# Data‐driven guidelines for phylogenomic analyses using SNP data

**DOI:** 10.1002/aps3.11611

**Published:** 2024-08-09

**Authors:** Jacob S. Suissa, Gisel Y. De La Cerda, Leland C. Graber, Chloe Jelley, David Wickell, Heather R. Phillips, Ayress D. Grinage, Corrie S. Moreau, Chelsea D. Specht, Jeff J. Doyle, Jacob B. Landis

**Affiliations:** ^1^ Department of Ecology and Evolutionary Biology University of Tennessee at Knoxville Knoxville Tennessee USA; ^2^ School of Integrative Plant Science, Section of Plant Biology and the L. H. Bailey Hortorium Cornell University Ithaca New York USA; ^3^ Department of Entomology Cornell University Ithaca New York USA; ^4^ Boyce Thompson Institute Ithaca New York USA; ^5^ Department of Ecology and Evolutionary Biology Cornell University Ithaca New York USA; ^6^ BTI Computational Biology Center, Boyce Thompson Institute Ithaca New York USA

**Keywords:** ancestral state reconstructions, divergence time estimation, genotyping‐by‐sequencing (GBS), *Glycine*, locus, phylogenetic comparative methods, single‐nucleotide polymorphism (SNP) filtering

## Abstract

**Premise:**

There is a general lack of consensus on the best practices for filtering of single‐nucleotide polymorphisms (SNPs) and whether it is better to use SNPs or include flanking regions (full “locus”) in phylogenomic analyses and subsequent comparative methods.

**Methods:**

Using genotyping‐by‐sequencing data from 22 *Glycine* species, we assessed the effects of SNP vs. locus usage and SNP retention stringency. We compared branch length, node support, and divergence time estimation across 16 datasets with varying amounts of missing data and total size.

**Results:**

Our results revealed five aspects of phylogenomic data usage that may be generally applicable: (1) tree topology is largely congruent across analyses; (2) filtering strictly for SNP retention (e.g., 90–100%) reduces support and can alter some inferred relationships; (3) absolute branch lengths vary by two orders of magnitude between SNP and locus datasets; (4) data type and branch length variation have little effect on divergence time estimation; and (5) phylograms alter the estimation of ancestral states and rates of morphological evolution.

**Discussion:**

Using SNP or locus datasets does not alter phylogenetic inference significantly, unless researchers want or need to use absolute branch lengths. We recommend against using excessive filtering thresholds for SNP retention to reduce the risk of producing inconsistent topologies and generating low support.

With the increased efficiency of high‐throughput sequencing, generating genome‐wide data for phylogenomic analysis is becoming cheaper and more feasible (Young and Gillung, 2020). There are many different approaches currently used to generate genome‐wide sequence data, each with required inputs and resulting limitations (Chambers et al., 2023). Both wet lab (e.g., access to living tissue, difficulty in DNA extractions, and laboratory costs) and computational requirements (e.g., reference genome, probe design, ultimate bioinformatic investment) need to be considered before starting any comprehensive phylogenomics project (McKain et al., 2018; Dodsworth et al., 2019).

While many phylogenomic methods rely on input data that include relatively long gene sequences, some types of sequencing data are not easily obtained for large numbers of samples, especially in non‐model systems (Dodsworth et al., 2019). For example, single‐nucleotide polymorphisms (SNPs) are useful for phylogenomic studies in part due to the ease of data collection (i.e., produced via genome skimming, restriction site–associated DNA sequencing [RADSeq], or genome resequencing) and the ability to attain SNP datasets from a variety of species that cover a broad distribution across the genome (Leaché and Oaks, 2017). However, the effects of SNP vs. locus datasets on tree topology, branch lengths, and nodal support are poorly understood, and the applicability of using inferred trees from SNPs for downstream phylogenetic comparative methods (PCMs) has been questioned (Leaché et al., 2015).

While there is no shortage of recommendations for Hyb‐Seq phylogenomic studies (Weitemier et al., 2014; Kadlec et al., 2017; McKain et al., 2018; Villaverde et al., 2018) or using SNP‐based methods—often generated with RADSeq or other genotyping‐by‐sequencing (GBS) methods (Lee et al., 2014; Hyun et al., 2020, 2021)—there is little overlap in studies comparing results from the different approaches. One such comparison by Zhou and Xiang (2022) found topological congruence and consistency in divergence time estimation in their SNP vs. RAD‐locus (SNPs plus the flanking regions of the sequencing reads) datasets. However, congruence is affected when SNP sites and the surrounding flanking regions are treated as genes/loci in coalescent approaches such as Astral (Mirarab et al., 2014b). This leads to considerable incongruence across loci and sites primarily due to the lack of signal in the gene tree, as usually inferred by either RAxML (Stamatakis, 2014) or IQ‐TREE (Minh et al., 2020). In coalescent approaches such as SVDquartets (Chifman and Kubatko, 2014), each nucleotide is considered individually so any invariant flanking region is not relevant given the lack of phylogenetically informative characters. For these reasons, further evaluation of how different kinds of datasets impact phylogenomic inference and the results of downstream PCMs is necessary.

Another long‐standing issue in phylogenomic inference is the effect of missing data. Given the complexity and scale of genome‐wide sequencing projects, it is almost certain that datasets will be affected by some degree of missing information, either as a result of the way that samples are collected and stored (e.g., herbarium vs. silica‐dried tissue) or due to variation in the accuracy of sequencing technology. This raises the important question of how missing data affect the accuracy and reliability of phylogenetic inference on a genomic scale. Historically, conflicting viewpoints exist regarding the influence of missing data on phylogenetic inference.

One side suggests that missing data has not been demonstrated to have a direct impact on phylogenetic inference, especially when datasets are large and the amount of missing data is relatively low (Wiens, 2003, 2006; Philippe et al., 2004). For instance, Wiens (2003) and Roure et al. (2013) showed that even with moderate amounts of missing loci, the resulting phylogenetic trees were still highly congruent with those obtained from complete datasets. Many of these phylogenetic studies have used genes that were neutral, mostly neutral, or under stabilizing selection (Edwards, 2009), instead of more rapidly evolving SNPs (Morin et al., 2004), which may or may not be under strong selection (Pavlidis and Alachiotis, 2017). A notable example of missing data in a SNP dataset is the successful resolution of a recent radiation event in Acanthaceae using RADSeq with as much as 90% missing data with more than 300,000 retained SNPs (Tripp et al., 2017).

However, other studies demonstrate that missing data can have a negative effect on phylogenetic inference by exacerbating uncertainty in inferred topologies, potentially leading to the estimation of misleading branch lengths (Huelsenbeck, 1991; Lemmon et al., 2009). One study even found that 70% data completeness was necessary to avoid spurious relationships (Smith et al., 2020). These studies contend that missing data can introduce biases, affecting the accuracy and reliability of the inferred phylogenetic relationships. Consequently, these uncertainties may propagate further in downstream analyses, potentially compromising the interpretation of evolutionary processes in both concatenation approaches and species tree inferences that rely on previously inferred gene trees (Mirarab et al., 2014a; Springer and Gatesy, 2016; Nute et al., 2018). In light of previous contradicting studies, the effect of missing data on phylogenetic inference remains a topic of debate, and we currently lack guidelines for best practices.

To address these issues and explore the effects of SNP vs. locus use on phylogenomic analyses—including the effects of SNP filtering stringency when a given SNP is kept or removed and the relation of this filtering to the generation or elimination of missing data—we conducted tree inference and downstream PCMs on the flowering plant genus *Glycine* Willd. (Fabaceae). The genus *Glycine* includes the cultivated soybean (*G. max* (L.) Merr.) and its wild East Asian annual progenitor (*G. soja* Siebold & Zucc.) as well as a group of approximately 26 perennial species mostly native to Australia (Sherman‐Broyles et al., 2014b; Landis and Doyle, 2023). Diploid *Glycine* species are largely inbreeding, with low levels of heterozygosity. Perennial *Glycine* species are classified in “genome groups” (Singh and Hymowitz, 1985; Hymowitz et al., 1998); originally based on artificial crossing studies, these have been refined with molecular phylogenetic data (Sherman‐Broyles et al., 2014a).

In this study, we use GBS data from 22 diploid *Glycine* species to investigate the effects of data type (SNP vs. locus) and SNP retention stringency for common phylogenetic inference and downstream PCMs. Specifically, we set out to compare the effects of SNPs vs. entire RAD loci on overall inferred tree topology, node support, branch length, node age, and downstream PCMs. Through our analyses, we provide support for researchers in deciding the best phylogenomic practices in their study system when using genome‐wide data.

## METHODS

### Taxonomic sampling and SNP vs. locus identification

The GBS data used in this study were previously described in Landis and Doyle (2023) and were generated from 22 accessions, each representing a named or informally recognized perennial *Glycine* species (Table 1). Briefly, DNA was extracted from tissue grown from seed stocks obtained from CSIRO (Commonwealth Scientific and Industrial Research Organization, Australia) using a cetyltrimethylammonium bromide (CTAB) method (Doyle and Doyle, 1987). Extracted DNA was sent to the University of Wisconsin Biotechnology Center (Madison, Wisconsin, USA) for GBS library preparation following Elshire et al. (2011) using the *ApeK1* restriction enzyme. Libraries were sequenced on an Illumina HiSeq 2000 (Illumina, San Diego, California, USA) at the Cornell Institute of Biotechnology (Ithaca, New York, USA) with a single‐end 100‐bp approach. Reads were demultiplexed using the process_radtags Perl script in Stacks v2.55 (Rochette et al., 2019). Accession numbers for the Sequence Read Archive (SRA), number of reads, and which genome group each accession belongs to can be found in Table 1. Because a de novo SNP‐calling approach was undertaken instead of a reference‐guided approach, raw reads were cleaned using fastp v0.12.4 (Chen et al., 2018) with default parameters and the specification to automatically detect adapters, require a minimum quality score of 20, and require a minimum read length of 60 bp for reads to pass filtering.

**Table 1:** Accessions used in this study, including number of reads, estimated depth after SNP calling, number of loci assembled within each accession, and the known genome group.

Species	SRA no.	No. of reads	Estimated depth	Assembled loci	Genome group
*Glycine albicans*	SRR18315602	3,319,398	18.92×	177,436	I genome
*Glycine arenaria*	SRR18315601	1,416,973	16.20×	88,094	H genome
*Glycine argyrea*	SRR18315591	3,133,554	22.07×	143,284	A genome
*Glycine canescens*	SRR18315580	2,409,732	17.95×	135,312	A genome
*Glycine clandestina*	SRR18315575	5,317,539	32.37×	166,063	A genome
*Glycine* sp. *“cracens”*	SRR18315595	3,204,690	21.30×	152,150	B genome
*Glycine falcata*	SRR18315574	687,974	11.00×	63,079	F genome
*Glycine gracei*	SRR18315573	2,707,429	19.52×	139,795	A genome
*Glycine hirticaulis*	SRR18315572	1,491,560	15.15×	99,371	H genome
*Glycine lactovirens*	SRR18315571	3,229,367	20.42×	160,799	I genome
*Glycine latifolia*	SRR18315600	2,240,664	17.65×	128,166	B genome
*Glycine microphylla*	SRR18315599	1,825,974	16.03×	114,992	B genome
*Glycine pindanica*	SRR18315598	1,972,682	16.74×	118,742	H genome
*Glycine pullenii*	SRR18315597	1,151,892	13.25×	87,740	H genome
*Glycine stenophita*	SRR18315592	2,434,744	20.42×	119,952	B genome
*Glycine syndetika*	SRR18315589	3,883,250	21.99×	178,332	A genome
*Glycine tomentella* D1	SRR18315587	3,595,114	20.78×	175,600	E genome
*Glycine tomentella* D2	SRR18315586	2,240,510	13.58×	166,821	E genome
*Glycine tomentella* D3	SRR18315585	3,315,188	19.67×	170,003	D genome
*Glycine tomentella* D5A	SRR18315581	2,861,702	19.53×	148,274	Ha genome
*Glycine tomentella* D5B	SRR18315579	2,461,459	20.15×	123,171	H genome
*Glycine* sp. *“wilsonii”*	SRR18315593	3,884,757	24.91×	157,579	B genome

*Note*: SRA = Sequence Read Archive.

The denovo_map.pl script in Stacks v2.62 (Rochette et al., 2019) was used to de novo call SNPs with default parameters with the inclusion of –force‐diff‐len in ustacks to account for a slight variation in read lengths across samples due to different barcode sizes. Specifically for SNP identification, the default value for the minimum stack depth (‐m 3) was used, meaning that at least three reads were needed to determine an allele. The distance between stacks (‐M 2) was used to minimize combining repetitive regions together into one locus. The populations module was then used with different filtering criteria for SNP retention, specifically, the proportion of individuals in which a shared SNP site must be present to be retained, which takes into account both the presence of polymorphisms and missing data at that site (*r* = 0%, at least one individual; 15%, at least three individuals; 30%, at least six individuals; 45%, at least nine individuals; 60%, at least 13 individuals; 75%, at least 16 individuals; 90%, at least 19 individuals; and 100%, all 22 individuals), plus the additional filtering parameters of minimum minor allele frequency (MAF) = 0.05, minimum minor allele count (MAC) = 3, and maximum observed heterozygosity = 0.5. Using this filtering scheme for SNP retention, when *r* = 15% any given SNP needed to be found in a minimum of three individuals, with all other individuals allowed to either have the SNP or have no genetic data at that site. Given the additional filtering parameters of MAC and MAF, the absolute minimum for the number of individuals exhibiting polymorphisms was two (minimum of three minor alleles). When *r* = 100%, all 22 individuals must possess the SNP, and if any individual was missing genetic data that SNP was removed entirely from the dataset. For the eight datasets, variant (SNP only) and full loci (SNP + flanking regions) were exported in PHYLIP format using the –phylip‐var‐all and –phylip‐var commands in the populations module. The exported PHYLIP files from Stacks were converted to FASTA format for Bayesian analysis (see below) with AliView v1.28 (Larsson, 2014). The amount of missing data per accession in each dataset was calculated in VCFtools v0.1.16 (Danecek et al., 2011) from the VCF file that was generated alongside the PHYLIP alignments by the Stacks populations module. Example alignments for variant sites only for all eight SNP filtering retention thresholds of the *Glycine* data are shown in Figure S1 in Appendix S1.

As a comparison to the empirical data, GBS data for 12 individuals (12 populations with one individual per population) were simulated with radinitio v1.2.1 (Rivera‐Colón et al., 2021) using the 20 chromosomes of the *Glycine max* Williams 82 v4 reference genome (Schmutz et al., 2010). SNPs were called de novo from the simulated reads for the 12 individuals and filtered using the same parameters as the *Glycine* GBS data.

### Phylogenetic inference

Maximum likelihood trees were inferred with RAxML‐NG v1.1.0 (Kozlov et al., 2019) with 100 bootstraps, the GTR+G model of molecular evolution, and specifying *G. falcata* Benth. as the outgroup, as demonstrated by previous nuclear studies showing *G. falcata* sister to the rest of the perennial species (Hwang et al., 2019; Zhuang et al., 2022; Landis and Doyle, 2023). No partitioning was done in the full locus dataset to make comparisons between variant and full locus as equitable as possible. Datasets with different levels of SNP retention (i.e., the number of individuals that a SNP must be present in to be kept: 0%, at least one individual; 15%, at least three individuals; 30%, at least six individuals; 45%, at least nine individuals; 60%, at least 13 individuals; 75%, at least 16 individuals; 90%, at least 19 individuals; and 100%, all 22 individuals), as well as variant sites only (SNPs) and locus (variant sites plus sequenced flanking regions in the full RAD locus), were used to infer maximum likelihood trees. Missing genetic information at any given site was coded with an N because Ns, gaps, and missing data are treated the same in RAxML (Kozlov et al., 2019). The same steps and parameters were followed for the simulated GBS data.

To explore whether differences in topology and nodal support could be explained by SNP filtering strategy alone, or by a combination of filtering strategy and alignment size, the SNP alignments for each threshold were downsampled to produce four nonoverlapping alignments of 2491 bp (the size of the 100% SNP alignment). For each of the four subsampled alignments, a maximum likelihood tree was inferred with 100 bootstrap replicates.

The RAxML topologies inferred for each of the eight different SNP retention levels for both the empirical *Glycine* (rooted with *G. falcata*) and simulated (rooted with msp00) GBS data (32 topologies total) were used as inputs into phytools v1.9‐16 (Revell, 2012) to determine the number of unique topologies recovered using the *find.unique* function as described on the phytools blog (http://blog.phytools.org/2016/05/identifying-unique-tree-topologies-in.html).

### Divergence time estimation

Bayesian divergence time estimation was performed with BEAST v2.7.2 (Bouckaert et al., 2019) using a secondary calibration based on a previous dating analysis of *Glycine* (Zhuang et al., 2022) incorporating the split of the *Glycine* and *Phaseolus* L. lineages ~22–37 mya (Koenen et al., 2021). The following settings were used in generating the BEAUTi file: ambiguities allowed, estimate substitution rate, gamma category count of 4, estimate gamma shape, GTR substitution model with empirical base frequencies, optimized relaxed clock, and birth–death tree prior. A calibration point forcing a monophyletic clade of all *Glycine* species with a median value of 6.12 and a sigma of 0.515 with a normal distribution was set. A total of 100–500 million Markov chain Monte Carlo (MCMC) generations were performed, sampling every 5000 generations or until the effective sample size values were over 200 as checked for convergence with Tracer v1.7.2 (Rambaut et al., 2018). An initial comparison between runs using a Yule tree prior or a birth–death tree prior showed overlapping age estimates for all nodes observed; therefore, all resulting analyses were performed using the birth–death tree prior model.

Maximum likelihood rate smoothing analyses are often preferred with large datasets to convert the inferred phylogeny to an ultrametric tree (Ho and Duchêne, 2014; Barba‐Montoya et al., 2021; Costa et al., 2022), primarily due to their increased speed and ability to be used with limited computational resources. RelTime, which is based on the relative rate framework (Tamura et al., 2012, 2018) and implemented in MEGA11 (Tamura et al., 2021), was used as a comparison to the Bayesian approach. Not only can RelTime be executed a thousand times faster than Bayesian methods (Tamura et al., 2012), but RelTime estimates have also been shown to be consistently more accurate than other maximum likelihood methods such as TreePL and least‐squares dating (Barba‐Montoya et al., 2021). To investigate the impacts of SNP vs. loci datasets, as well as the impact of SNP retention filtering, on downstream divergence time estimates, we dated each of the phylogenies inferred in RAxML, with the exception of the largest alignment file consisting of 111 million sites due to computational limitations. We used an equivalent calibration point to that in the BEAST analyses, setting the age of the most recent common ancestor of *Glycine* to a mean of 6.12 mya, with a standard deviation of 0.435 to span the confidence interval of 5.27–6.97 mya. A normal distribution for this calibration point was used, along with the GTR model of molecular evolution with a gamma rate. Differences between inferred nodes were compared at the same data filtering level (missing data) between variant‐only and locus data. A paired Student's *t*‐test (Student, 1908) was used to identify significant differences in inferred ages, with a Bonferroni correction for multiple tests.

### Node age by data type and filtering stringency

To explore the relationship between node age and SNP filtering stringency, we selected the mean node age for a set of selected nodes across the phylogeny for each filtered dataset. Using ggplot2 in R (Wickham, 2016), we plotted mean node age and standard error across each of the selected nodes. We also conducted linear regressions to test for significant trends between node age and filtering stringency, using the *lm* function in R.

### Comparison of filtering and data type on branch lengths and nodal support

Branch lengths and node support were compared between data types (SNP vs. locus) and SNP filtering stringency. These analyses were only conducted on RAxML trees because BEAST tree branch lengths and node ages were all standardized to unit time. To compare branch lengths between locus and SNP trees across different filtering parameters, all RAxML trees from the *Glycine* GBS data and the simulated data were read into R using the *read.tree* function in ape v5.7‐1 (Paradis and Schliep, 2019). Branch lengths, node support, node number, SNP retention stringency, and data type were extracted from each tree and aggregated into a dataframe using a custom script in R v4.2.2 (R Core Team, 2021). Boxplots of branch lengths and node support were compared within data type across filtering stringencies using the *geom_boxplot* function in ggplot2 v3.4.2 (Wickham, 2016). Pairwise comparisons of branch lengths and nodal support at different SNP retention levels within datasets were made using a Wilcoxon signed‐rank test with a Bonferroni correction with continuity correction using the *pairwise.wilcox.test* function in R. To further investigate branch length differences, phylogenetic trees were inferred in RAxML‐NG using Felsenstein's method for ascertainment bias correction (+ASC_FELS{w}) by specifying the number of invariant sites {w}. All invariant sites, including ambiguities, were removed from the SNP‐only alignments of the different filtering thresholds using IQTREE2 v2.2.7 (Minh et al., 2020) with the +ASC flag to produce variant sites–only alignments. The number of invariant sites for each filtering threshold was the total number of bases in the locus alignments minus the number of bases in the alignments produced by IQTREE2.

### Ancestral character estimation

A downstream approach that is a fundamental tool of comparative phylogenetic methods is ancestral character state estimation (Felsenstein, 1985). Different approaches for this have been reviewed in other studies, but all are conducted after a tree is inferred (Holland et al., 2020). The preferred approach in most studies is to use chronograms where branch lengths are in units of time; however, this is not consistent across the literature. Litsios and Salamin (2012) recommend using the tree (chronogram or phylogram) that produces the higher phylogenetic signal, while Cusimano and Renner (2014) recommend performing ancestral character state reconstructions on multiple different branch length representations and selecting the option with the fewest inferred steps. Because the alternative options to using a chronogram vary between studies and data, we used stochastic character mapping with a simulated binary trait on the inferred phylograms and chronograms using the 45% SNP retention threshold (SNPs were present in at least nine of 22 individuals) datasets as a representative dataset to see if differences were observable in number of transitions, time spent in different states, and percentage of traits across all nodes due to the trees exhibiting largely different branch lengths (see below). Specifically, a binary trait was randomly simulated across the tips without taking into account the phylogenetic position of samples using the *RAND()* function in Excel to generate a random number between 0 and 1, followed by rounding to the nearest whole number to represent presence or absence of the trait. Stochastic mapping was performed in phytools v1.5‐1 (Revell, 2012) with *make.simmap* using the all rates different (ARD) model and 1000 simulations.

We also explored how tree and data type affect ancestral character state estimations of continuous variables. To do this, we first randomly simulated a continuous trait using a random number generator from 0 to 1. After simulating these continuous data, we then estimated ancestral character states across the same four selected trees for the discrete traits mentioned above using the *anc.mL* function under a Brownian motion model in phytools v1.5‐1, using the same trees as was done for stochastic mapping. Observed and reconstructed values of the simulated continuous trait were projected onto the edges of the respective phylogenies using the function *contMap* in phytools v1.5‐1.

## RESULTS

### SNPs vs. locus information

Based on the required threshold of the percentage of accessions in which a SNP must be found to be retained in a dataset (i.e., SNP retention), from 0% (allowing for a SNP at any site in any given accession) to 100% (a given SNP site must be shared across all accessions), the number of included RAD loci varied from 1960 (100%) to 1,339,951 (0%) (Table 2). Across all RAD loci, the number of variant sites ranged from 2491 to 118,925; when the full locus was taken into account, the total alignment size ranged from 182,544 bp to 111,994,953 bp. The mean fraction of missing data for just the variant sites across all individuals in a filtering threshold ranged from zero to 0.522, while the mean level of missing data in the full locus ranged from 0.099 to 0.906. Missing data in any given accession across datasets is shown in Appendix S2, showing that most accessions were similar in terms of the amount of missing data, except for *G. falcata*, which showed substantially more missing sites than other accessions.

**Table 2:** Summary of the different datasets, including the fraction of shared sites a SNP must have to be retained, number of loci for each filtering scheme, the number of base pairs for variant and locus datasets, and the median and mean values of missing data for each dataset.

Shared percentage required	Loci	Variant sites (bp)	All sites (bp)	Median missing variant	Mean missing variant	Median missing locus	Mean missing locus
100	1960	2491	182,544	0.000	0.000	0.115	0.099
90	6522	9125	606,786	0.033	0.050	0.133	0.140
75	13,305	19,956	1,236,145	0.092	0.121	0.178	0.200
60	22,713	33,340	2,102,501	0.162	0.201	0.248	0.273
45	43,490	58,081	3,983,546	0.273	0.322	0.359	0.392
30	76,196	85,371	6,906,941	0.378	0.423	0.483	0.507
15	170,505	117,310	15,060,214	0.483	0.518	0.649	0.663
0	1,339,951	118,925	111,994,953	0.489	0.522	0.904	0.906

### Topology

**Figure 1:**
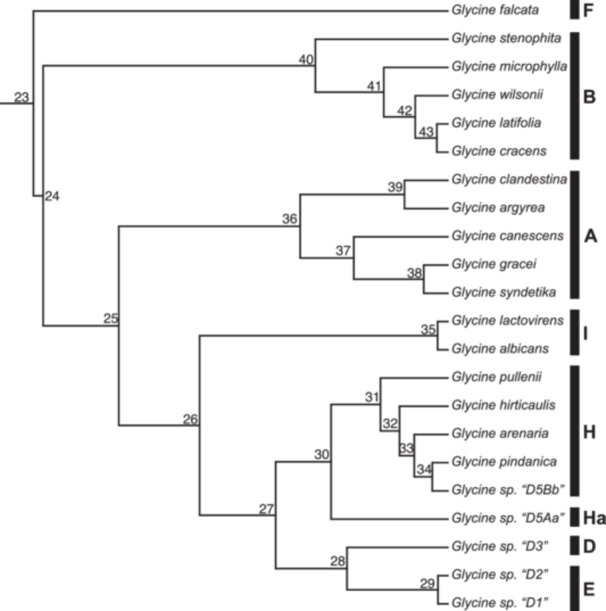
Chronogram of the SNP phylogeny, which is largely congruent across different data types and filtering parameters. The only observable differences are when SNP filtering is too extreme and only sites shared across all taxa are kept. The bars next to the tips represent the assigned genome group for each species, and the numbers next to the nodes represent nodes plotted in Figure 3 and not support values.

The inferred topologies across all 16 datasets were nearly identical, regardless of whether the alignment was inferred with SNPs or the full locus (see Figure S2 in Appendix S1, Appendix S3). The five genome groups represented by multiple individuals were all recovered as monophyletic. Even though *G. falcata* (F‐genome group) had the most missing data of any sample, when rooting with *G. falcata* the other genome groups displayed the same relationships regardless of filtering threshold. The D‐genome and E‐genome groups were sister to each other, and were in turn sister to a clade formed by the H‐ and Ha‐genome groups. This resulting clade was successively sister to the I‐genome, A‐genome, and B‐genome groups. The phytools clustering showed three unique tree topologies across the 16 trees, with the RAxML and BEAST trees showing the exact same patterns. The two most prevalent topologies (topology 1 found in eight trees and topology 2 found in six trees) only differ within the H‐genome group (Figure 1, Figure S2 in Appendix S1), specifically with the placement of *G. arenaria* Tindale, *G. hirticaulis* Tindale & Craven, *G. pindanica* Tindale & Craven, and *G. tomentella* Hayata D5B. In both topologies, *G. pullenii* B. E. Pfeil, Tindale & Craven is sister to the remaining species. In topology 1, *G. hirticaulis* is sister to the clade of *G. arenaria*, *G. pindanica*, and *G. tomentella* D5B, whereas in topology 2, *G. hirticaulis* is sister to *G. arenaria*. The support for the placement of *G. arenaria* is generally below the threshold of support (<70%). The third topology (topology 3), found only in the trees inferred in the 100% SNP retention threshold (SNPs shared across all 22 individuals) for both SNP and locus, again differs in the H‐genome group with additional uncertainty involving *G. pullenii*, with the species no longer being well supported as sister to the rest of the clade but instead sister to *G. pindanica* and *G. tomentella* D5B with low support (49% bootstrap support in the SNP tree and 29% in the locus tree). The observed topology in the 100% SNP retention threshold was observed in one of the 28 downsampled trees, specifically one of the four 90% SNP retention threshold trees (Appendix S4), providing further support that this topology is inconsistent with the most frequently recovered topologies and is only found with more extreme filtering.

The topological comparisons for the simulated data were more varied, with seven total topologies; the increased number was mostly due to the lack of support in inferred relationships. One result congruent with the empirical data is that the inferred topologies for the 100% SNP retention threshold were unique and not found in any other dataset. The most consistent topology was found in the 45–75% (SNPs found in at least 9–16 of the 22 individuals) filtering thresholds, with five of the 16 trees showing this topology.

### Comparison of filtering and data type on branch lengths and node support

**Figure 2:**
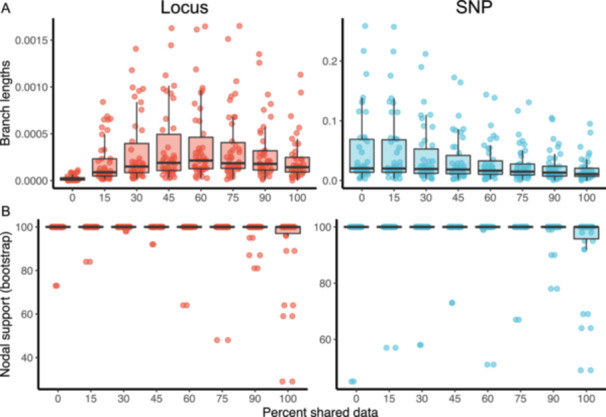
Summary statistics for inferred RAxML trees associated with branch length and node support across different filtering thresholds. (A) Comparison of branch lengths between SNP and locus datasets by filtering stringency. (B) Comparison of nodal support between SNP and locus datasets by filtering stringency.

Branch lengths and node support were only compared within data type (i.e., between filtering stages in SNP and locus). While data on branch lengths and node support were not statistically compared between data types, one noticeable difference is that raw branch lengths of locus data were in general two orders of magnitude shorter than those of SNP data (Figure 2A). Branch lengths for the locus and SNP datasets were not normally distributed (Figure 2A; Shapiro–Wilk's test of normality, *P* value < 0.01). However, across the locus dataset, the lowest branch lengths occurred at 0% and 100% shared thresholds, and the largest occurred between 30–75% (SNPs found in at least 6–16 individuals). Alternatively, within the SNP data, branch lengths were longest in the dataset with the 0% SNP filtering threshold (allowing for a unique polymorphism in only one accession) and shortest in the dataset with 100% SNP filtering threshold (where a SNP was required to be shared across all individuals; Figure 2A). Based on a Wilcoxon signed‐rank test with a Bonferroni correction, node support was not significantly different across filtering stringencies either within the SNP or locus data (Appendix S5); however, there were differences in branch lengths across filtering stringencies (Figure 2A). Within the SNP data, significant differences in branch lengths based on a Wilcoxon signed‐rank test occurred between filtering stringencies 0% and 75%, 0% and 100%, 15% and 30%, and 15% and 100%. Within locus data, significant differences in branch lengths based on a Wilcoxon signed‐rank test occurred between filtering stringencies 0% and 15%, 0% and 30%, 0% and 45%, 0% and 60%, 0% and 75%, 0% and 100%, 15% and 60%, 15% and 75%, and 60% and 100% (Appendix S5; *P* < 0.05 with Bonferroni correction). Using the Felsenstein ascertainment bias correction model in RAxML‐NG, branch length differences between the SNP and locus datasets at the same filtering threshold were resolved.

Node support in the empirical datasets was generally quite high, regardless of the SNP filtering threshold used. Downsampling the alignments of less stringent filtering thresholds to the size of the 100% threshold (2491 bp) resulted in a decrease in node support for some nodes (Figure S3 in Appendix S1) compared to the full datasets (Figure 2B). However, most nodes still maintained 100% bootstrap support, with most variation in support occurring at lower filtering thresholds.

Comparing branch length and nodal support in the simulated data between data types and across SNP retention thresholds showed similar patterns to the empirical data. As the SNP retention threshold became more conservative (from 0% to 100%), the branch lengths became shorter for both the SNP and locus datasets. Based on a Wilcoxon signed‐rank test with a Bonferroni correction, the branch lengths of 100% in the locus data were significantly different from all other thresholds (except for 60% with a *P* value = 0.054), while there were no significant differences in the SNP trees. Nodal support fluctuated in the simulated data across SNP retention thresholds, with the highest median values observed in the 60–90% thresholds. Node support was significantly lower at the 100% threshold, for both SNP and locus, than any other threshold (Figure S4 in Appendix S1) based on a Wilcoxon signed‐rank test.

### Divergence time estimation

**Figure 3:**
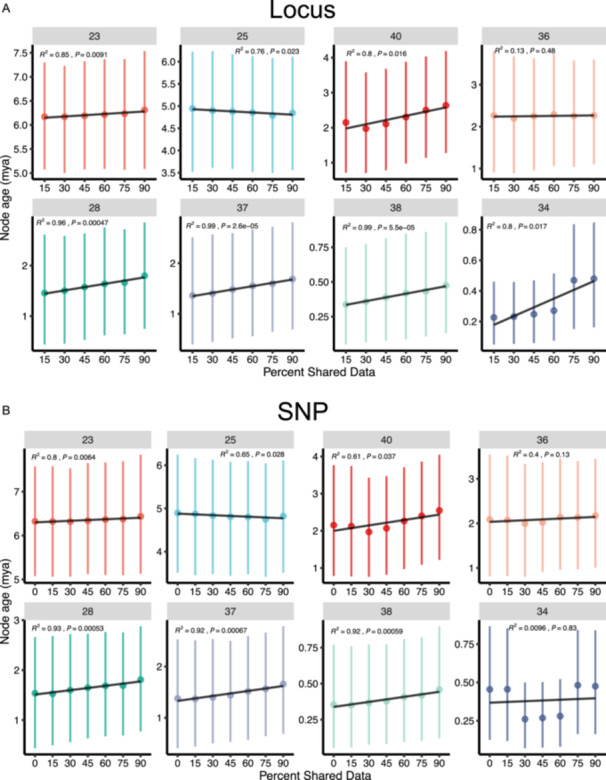
Selected nodes from the node ages determined from the BEAST analyses for (A) locus‐inferred phylogenies and (B) SNP‐based phylogenies. Mean node ages are represented by a point, with 95% confidence intervals of absolute age represented by vertical lines. Linear regressions depict the relationship between mean node age and filtering stringency. Numbers above each inset represent a node number corresponding to Figure 1.

The BEAST analyses within data type produced congruent results in terms of estimated node age across different SNP retention thresholds, with the 95% confidence interval for selected nodes almost entirely overlapping (Figure 3). The one exception to this was node 34, which is in the H‐genome group and is the most recent common ancestor of *G. pindanica* and *G. tomentella* D5Bb. As noted above, the relationships within the H‐genome group change based on different retention thresholds, with some relationships within the clade no longer being well supported. Specifically, node 34 is the only node that was observed to change relationships, albeit with low support, between topology 1 and topology 2 (Figure S2 in Appendix S1). For some nodes, a significant correlation exists, such that as SNP retention stringency increases, the inferred divergence time increases; in other nodes, the opposite is true (Figure 3). Even though several of these changes are statistically significant, the differences are still within the 95% confidence interval of all inferred ages for that particular node across the different levels of filtering.

The inferred divergence times using RelTime were highly similar regardless of whether SNP or locus data were used. The inferred node ages of RelTime were largely within the 95% confidence interval generated in the BEAST analyses (Appendix S6), with larger variance as the SNP retention threshold approached 0%. Generally speaking, as the stringency for SNP retention increased, the mean difference between variant and locus marginally decreased. The mean difference in ages of the same node was 0.023 mya when all SNPs were required to be shared across all individuals (100%), increasing to 0.025 mya (90%), 0.036 mya (75%), 0.041 mya (60%), 0.038 mya (45%), 0.052 mya (30%), and 0.61 mya (15%). The observed differences between datasets were much smaller than the standard deviation of 0.435 mya used in the calibration point for dating (6.12 ± 0.435 mya). A paired Student's *t*‐test showed no significant difference in node ages between SNP and locus after Bonferonni correction (critical value 0.05/7), whereas two comparisons showed a significant difference in estimated node age at the 45% (*P* = 0.021) and 30% (*P* = 0.014) thresholds given a standard cutoff of 0.05 (Appendix S7).

### Ancestral character state estimation

**Figure 4:**
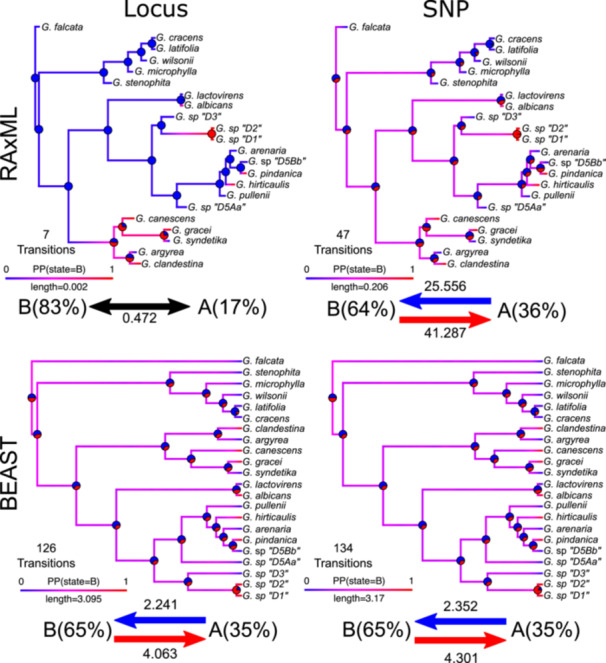
Summarized results of 1000 stochastic character maps of simulated data on two representative phylograms and chronograms from the 45% filtering stringency. Blue and red arrows represent transition rates. The percentages in parentheses are the total time spent in each state. Branches are colored by their posterior probability (PP) of being in state A (blue) or B (red). Pie charts represent the probability of nodes in state A (0) or B (1). The total number of transitions are denoted at the top of each legend.

Ancestral character state estimation differed qualitatively and quantitatively between the RAxML and BEAST trees. The RAxML SNP tree had a total of 47 transitions, spending 64% of time in state B and 36% of time in State A (Figure 4). Transition rates were 25.556 changes per unit branch length from A–B and 41.287 changes per unit branch length from B–A. The RAxML locus tree had a total of seven transitions, spending 83% of time in state B and 17% of time in State A. Transition rates were equal between State A and B (0.472 changes per unit branch length). The BEAST SNP tree had a total of 134 transitions, spending 65% of time in state B and 35% of time in State A. Transition rates were 2.352 changes per unit branch length from A–B and 4.301 changes per unit branch length from B–A. The BEAST locus tree had a total of 128 transitions, spending 65% of time in state B and 35% of time in State A. Transition rates were 2.241 changes per unit branch length from A–B and 4.063 changes per unit branch length from B–A.

Qualitatively, the ancestral states and proportion of time spent in each state appear to be relatively similar between the RAxML tree using SNP data and both stochastic character maps of the BEAST trees (Figure 4). Quantitatively, however, the transition rates for the RAxML SNP tree were 10.9 times higher and the total number of transitions was almost three times lower compared to the BEAST trees. The reconstruction with the RAxML locus tree produced the most different results compared to all other trees. The ancestral state estimation for almost every node was incongruent, the total number of transitions was 6.7 times less than the respective SNP tree and almost 20 times less than the BEAST trees, and the transition rates were equal between A–B and very low. Overall, using trees with branch lengths in proportion to the nucleotide substitution rate appears to provide drastically different quantitative and qualitative results compared to trees with branch lengths in proportion to absolute time.

**Figure 5:**
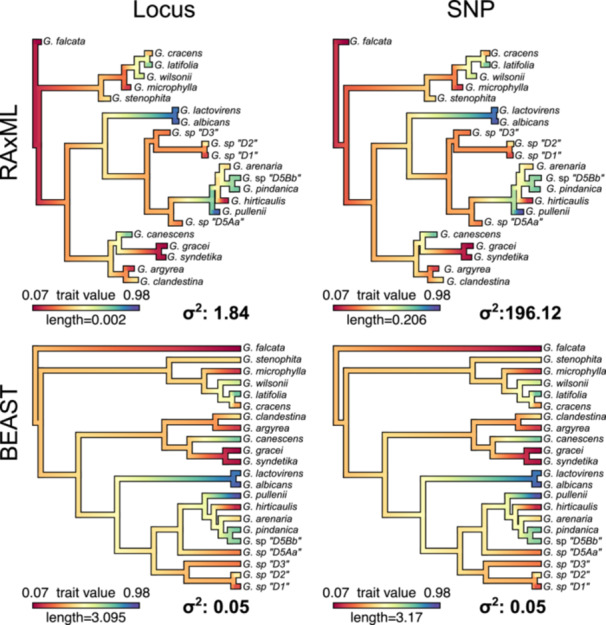
Summarized results of ancestral character estimation of continuous character states for two representative phylograms and chronograms from the 45% filtering stringency. Branches are colored by their estimated continuous state based on the associated legend. The σ^2^ represents the estimated Brownian motion parameter. The data were modeled with a σ^2^ equal to 1.

Similar disparities in ancestral state estimations were also observed in continuous variables (Figure 5). Broadly, there was virtually no difference in ancestral state estimations and model parameters between SNP and locus BEAST chronograms. However, there were large differences between both SNP and locus phylograms and between RAxML phylograms and BEAST chronograms. SNP‐based and locus‐based phylograms both estimated a root ancestral state near the upper limits of the modeled continuous variable. Moreover, the rate of variance (σ^2^) was two orders of magnitude greater in the locus phylogram compared to the SNP phylogram (3144.58 vs. 30.87). The root state of chronograms was estimated to be near the middle of the modeled continuous variable, and the rate of variance was much lower compared to the RAxML trees and was equal between the SNP and locus BEAST trees.

## DISCUSSION

### Topological congruence, branch lengths, and nodal support

The topology generated here (Figure 1) using a de novo SNP‐calling approach is congruent with the phylogeny presented in Landis and Doyle (2023), which used a reference genome approach for identifying SNPs. In comparison, both the de novo approach presented here and the reference‐guided approach generated a fully resolved phylogeny with strong support for the majority of relationships. This suggests that robust relationships can be inferred with or without a reference genome. Moreover, the topologies we generated de novo using either SNP or full locus datasets were almost entirely identical, with the only exceptions being the relationships within the H‐genome group, especially when retained SNPs were required to be present in all individuals (100%; Figure S2 in Appendix S1). However, these incongruent relationships generally showed low (<70%) bootstrap support.

A notable finding was the divergence between absolute branch lengths in the full locus and SNP datasets. Branch lengths in the full locus datasets were nearly two orders of magnitude shorter than the branch lengths in the SNP datasets (Figure 2A). We attribute these differences to the total number of sites per sequence included in the analysis, thus affecting the inferred rates of evolution and overall branch lengths. However, while the absolute branch lengths differed drastically, the relative branch lengths between datasets did not vary. Notably, patterns of filtering stringency on branch lengths differed between the SNP and locus datasets. In the SNP datasets, branch lengths tended to decrease with increasing SNP retention stringency (Figure 2A). However, in the locus datasets, branch lengths were shortest at 0% (allowing for unique SNPs in some accessions) and longest around 45–60% SNP retention (9–13 individuals; Figure 2A, Appendix S5). In the SNP‐only datasets, decreasing the SNP retention stringency led to longer branch lengths, while the SNP retention threshold in the entire locus datasets did not show a linear pattern (Figure 2A). Even with the large differences in absolute branch lengths between the SNP and full locus datasets, the inferred node ages were largely overlapping across filtering and data types (Figure 2) regardless of whether a Bayesian or maximum likelihood dating approach was used. We attribute this to a similarity in relative branch lengths between the SNP‐ and locus‐inferred phylogenies.

Some of the branch length discrepancies we observed might be alleviated with the incorporation of an ascertainment bias flag (Lewis, 2001; Leaché et al., 2015) as implemented in RAxML or IQ‐TREE. The correction for ascertainment bias impacts the calculated branch lengths because the variant sites are sampled in a non‐uniform manner and only including SNPs deviates from theoretical expectations in how bases change (Lachance and Tishkoff, 2013). In some cases, implementing this bias correction is not straightforward because nonvariant sites cannot be included in the alignment. We found that the easiest approach to removing constant sites, including those due to ambiguities, was in IQTREE2 with the +ASC flag, which produces alignments of only variant sites. However, the number of invariant sites does need to be counted, and this may be difficult for non‐model systems and genome‐wide data. This is especially evident with de novo SNP‐calling approaches unless explicit investigation of flanking regions at different filtering thresholds is carried out. In our empirical data, we see that by including the Felsenstein method for ascertainment bias, our inferred branch lengths are congruent between the SNP + ascertainment bias correction and locus datasets.

Within *Glycine*, heterozygosity is low due to high rates of selfing (Doyle et al., 2004). In highly outcrossing systems, high levels of heterozygosity may also cause issues in phylogenetic inference due to the way ambiguities are handled (e.g., treating ambiguities as one base or the other, or by ignoring ambiguities altogether). In these cases, using models of genotype evolution (eight possible genotype combinations and the rates of change between combinations) instead of models of molecular evolution (four possible bases and the rates of change between bases), as implemented in RAxML‐NG (Kozlov et al., 2019), may provide a more accurate inference, especially in terms of branch length estimation. One additional caveat to consider is the age of divergence between the focal species. SNPs have been shown to be useful for inferring relationships in species with divergence times up to 40–60 mya (such as in *Drosophila*) but may not provide robust results when divergence times approach 100 mya (Rubin et al., 2012) due to a decrease in recoverable loci between taxa with increasing divergence times. We have shown that SNP data are useful for *Glycine*, where the perennial species diverged from the annual *G. max* approximately 8 mya (Zhuang et al., 2022). Determining the crown age of focal taxa, either from primary fossil evidence or secondary phylogenetic observations, should be considered before undertaking a SNP‐based phylogenomic analysis.

### Divergence time estimation by percent shared data and locus type

Previous phylogenetic studies reconstructing one or more gene trees have shown that allowing for missing data can help resolve difficult relationships and increase node support because there may be biological explanations for these patterns of missing information (Wiens, 1998, 2006; Wiens and Moen, 2008). However, there is a risk that allowing more missing data may increase long‐branch attraction (Wiens, 2006). This risk is compounded when the missing data are not randomly distributed among loci (Xi et al., 2016). Yet, there are no consistent trends in branch length and missing data (Jiang et al., 2014). These patterns are amplified when using SNP data that are more rapidly evolving than neutral genes (Morin et al., 2004) or those under stabilizing selection (Edwards, 2009). In our datasets, we observed a significant inverse linear relationship between the SNP retention stringency and mean age across most nodes (Figure 3). In other words, most nodes showed increased mean ages with increased stringency (i.e., less missing data) of SNP retention (Figure 3). However, it is important to note that variation in mean age tended to be captured within the full confidence interval of the node estimation, demonstrating the importance of bounding age‐dependent hypotheses by confidence intervals (Figure 3). As such, while there is a correlation between filtering and mean age, this may not be phylogenetically meaningful on small time scales.

Several SNP‐based studies have shown that retaining sites with missing data (i.e., decreasing the SNP retention threshold) increased the support for phylogenetic relationships (Wagner et al., 2013; Hodel et al., 2017; Tripp et al., 2017). This is likely due to larger datasets containing more phylogenetically informative sites, as well as because excluding sites with missing data can mask regions of the genome with the highest mutation rates. For instance, a SNP‐based study of African frogs (*Afrixalus* spp.) showed that allowing approximately 60% missing data in a given site provided congruent tree inferences across datasets, whereas topological differences were observed when 80–90% missing data were permitted (Crotti et al., 2019). Our results support the notion that including sites with a lower required sampling proportion and, in some cases, more missing data (at least to a certain extent) is beneficial in phylogenomic reconstruction, and also allows a faster computational time, compared to all sites being retained. While the inferred topologies generally did not change between datasets (except in the most stringent SNP filtering criteria, which produced a minimally different unique topology; Figure S2 in Appendix S1), some variation in node support across filtering stringencies was observed. Specifically, the largest variation in support was found when a SNP was required to be retained in all individuals (100%) compared to all other thresholds (Figure 2B), especially in simulated data, which had overall lower support values (Figure S4 in Appendix S1). We also observed the smallest variation in node support when a SNP was required to be present in at least 30–45% of individuals, which is similar to the best‐case scenarios of Crotti et al. (2019).

### Phylogenetic comparative methods and downstream analyses

We demonstrated that using phylograms leads to drastically different ancestral character estimations between SNP and full locus datasets in both discrete and continuous characters compared to chronograms (Figures 4 and 5; also see Wilson et al., 2022). These patterns are especially obvious when examining transition rates, where rates of morphological evolution were higher in phylograms compared to chronograms (Figures 4 and 5). The only exception to this was the stochastic character maps for the locus‐inferred phylogram, which estimated significantly lower total rates and only seven total transitions across the tree. The true ancestral character history is almost always unknown, and so here we were more interested in precision rather than accuracy. As such, we found that the estimated evolutionary parameters were more consistent between SNP and locus chronograms compared to SNP and locus phylograms (Figures 4 and 5). This likely reflects the drastic differences in absolute branch lengths between SNP and locus phylograms (Figure 2A), which is eliminated in chronograms (i.e., branch lengths become proportional to time). Incorporating an ascertainment bias correction model partially fixes this issue; however, the inferred branch lengths are dependent on the total number of nonvariant sites, and different branch lengths would be recovered if the total number of bases from flanking regions surrounding SNPs were used or if genome size minus SNP variants were used. Converting to chronograms is not dependent on the number of variant or nonvariant sites in the analysis.

These downstream results are, however, hardly surprising given that rates of morphological change in phylograms are denoted as the number of morphological changes per nucleotide substitution per site. This rate does not make much biological sense, unless the tree was inferred with genes that underlie the morphological traits of interest and mutations in those genes are known to directly correlate with particular shifts in phenotype (Litsios and Salamin, 2012). Going against what is often considered “standard practice,” phylograms are still used in PCMs across a range of studies (Landis et al., 2016; Ickert‐Bond et al., 2020; Maletti et al., 2021; Mennecart et al., 2022; Jauregui‐Lazo et al., 2023; Chomicki et al., 2024). While this comparison does not definitively answer the question about whether chronograms or phylograms should be used (for alternative approaches, see Litsios and Salamin, 2012; Cusimano and Renner, 2014), we do highlight the caution that is needed in comparing ancestral state reconstructions across studies that use different types of phylogenetic trees given the vastly different number of transitions and transition rates observed in phylograms compared to chronograms. Given the differences and the sensitivity of modeled parameters to branch lengths, we recommend researchers use chronograms when conducting PCMs, especially considering the congruent results observed in the BEAST trees regardless of whether SNP or locus datasets were used (Figures 4 and 5).

### Guidelines

Many points should be considered when determining which sequencing method is most appropriate, including the number of taxa, taxon sampling, crown age, heterozygosity, and the biological question of interest; these factors should all be considered before any data are generated. If researchers determine that a SNP‐based approach is best, the results from our analyses suggest the following recommendations. (1) Researchers can use either SNPs or full loci (short‐read contigs) in their analyses to produce reliable inferences. This is supported by the equivalent results in our phylogenomic inference, including overall topology, divergence time estimation, and PCMs on chronograms. Incorporation of ascertainment bias correction models can resolve differences in absolute branch lengths observed between SNP and full loci; however, inferred branch lengths are fully dependent on the specified number of nonvariant sites. (2) Use moderate filtering in SNP retention (SNPs found in ~45–75% of individuals), as over‐filtering can lead to inconsistent branch lengths and, in some cases, topological differences. On the other hand, under‐filtering of SNPs increases computation time with minimal benefit to phylogenetic inference. (3) Use chronograms in downstream PCMs, as phylograms are highly sensitive to differences in absolute branch lengths, leading to variation in evolutionary metrics. While we anticipate new information to provide further insights and guidelines for researchers conducting phylogenomic analyses, we hope our framework can be a helpful resource for phylogenetic analyses in the genomics age and can influence some of the methodological decisions taken by future researchers.

## AUTHOR CONTRIBUTIONS

G.Y.D., L.C.G., A.D.G., C.J., H.R.P., D.W., J.S.S., and J.B.L. conceptually designed the project. J.S.S., G.Y.D., and J.B.L. conducted the analyses. J.S.S. and J.B.L. drafted the manuscript. All authors contributed to editing the final draft of the manuscript and approved the final version of the manuscript.

## Supporting information


**Appendix S1.** Supplemental figures for “Data‐driven guidelines for phylogenomic analyses using SNP data.”

**Appendix S2.** Amount of missing data for each accession in each dataset as calculated by VCFtools in the resulting VCF file.

**Appendix S3.** Inferred topologies from all empirical datasets including different filtering thresholds and analysis methods. The right side of the cophylo plot is the 45% RAxML tree as a reference. Bootstrap support or posterior probability at nodes are 100%/1.0 unless otherwise specified.

**Appendix S4.** Tree topologies as scored by the phytools “find.unique” function across filtering thresholds for the empirical maximum likelihood and Bayesian trees, simulated GBS data maximum likelihood trees, and downsampled maximum likelihood trees.

**Appendix S5.** Wilcoxon signed‐rank pairwise comparisons of branch length and node support for the different data types and filtering strategies.

**Appendix S6.** Inferred topologies and divergence time estimation with the 95% confidence interval of node ages from all empirical datasets including different filtering thresholds and analysis methods. Divergence time estimation in RelTree was rooted with *Glycine falcata* as in the BEAST analyses, but due to plotting limitations of Mega, the outgroup was not included in the plot.

**Appendix S7.** Comparisons of inferred divergence times from RelTime between the SNP and locus datasets.

## Data Availability

Raw sequencing reads are available on the Sequence Read Archive with the accession numbers presented in Table 1. Analyses and plotting scripts are available on GitHub: https://github.com/Suissajacob/SNPS_testing.
