# Supplementary material for: Data‐driven guidelines for phylogenomic analyses using SNP data
Source: Appl Plant Sci. 2024 Aug 9;12(6):e11611. doi: 10.1002/aps3.11611 (PMC11610416; doi:10.1002/aps3.11611)

DOI 10.1002/aps3.11611

Appendix S1. Supplemental figures for "Data-driven guidelines for phylogenomic analyses using SNP data."

Figure S1. Example alignments for the different SNP retention thresholds providing examples of the composition of each column in the alignment. Missing data and N's are treated the same as based on RAxML.

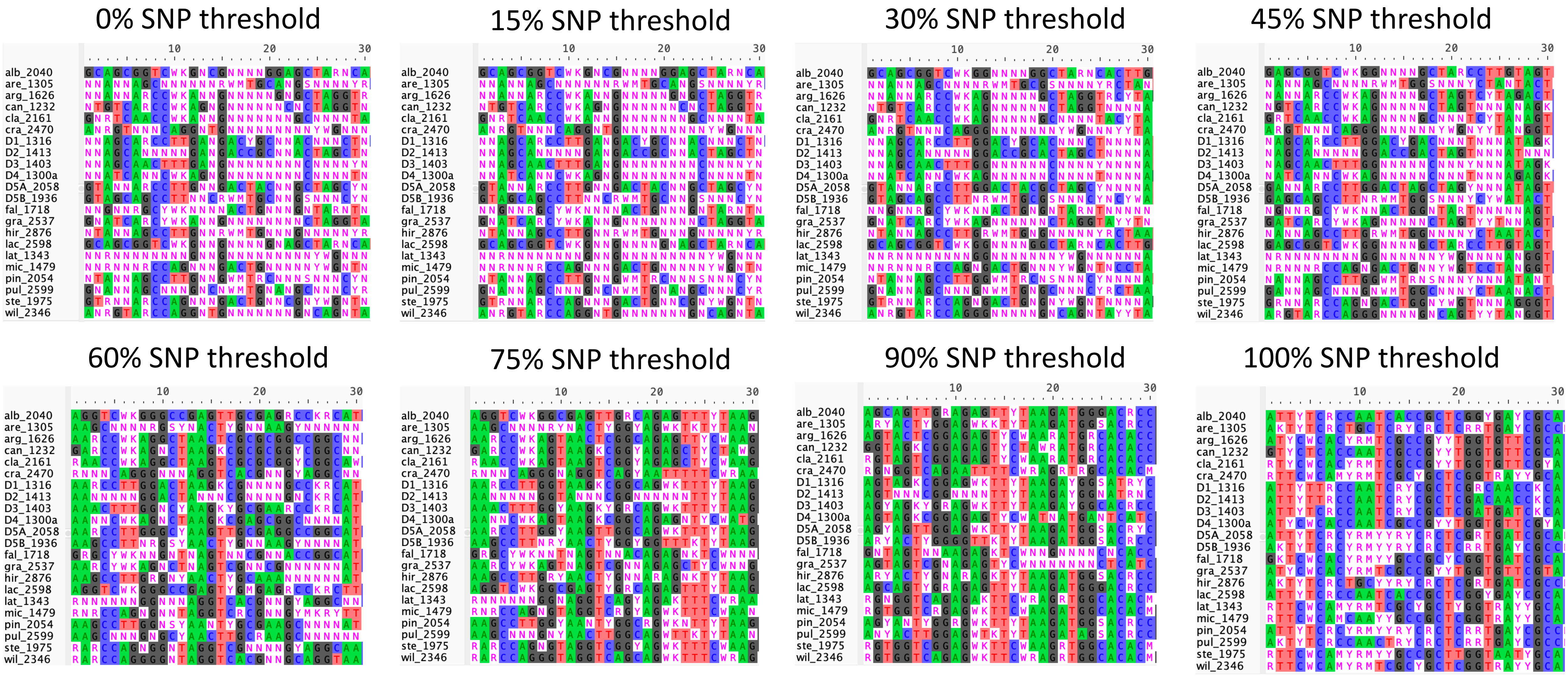

DOI 10.1002/aps3.11611

Figure S2. Depiction of the three unique topologies inferred across the 16 different datasets analyzed with RAxML. (A, B) The two most frequently inferred trees, with incongruence between the two found in the H-genome group. (C) The topology inferred only with the most strict SNP-filtering scheme where all SNPs were required to be found in all 22 individuals (100% SNP filtering).

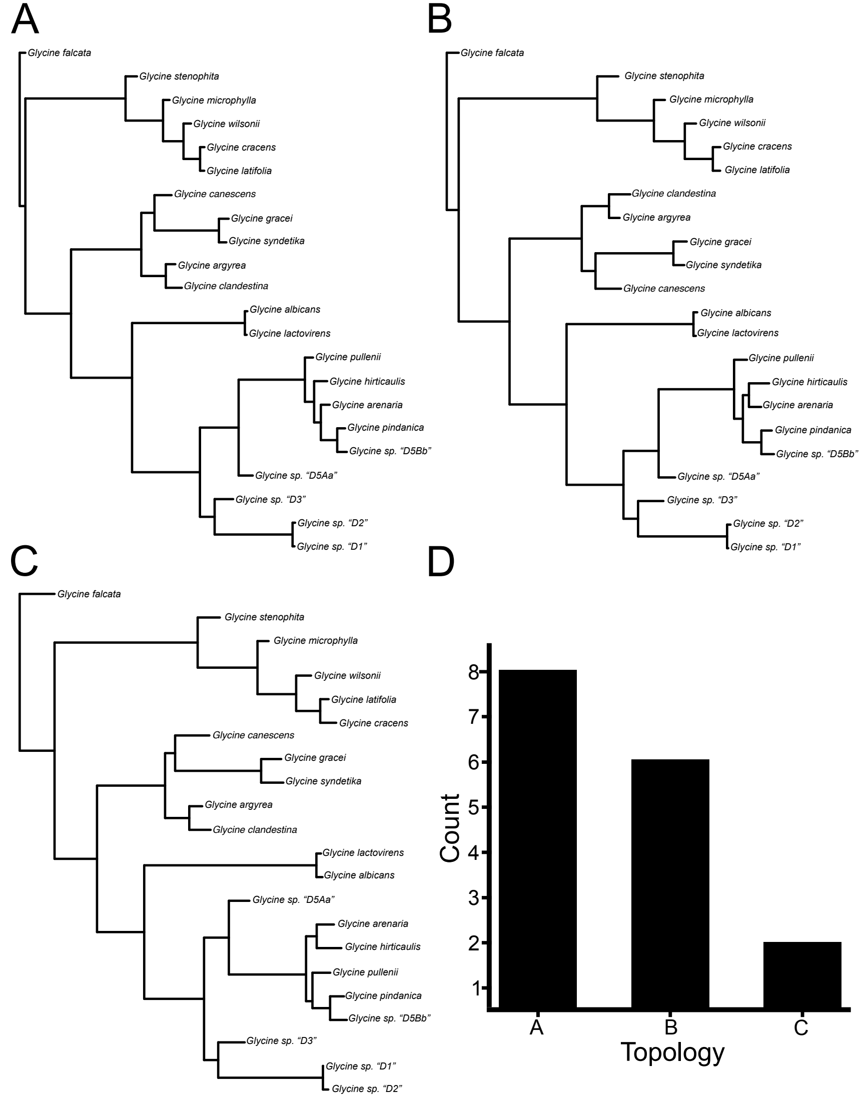

DOI 10.1002/aps3.11611

Figure S3. Node support comparisons across 28 trees inferred from downsampled alignments of the different thresholds compared to the 100% threshold tree.

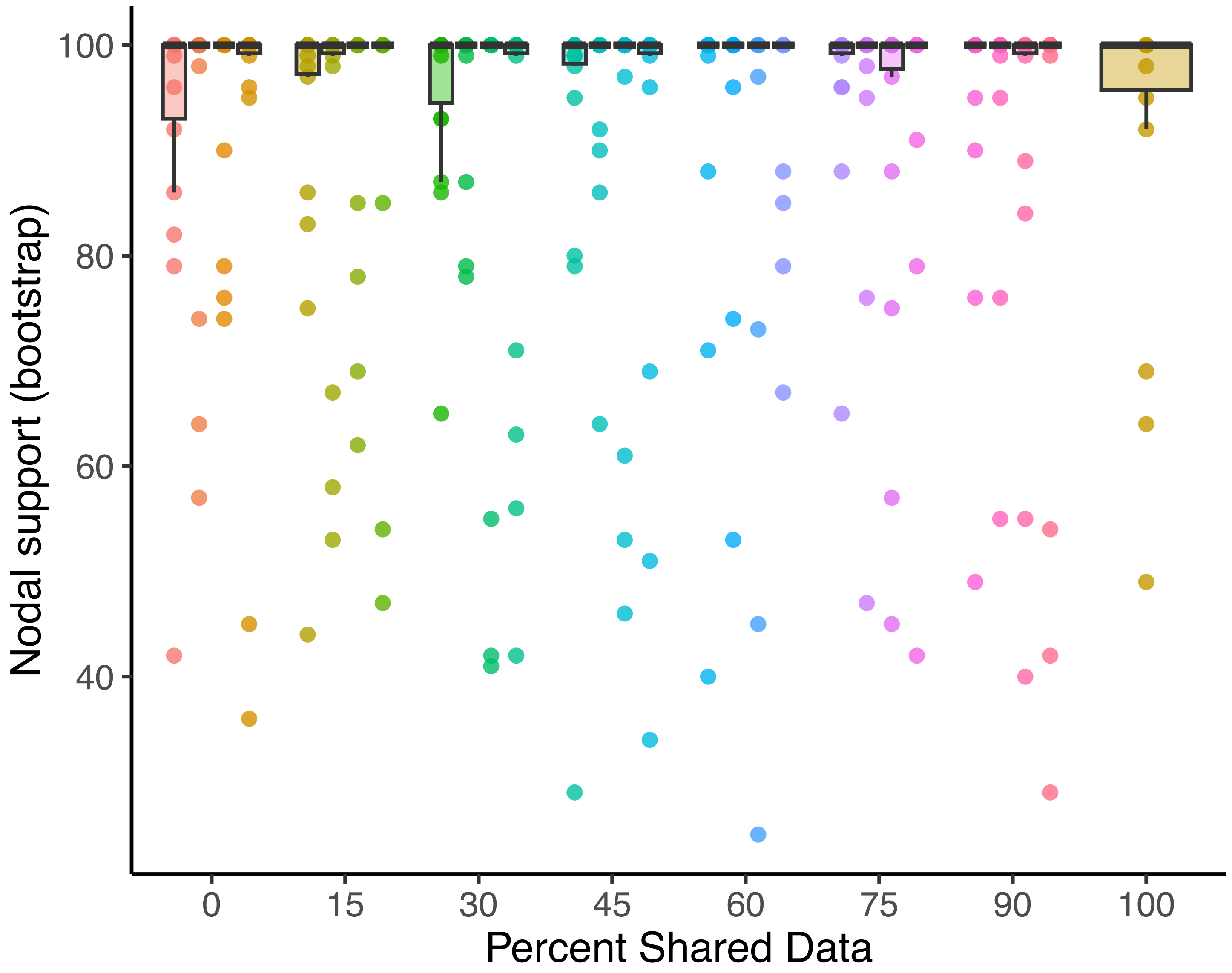

DOI 10.1002/aps3.11611

Figure S4. Branch length and node support comparisons across 16 trees inferred from simulated GBS data. (A) Comparison of branch lengths between the SNP and locus datasets by filtering stringency. (B) Comparison of nodal support between the SNP and locus datasets by filtering stringency.

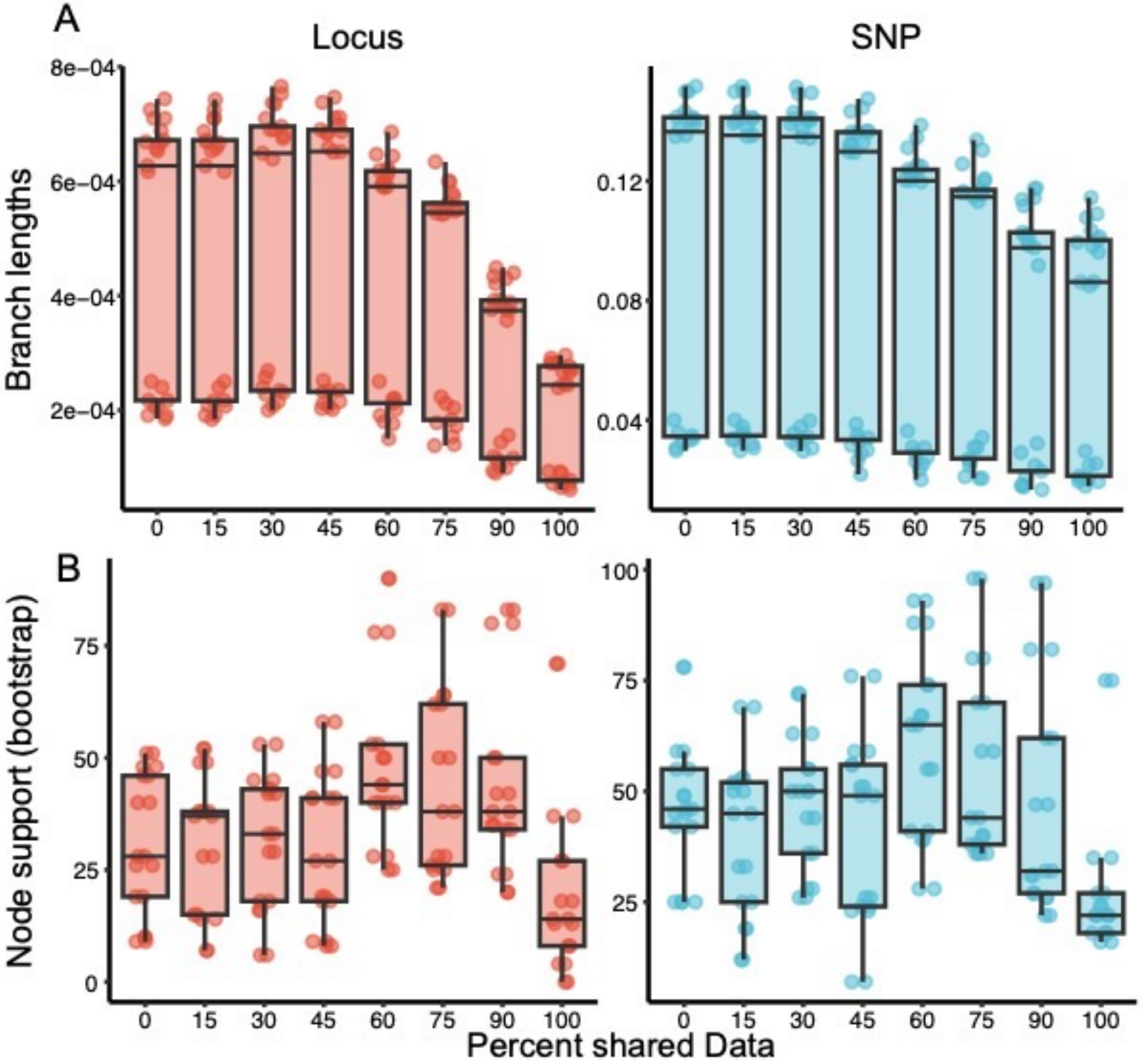

Supplement: Supplementary file 1 — Appendix S1. Supplemental figures for “Data‐driven guidelines for phylogenomic analyses using SNP data.” [file APS3-12-e11611-s003.pdf]
