# Supplementary material for: Data‐driven guidelines for phylogenomic analyses using SNP data
Source: Appl Plant Sci. 2024 Aug 9;12(6):e11611. doi: 10.1002/aps3.11611 (PMC11610416; doi:10.1002/aps3.11611)

DOI 10.1002/aps3.11611

Appendix S3. Inferred topologies from all empirical datasets

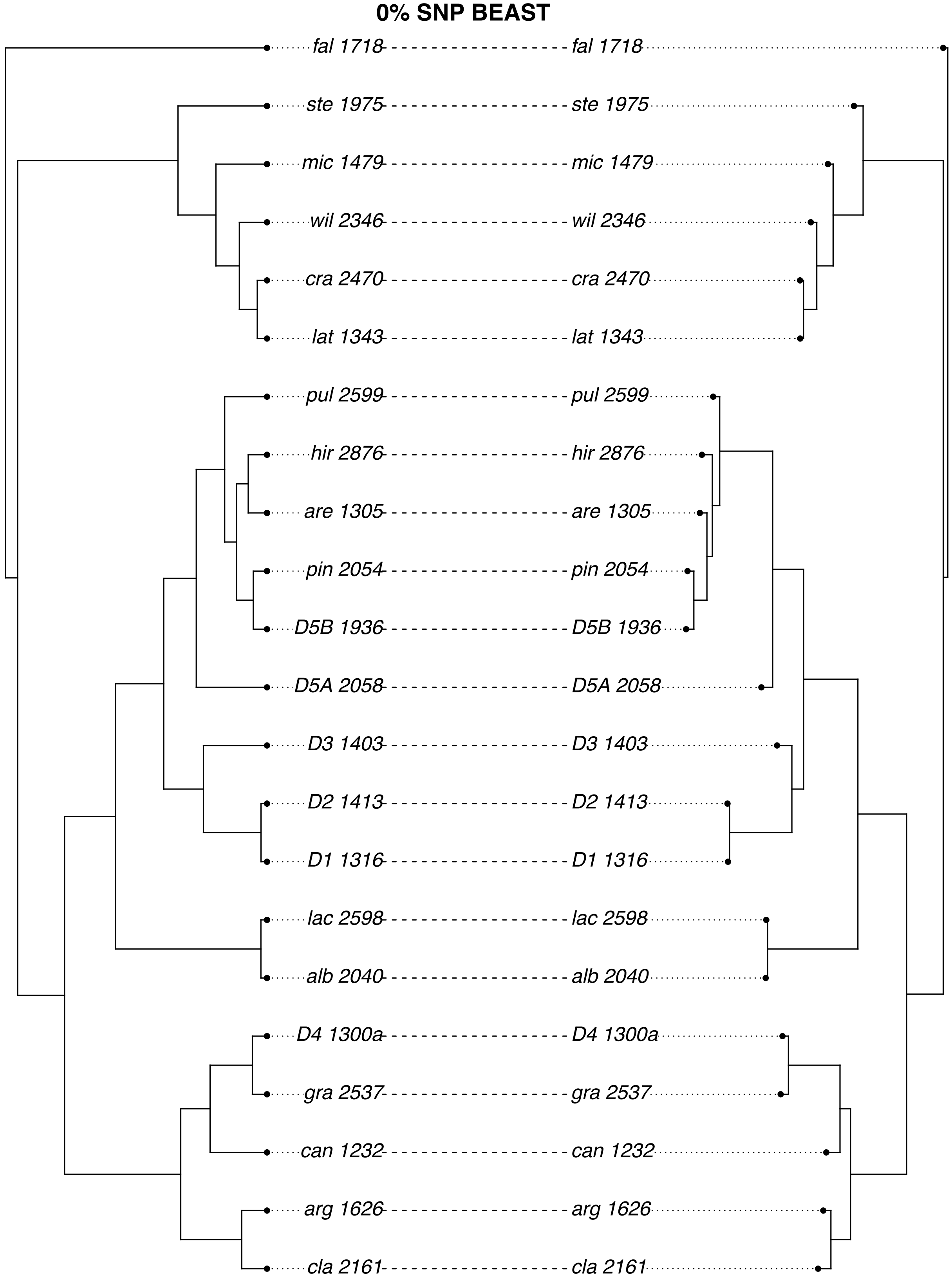

DOI 10.1002/aps3.11611
Appendix S3. Inferred topologies from all empirical datasets

15% SNP BEAST

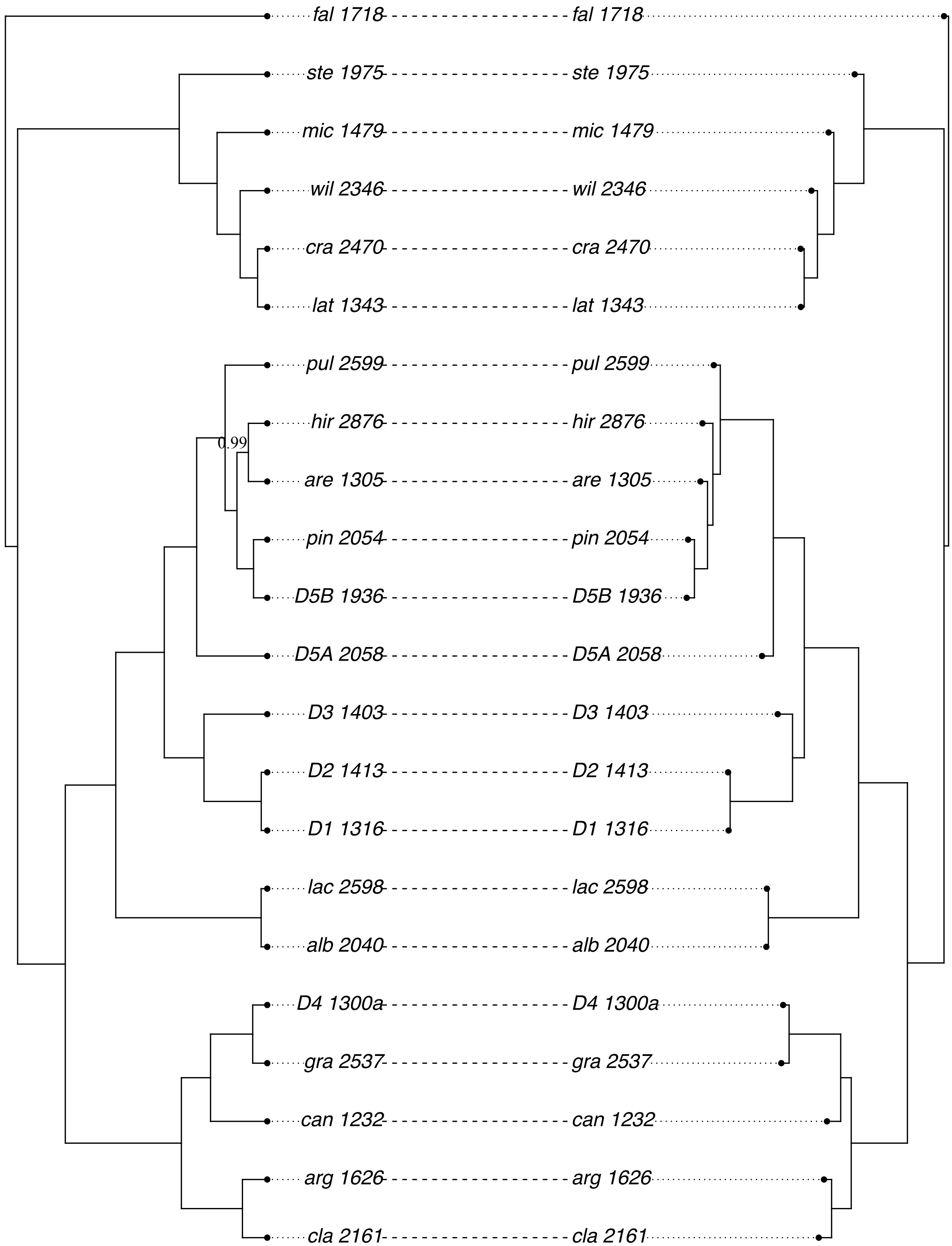

DOI 10.1002/aps3.11611
Appendix S3. Inferred topologies from all empirical datasets

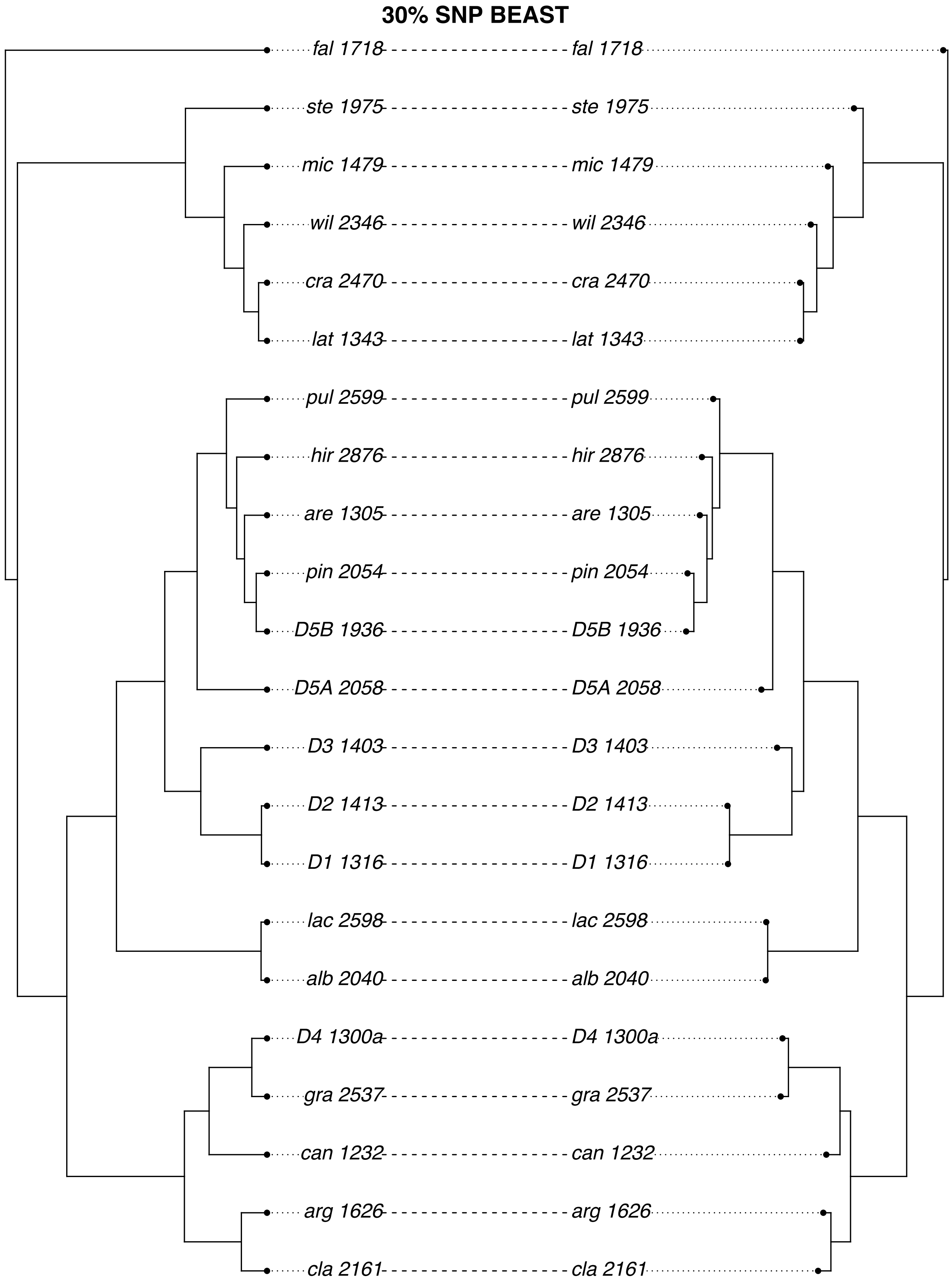

DOI 10.1002/aps3.11611
Appendix S3. Inferred topologies from all empirical datasets

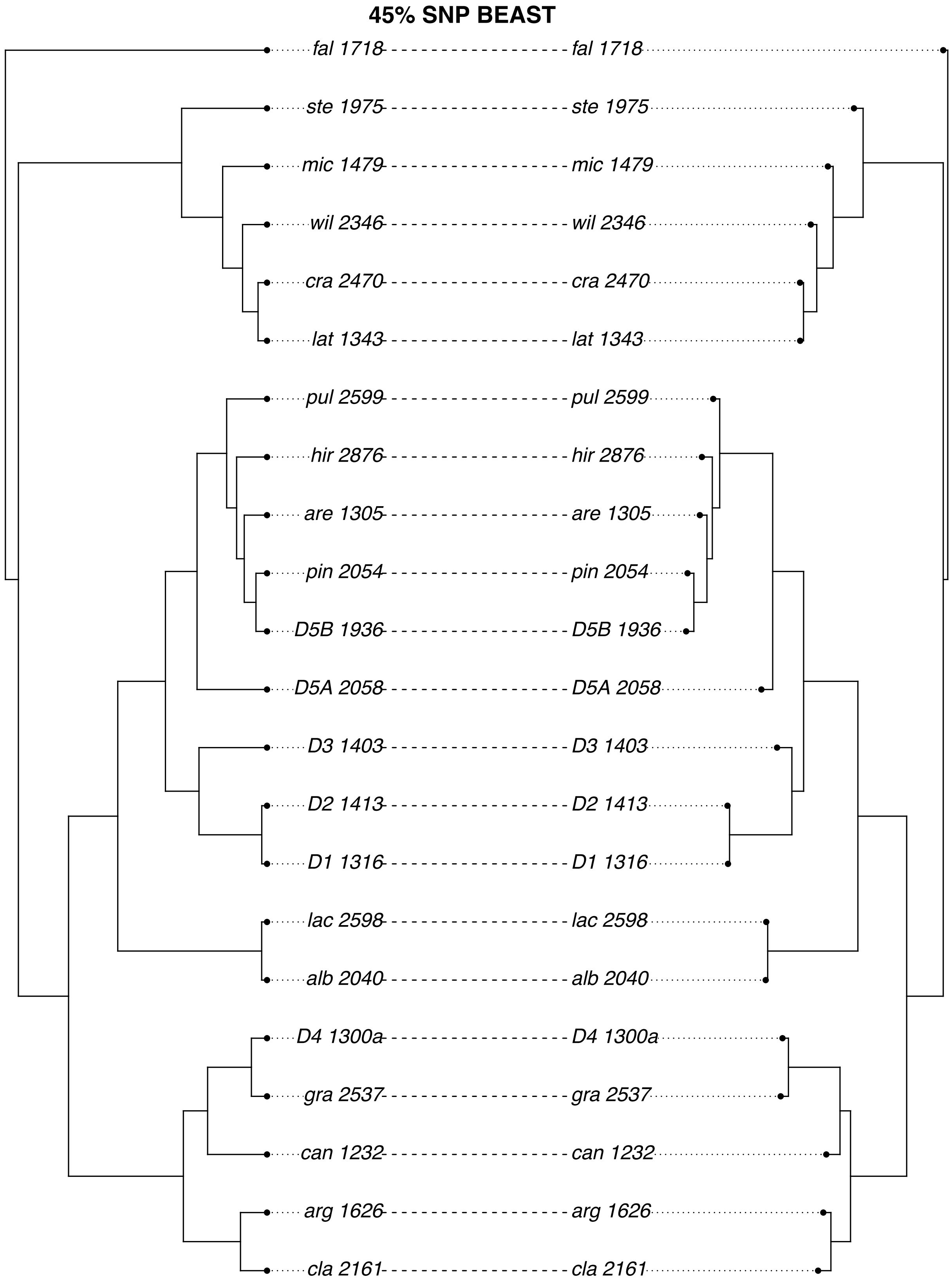

DOI 10.1002/aps3.11611
Appendix S3. Inferred topologies from all empirical datasets

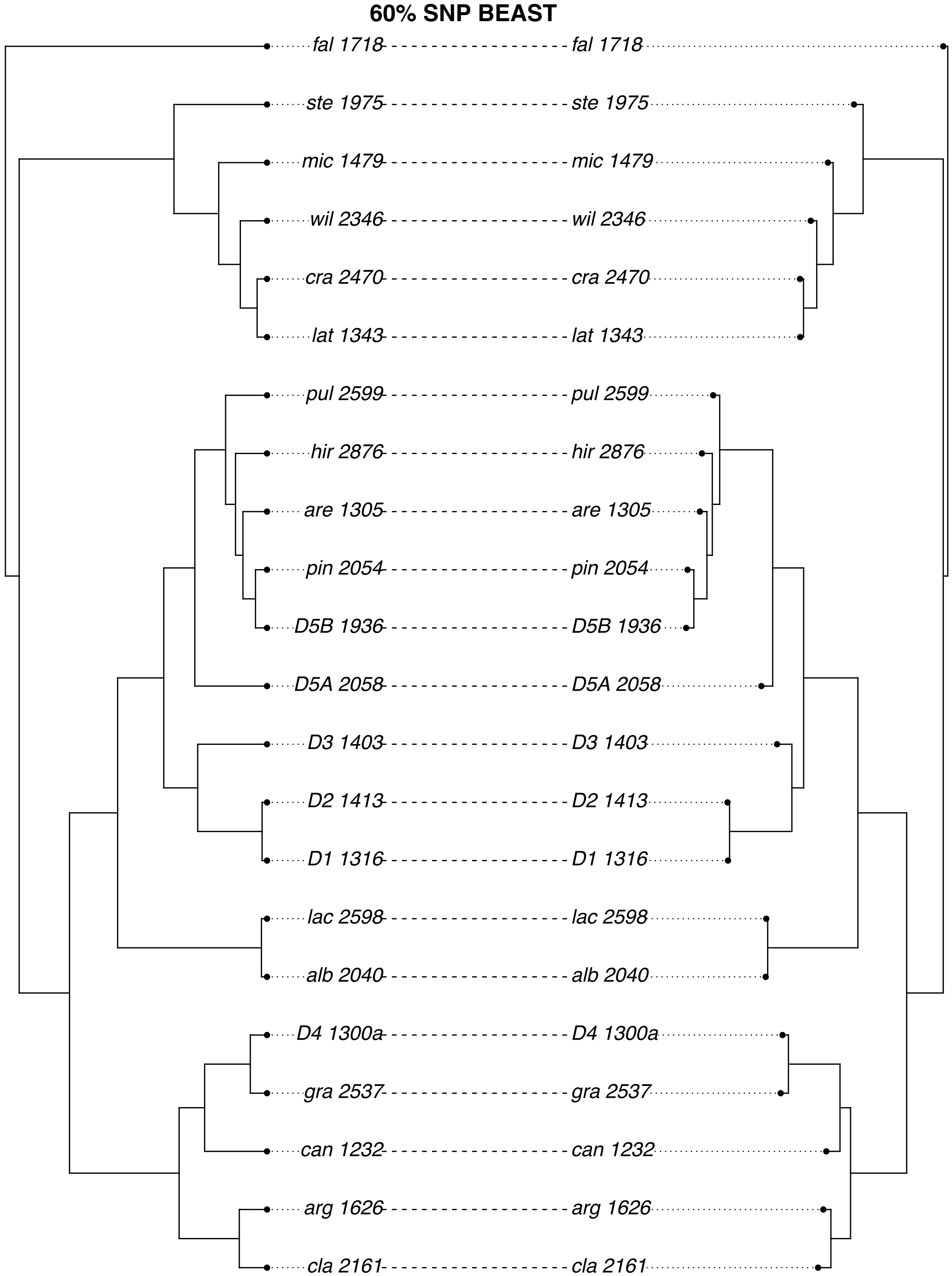

DOI 10.1002/aps3.11611
Appendix S3. Inferred topologies from all empirical datasets

75% SNP BEAST

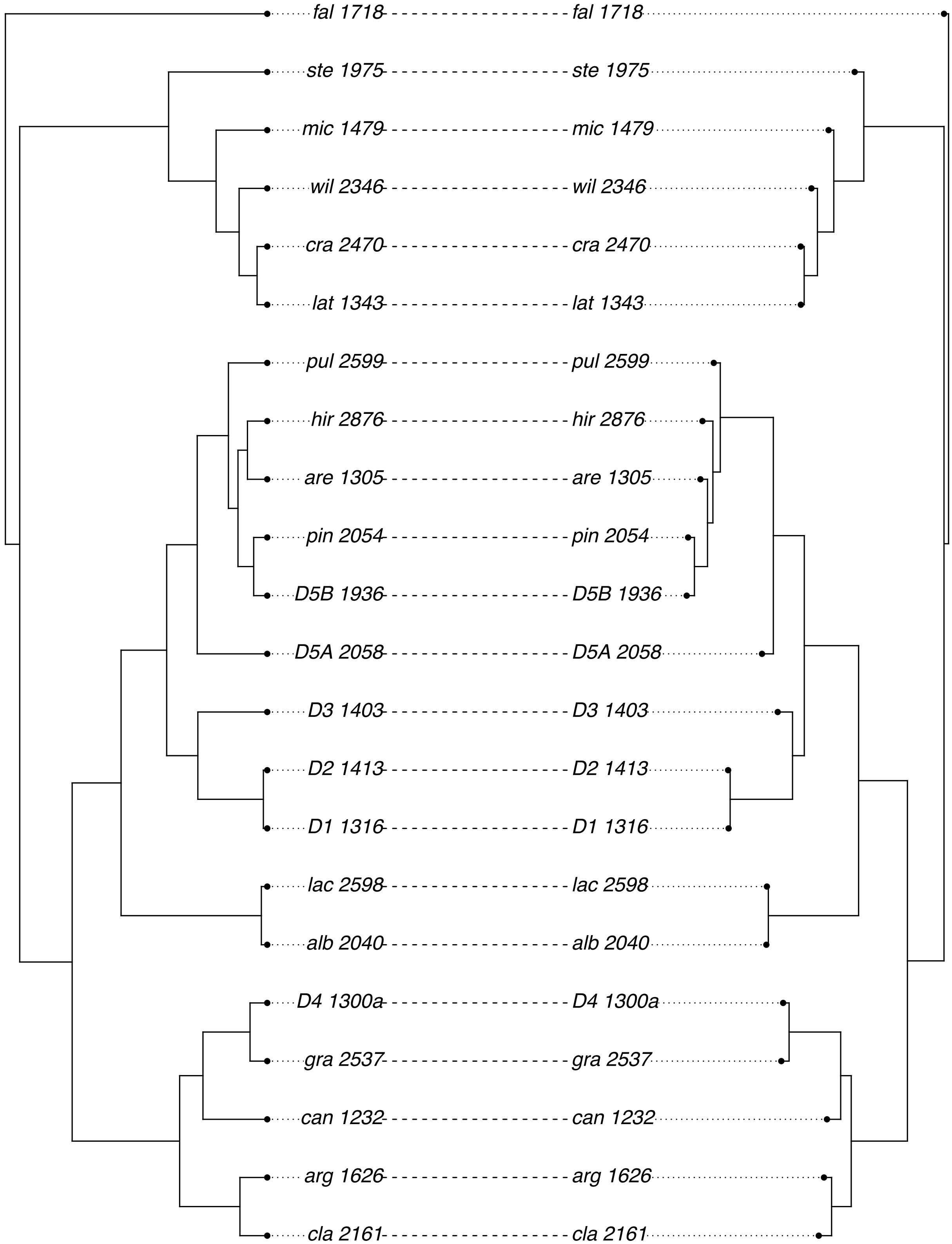

DOI 10.1002/aps3.11611
Appendix S3. Inferred topologies from all empirical datasets

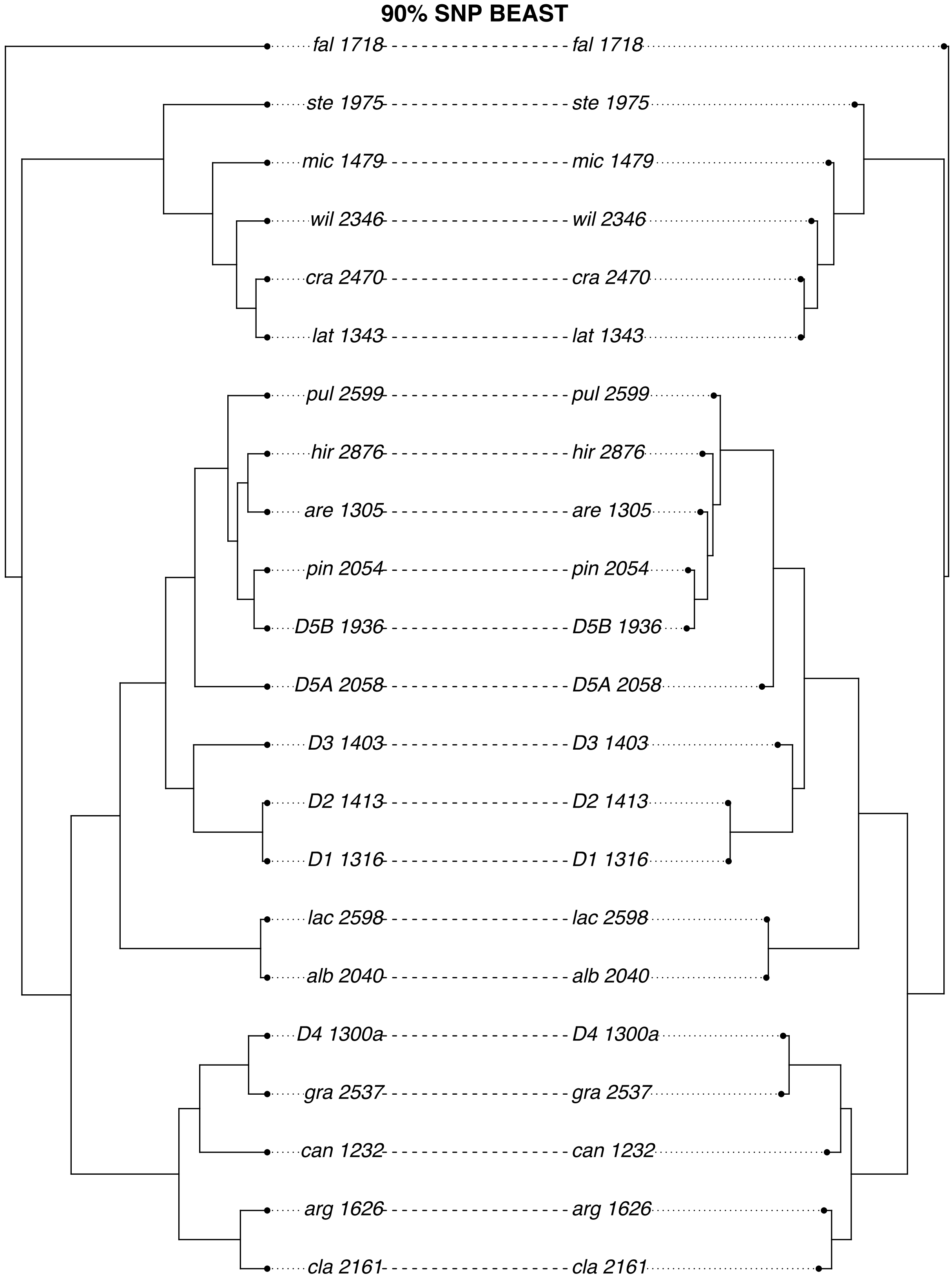

DOI 10.1002/aps3.11611
Appendix S3. Inferred topologies from all empirical datasets

100% SNP BEAST

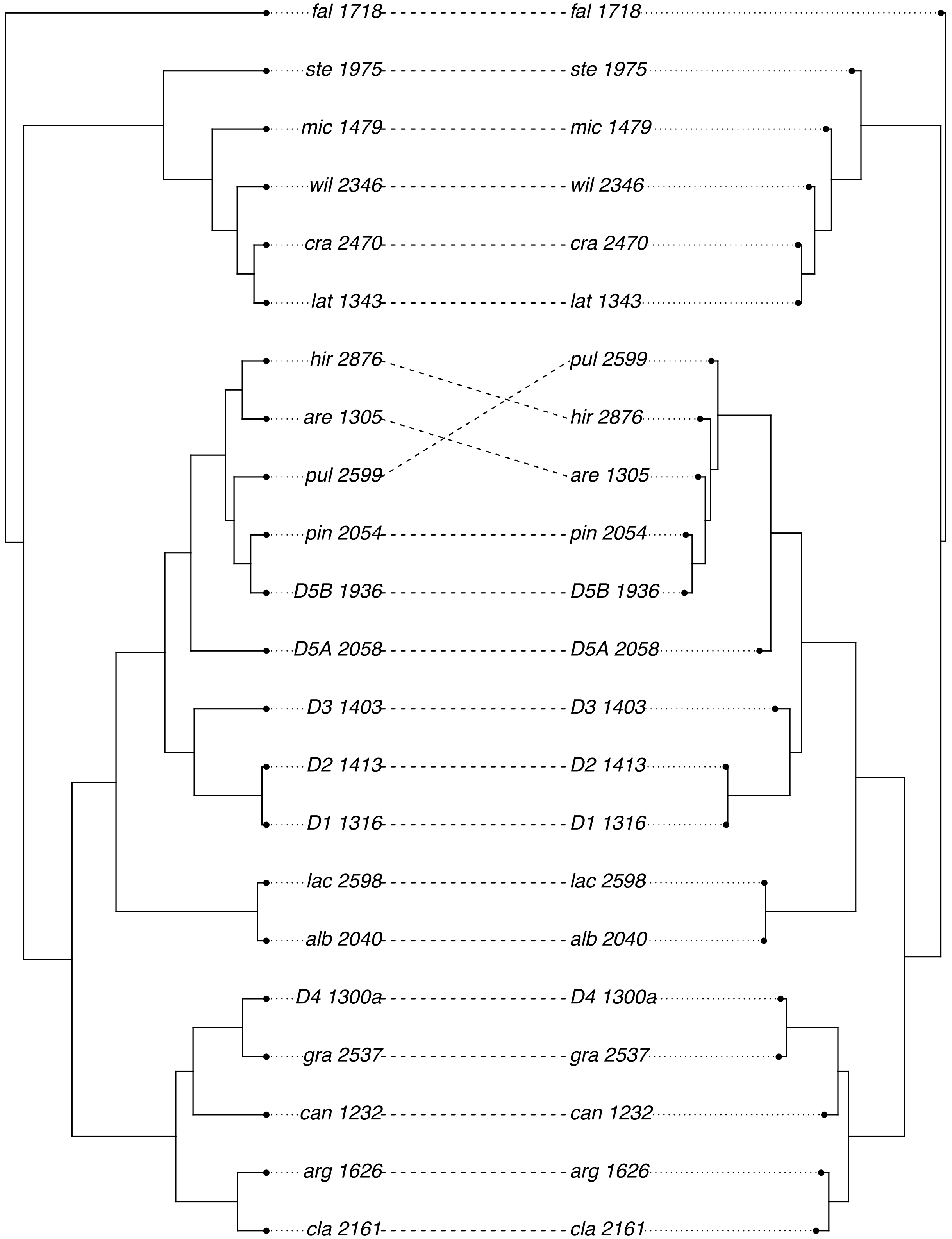

DOI 10.1002/aps3.11611
Appendix S3. Inferred topologies from all empirical datasets

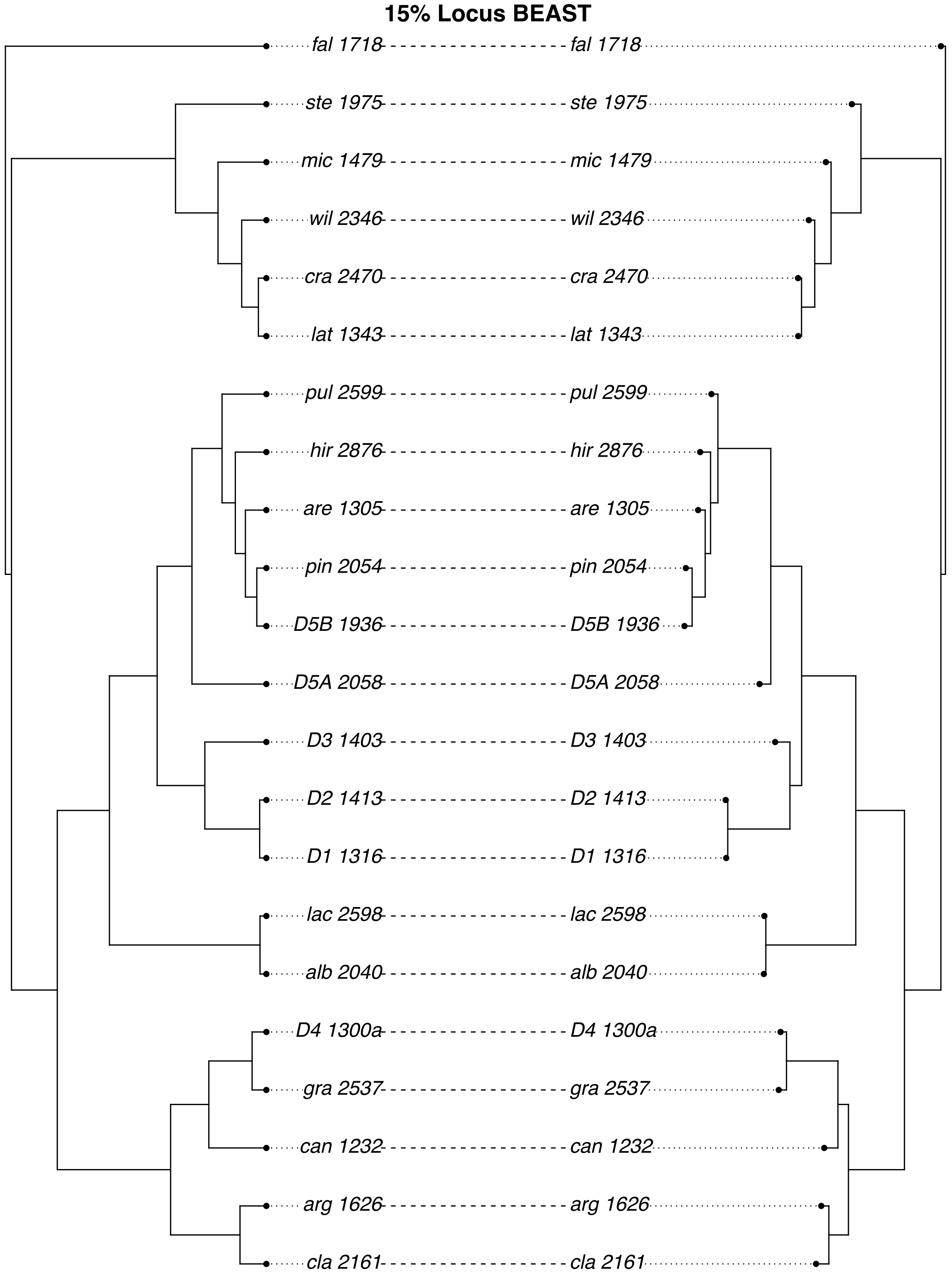

DOI 10.1002/aps3.11611
Appendix S3. Inferred topologies from all empirical datasets

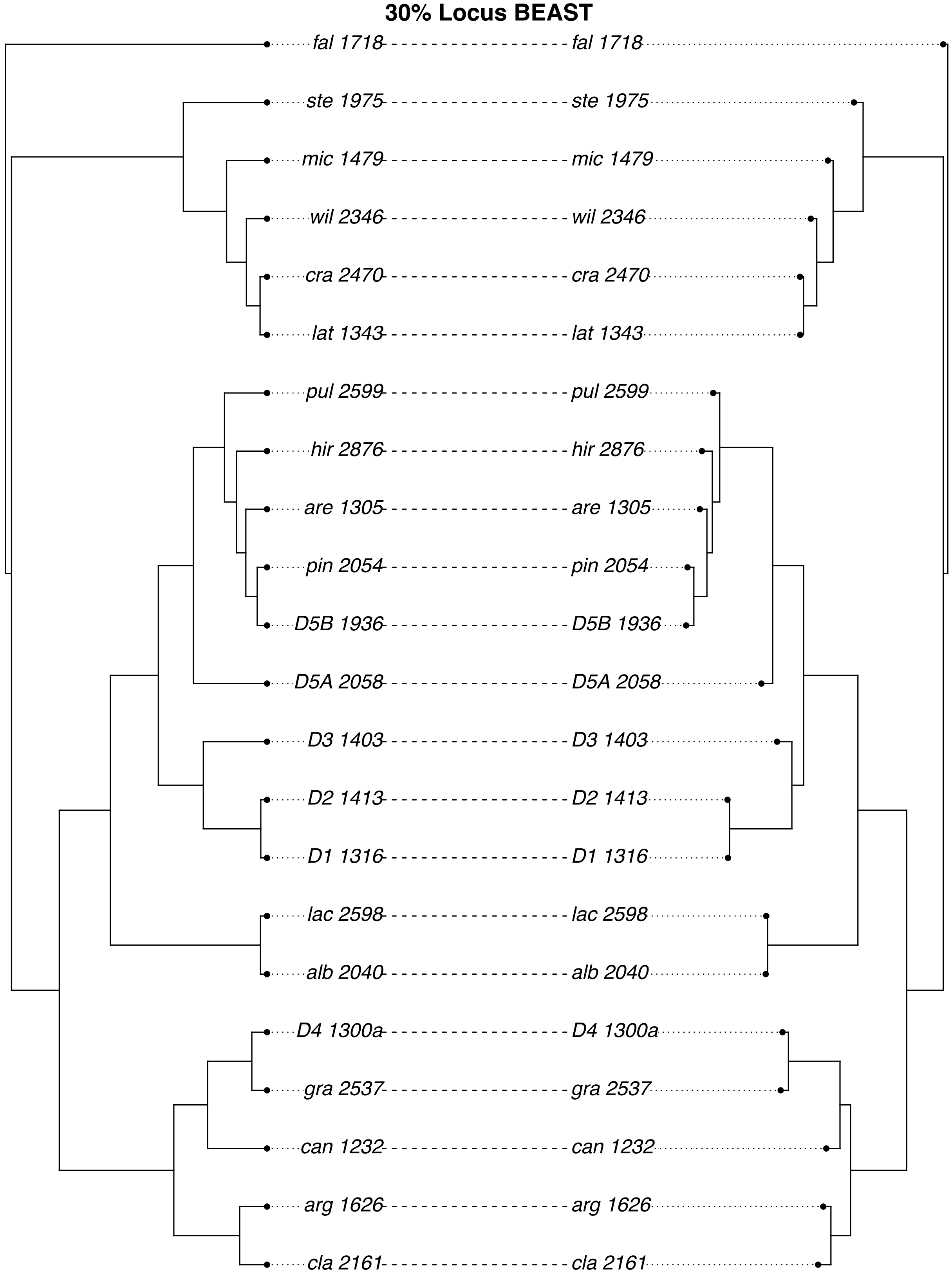

DOI 10.1002/aps3.11611
Appendix S3. Inferred topologies from all empirical datasets

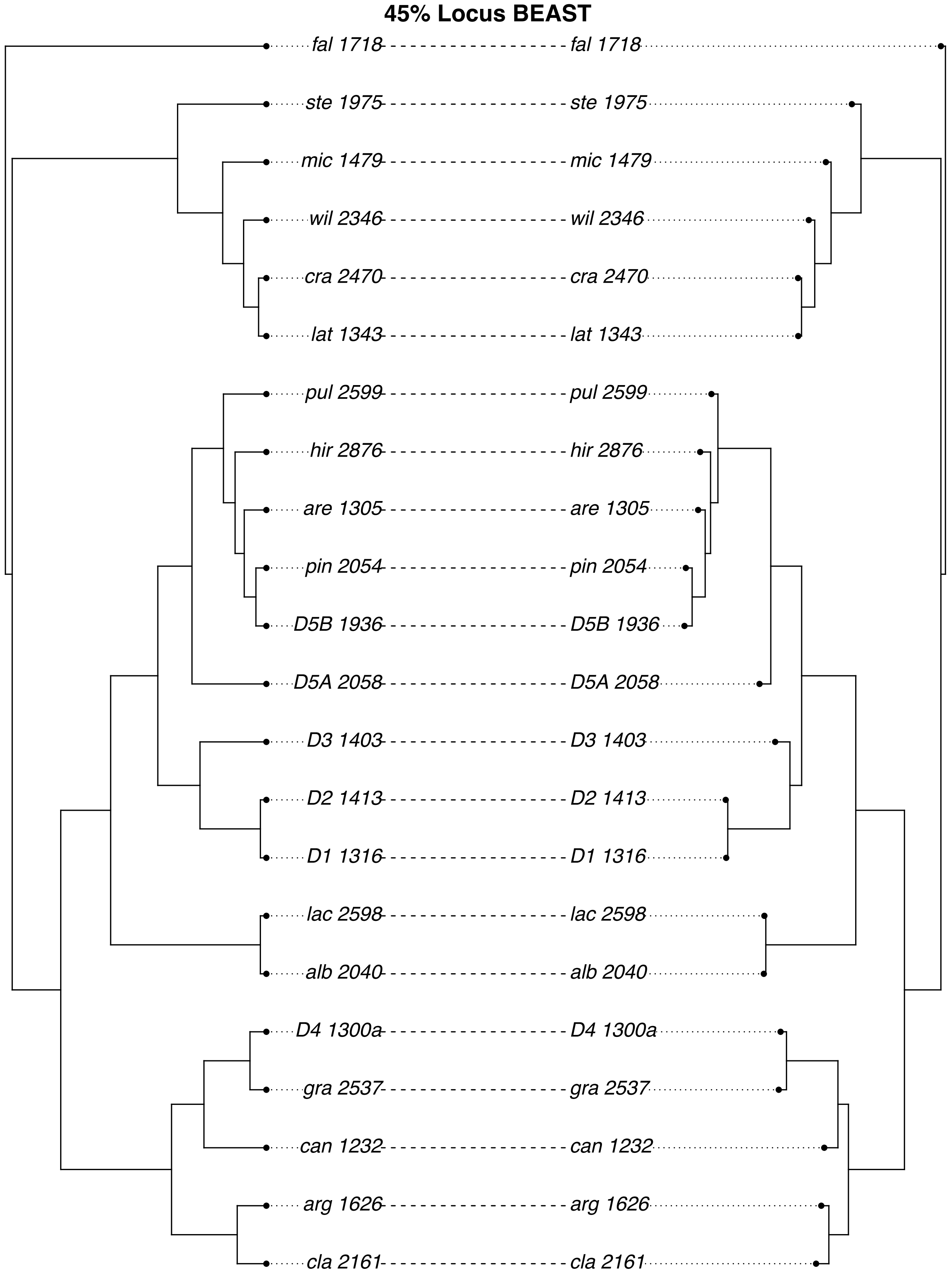

DOI 10.1002/aps3.11611
Appendix S3. Inferred topologies from all empirical datasets

60% Locus BEAST

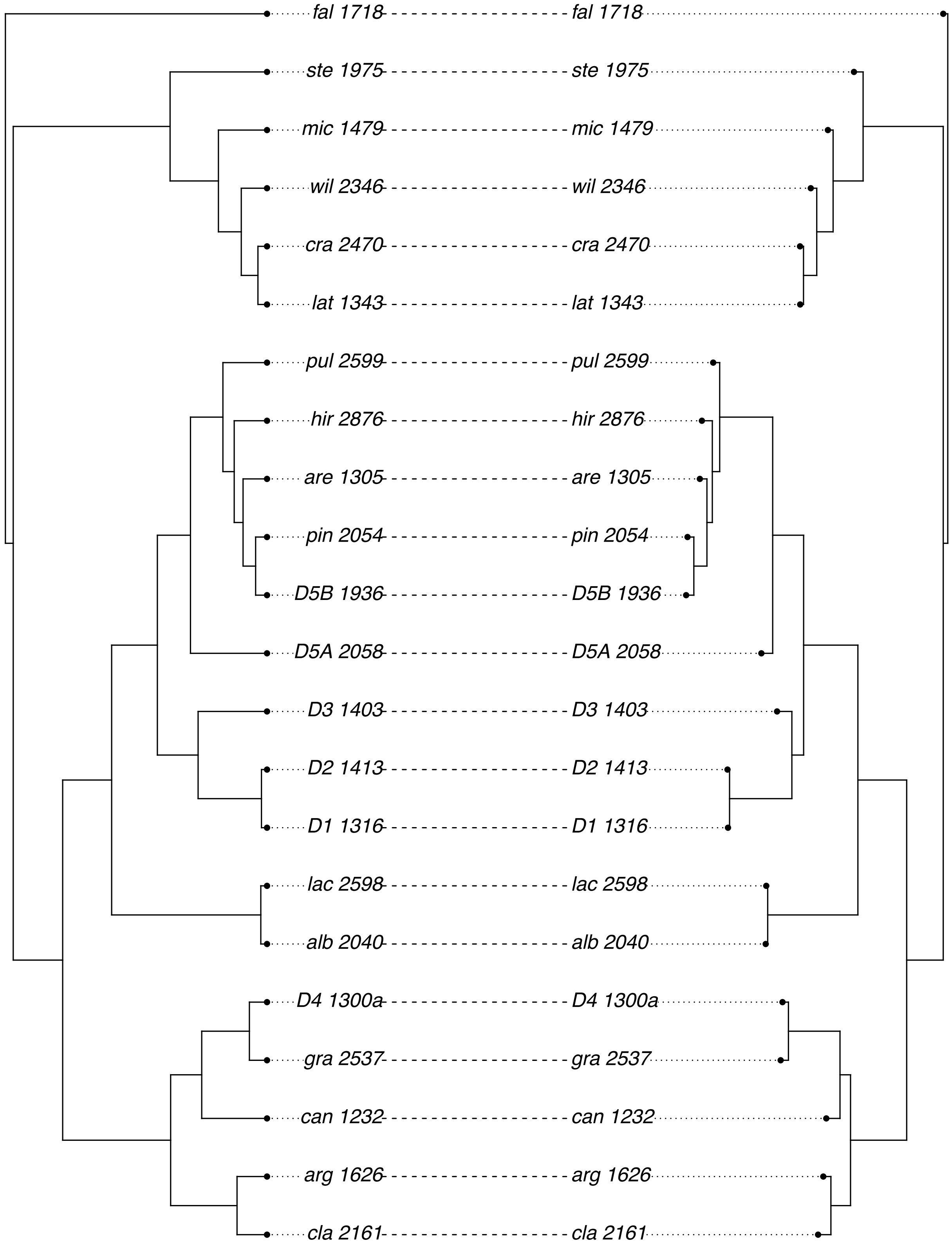

DOI 10.1002/aps3.11611
Appendix S3. Inferred topologies from all empirical datasets

75% Locus BEAST

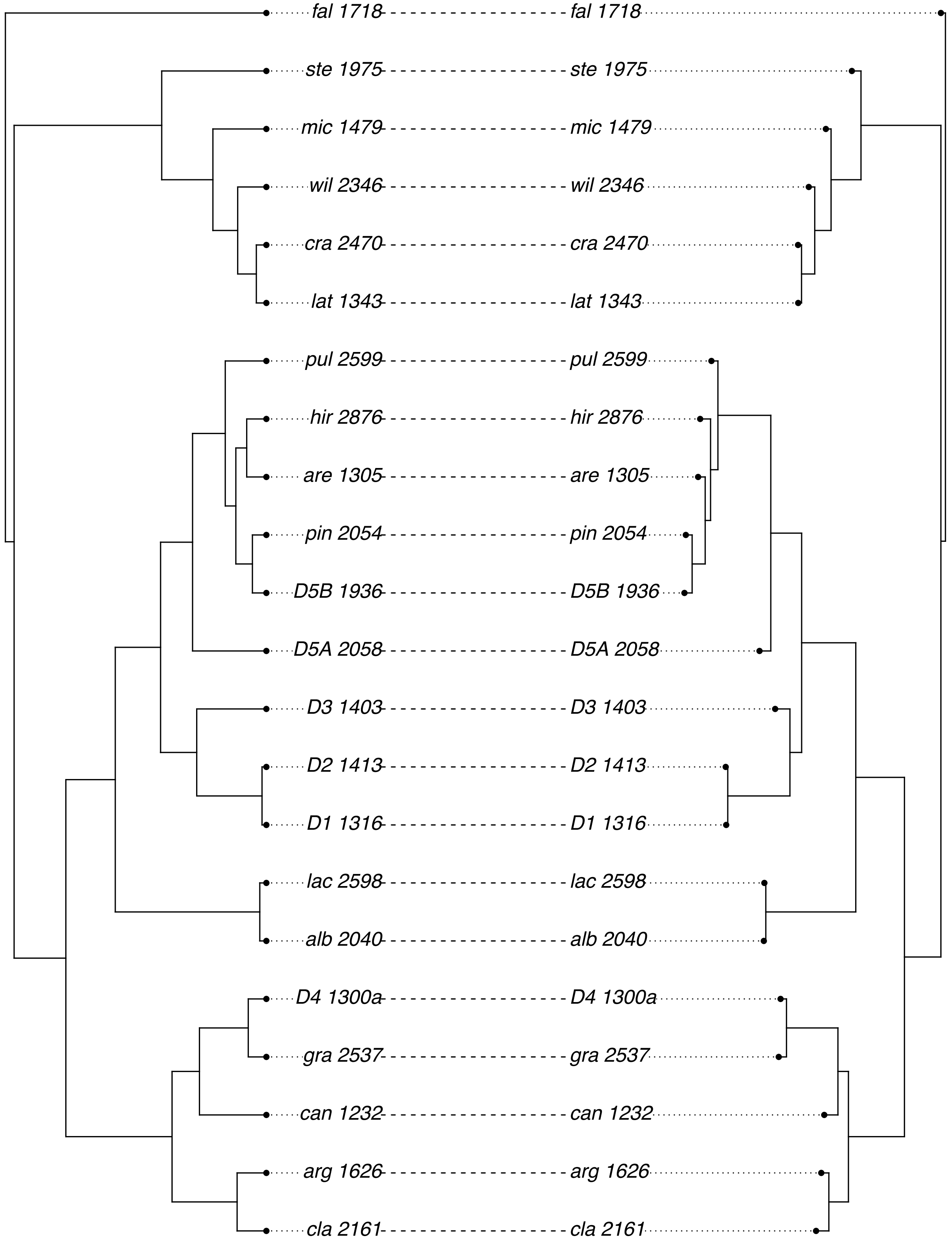

DOI 10.1002/aps3.11611
Appendix S3. Inferred topologies from all empirical datasets

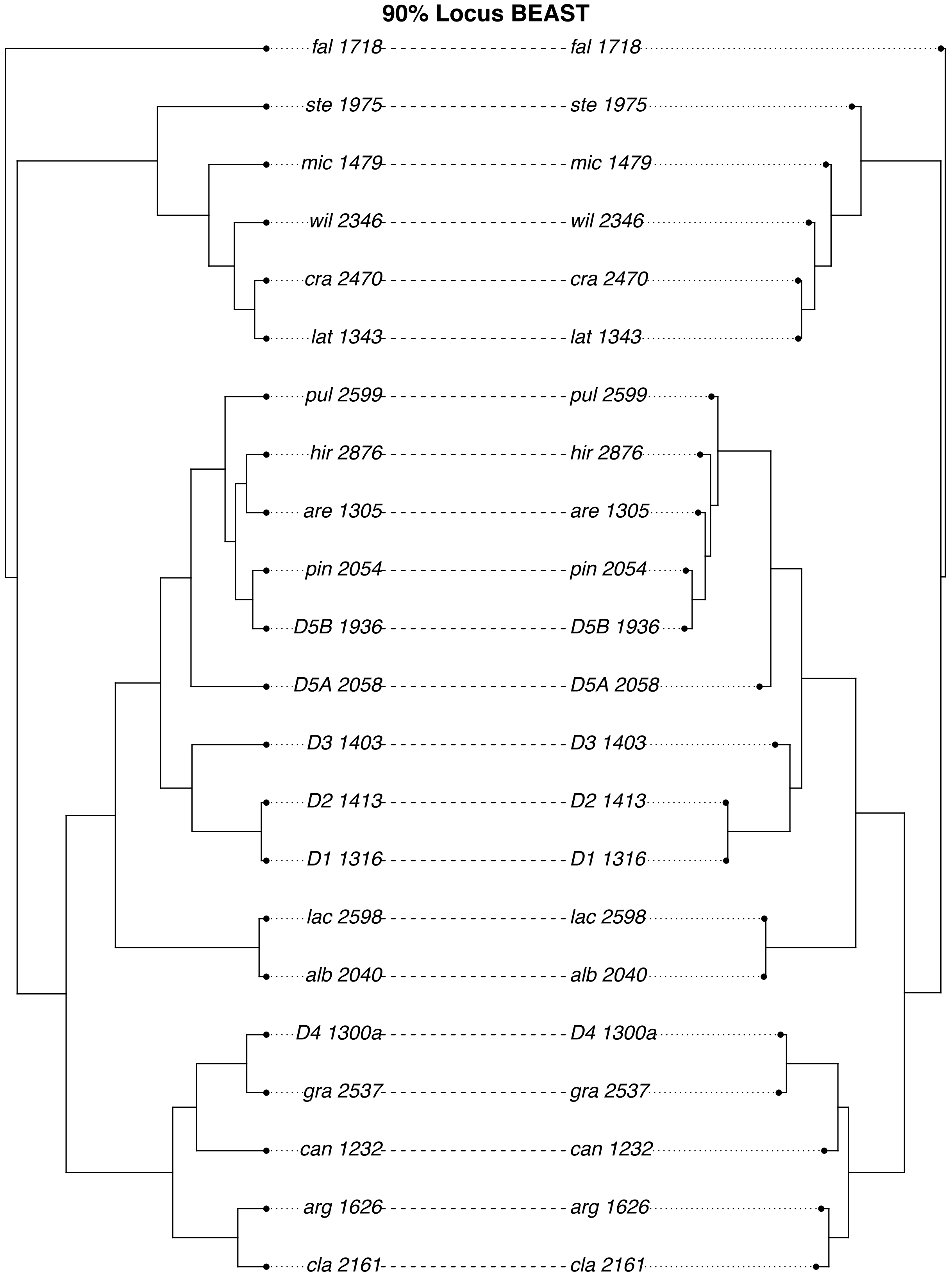

DOI 10.1002/aps3.11611
Appendix S3. Inferred topologies from all empirical datasets

100% Locus BEAST

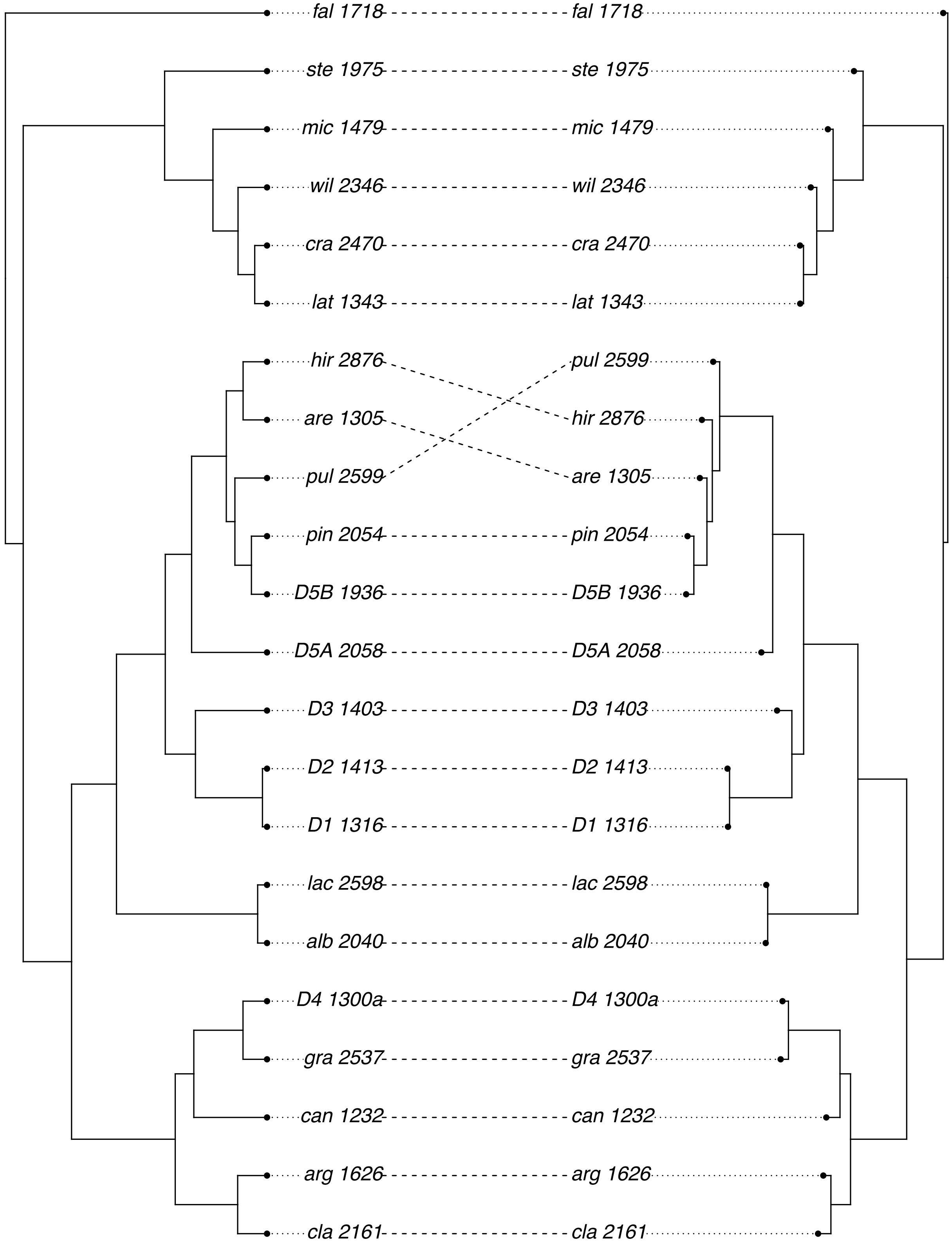

DOI 10.1002/aps3.11611
Appendix S3. Inferred topologies from all empirical datasets

0% SNP RAxML

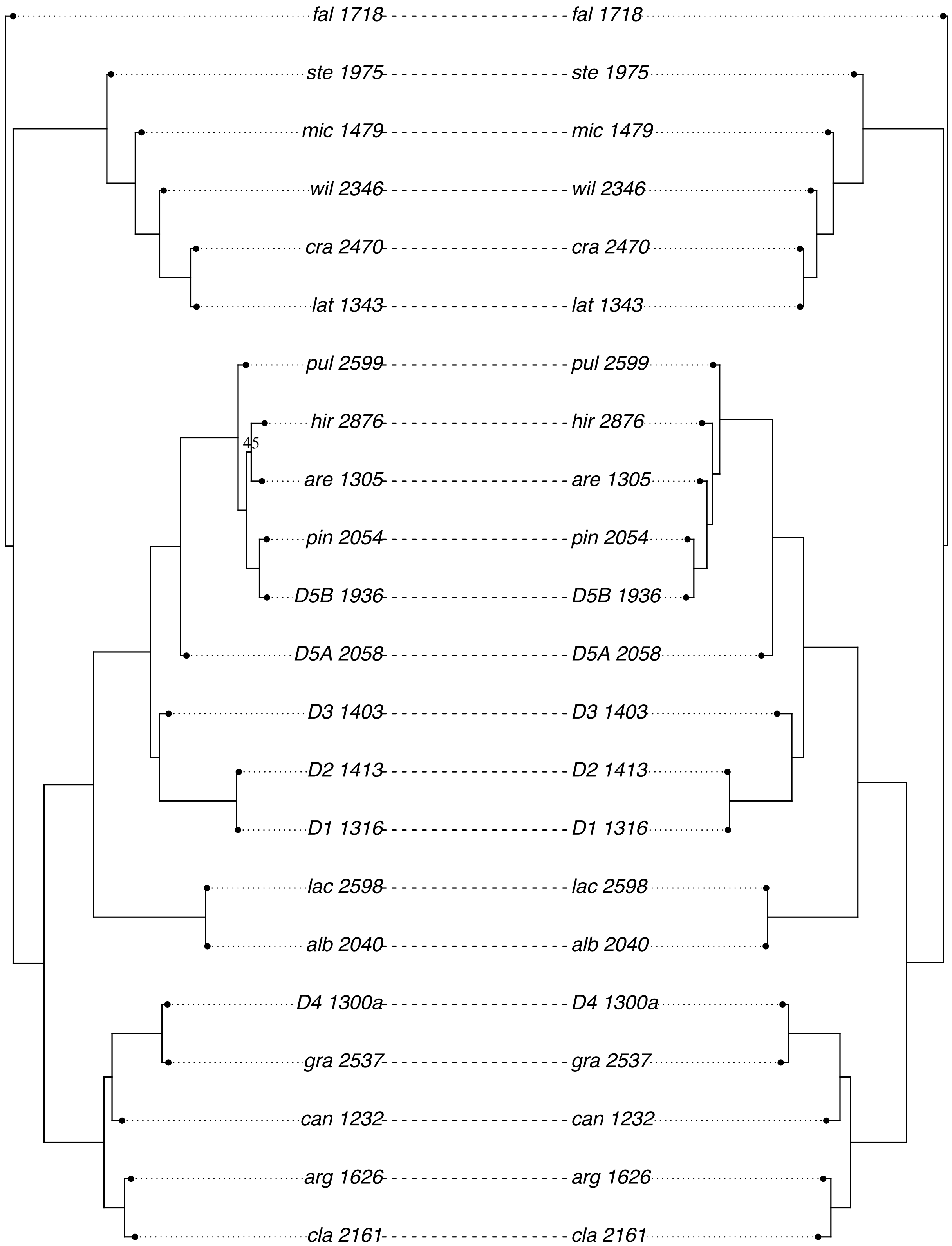

DOI 10.1002/aps3.11611
Appendix S3. Inferred topologies from all empirical datasets

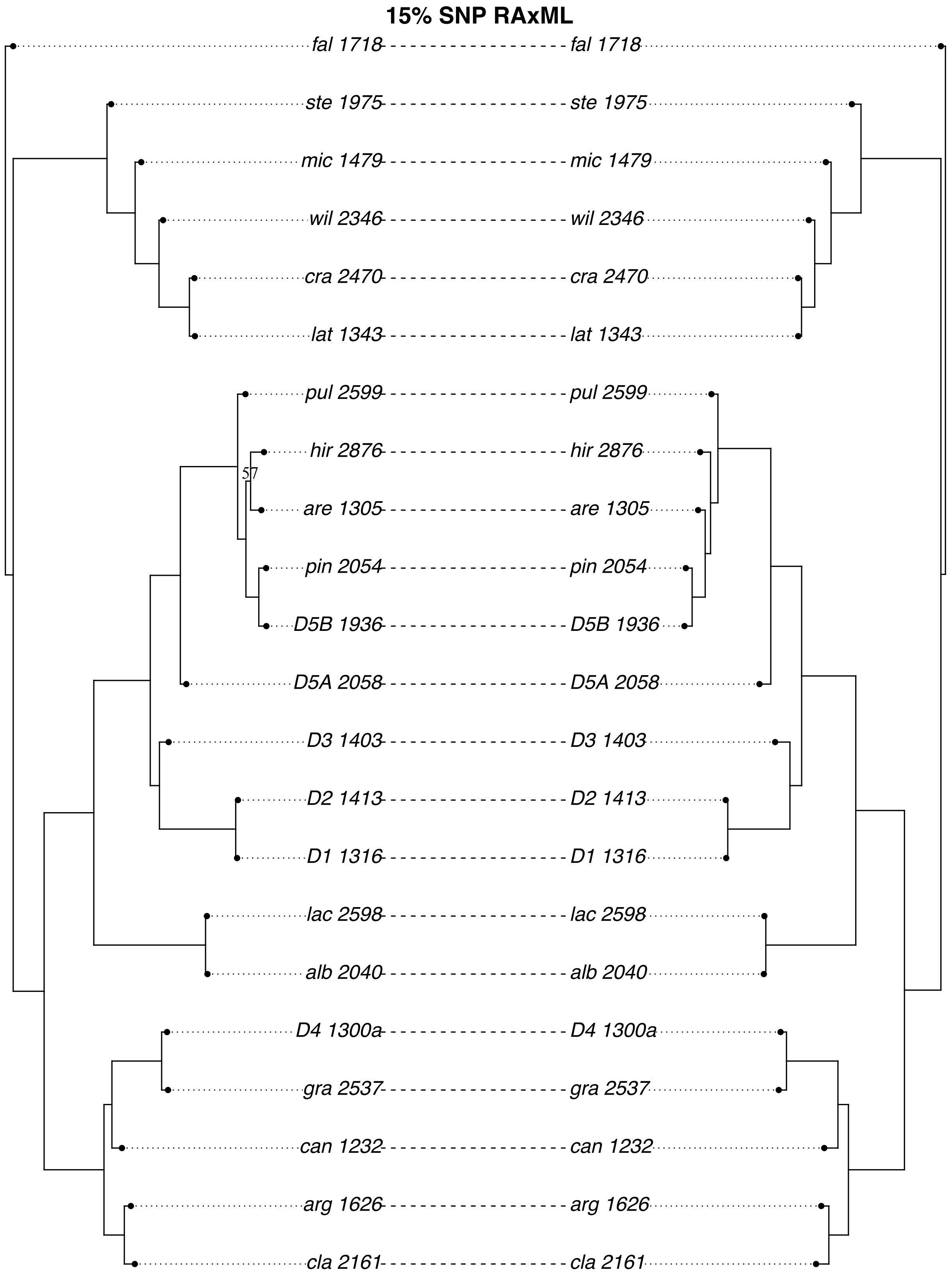

DOI 10.1002/aps3.11611
Appendix S3. Inferred topologies from all empirical datasets

30% SNP RAxML

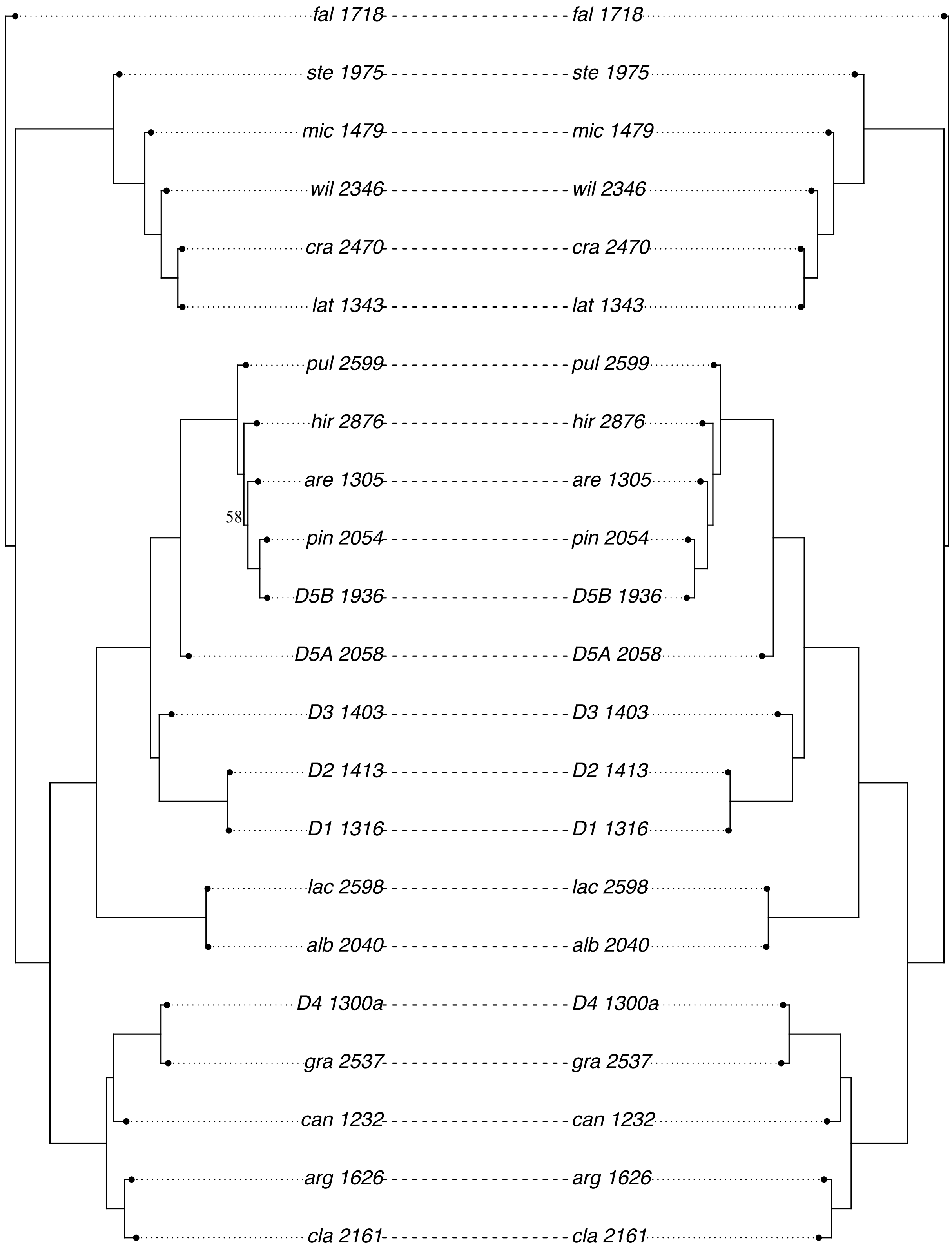

DOI 10.1002/aps3.11611
Appendix S3. Inferred topologies from all empirical datasets

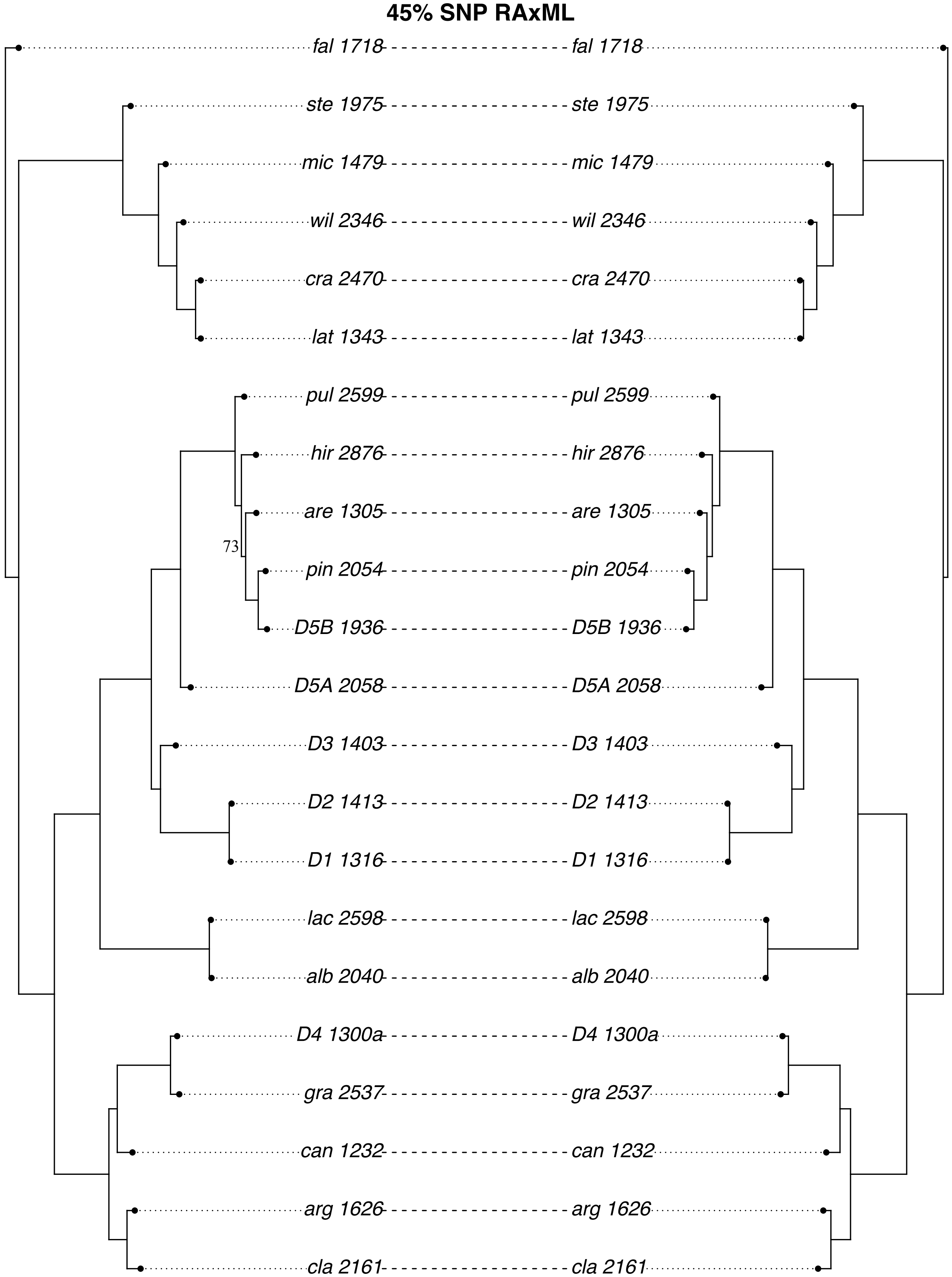

DOI 10.1002/aps3.11611
Appendix S3. Inferred topologies from all empirical datasets

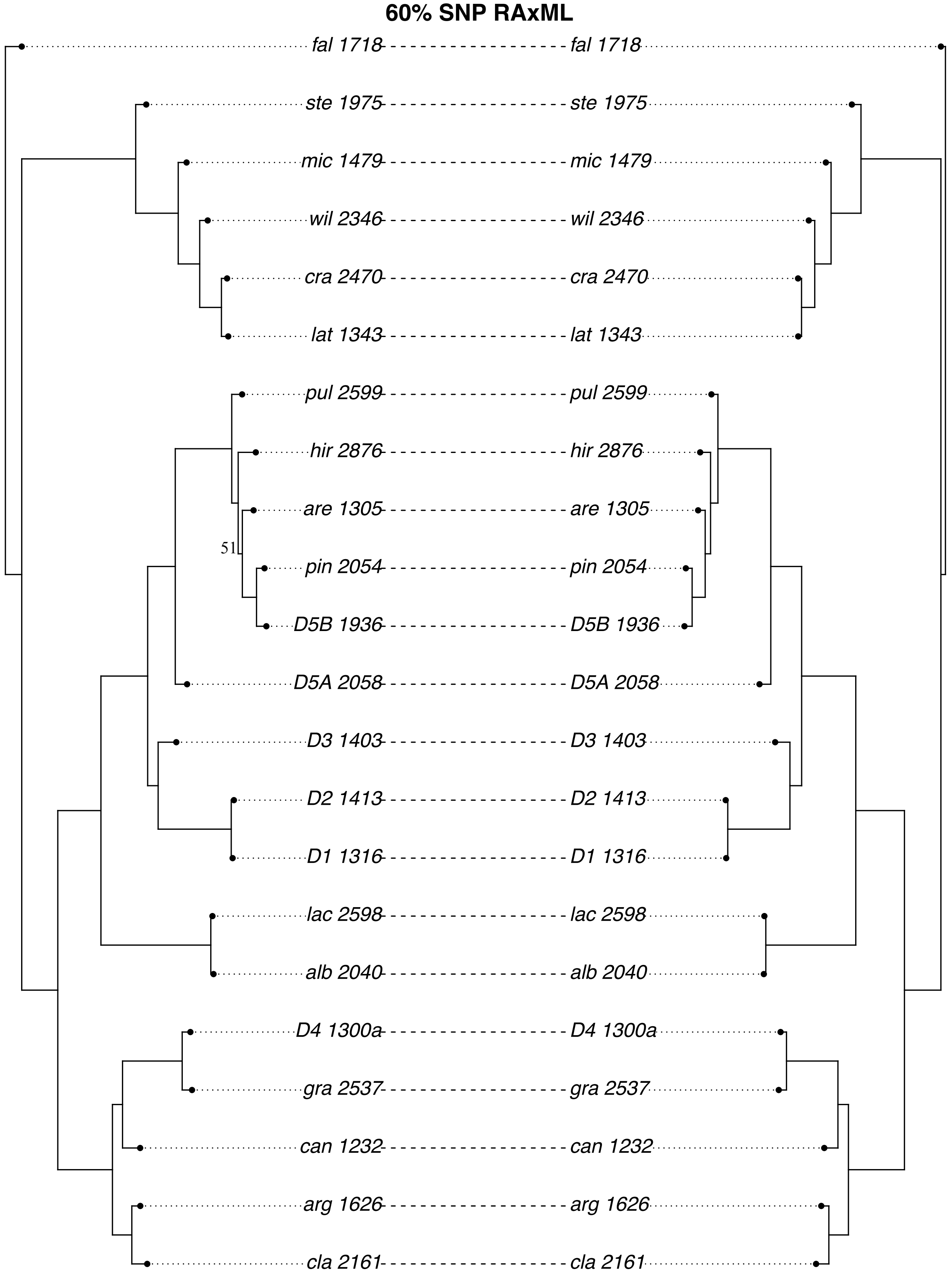

DOI 10.1002/aps3.11611
Appendix S3. Inferred topologies from all empirical datasets

75% SNP RAxML

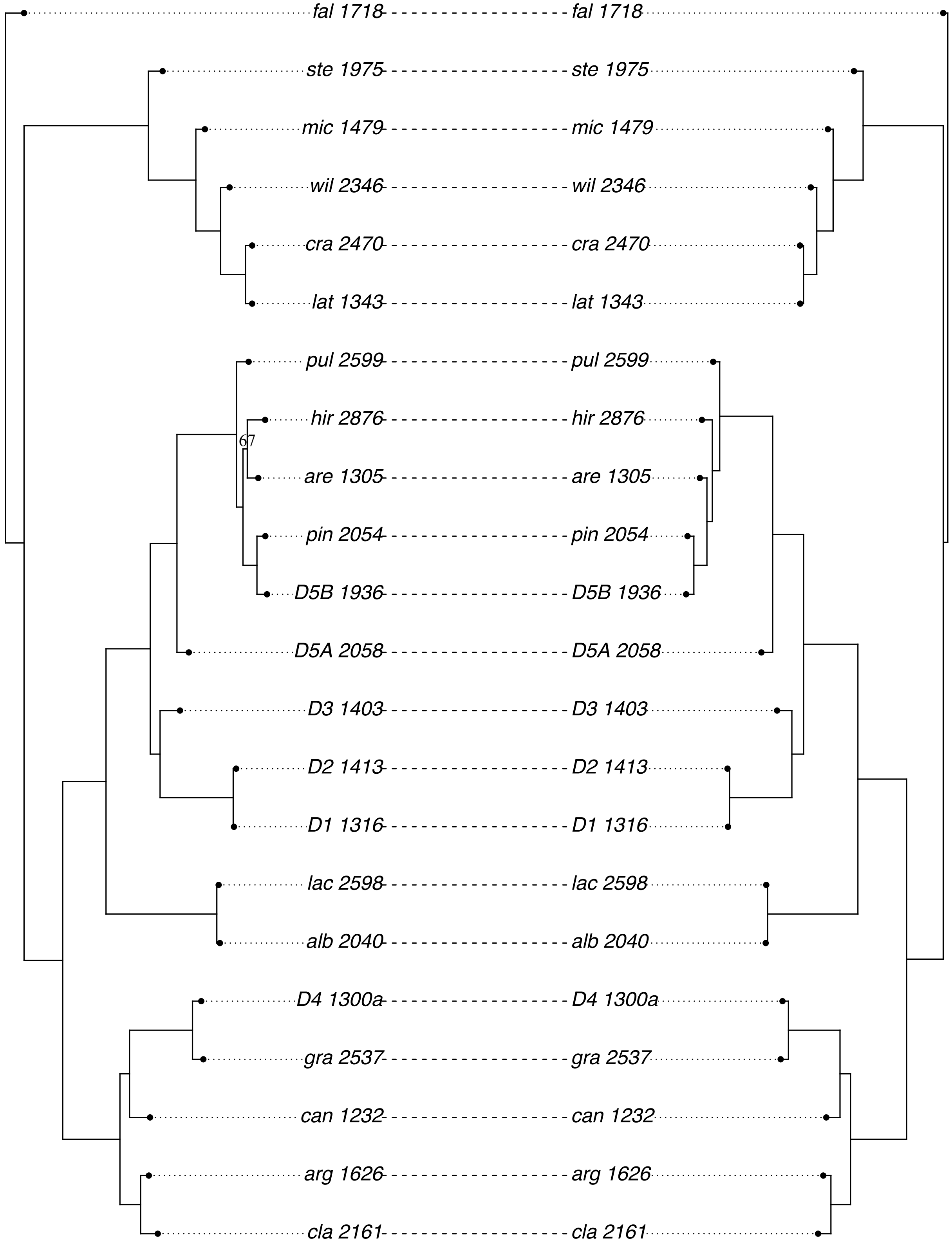

DOI 10.1002/aps3.11611
Appendix S3. Inferred topologies from all empirical datasets

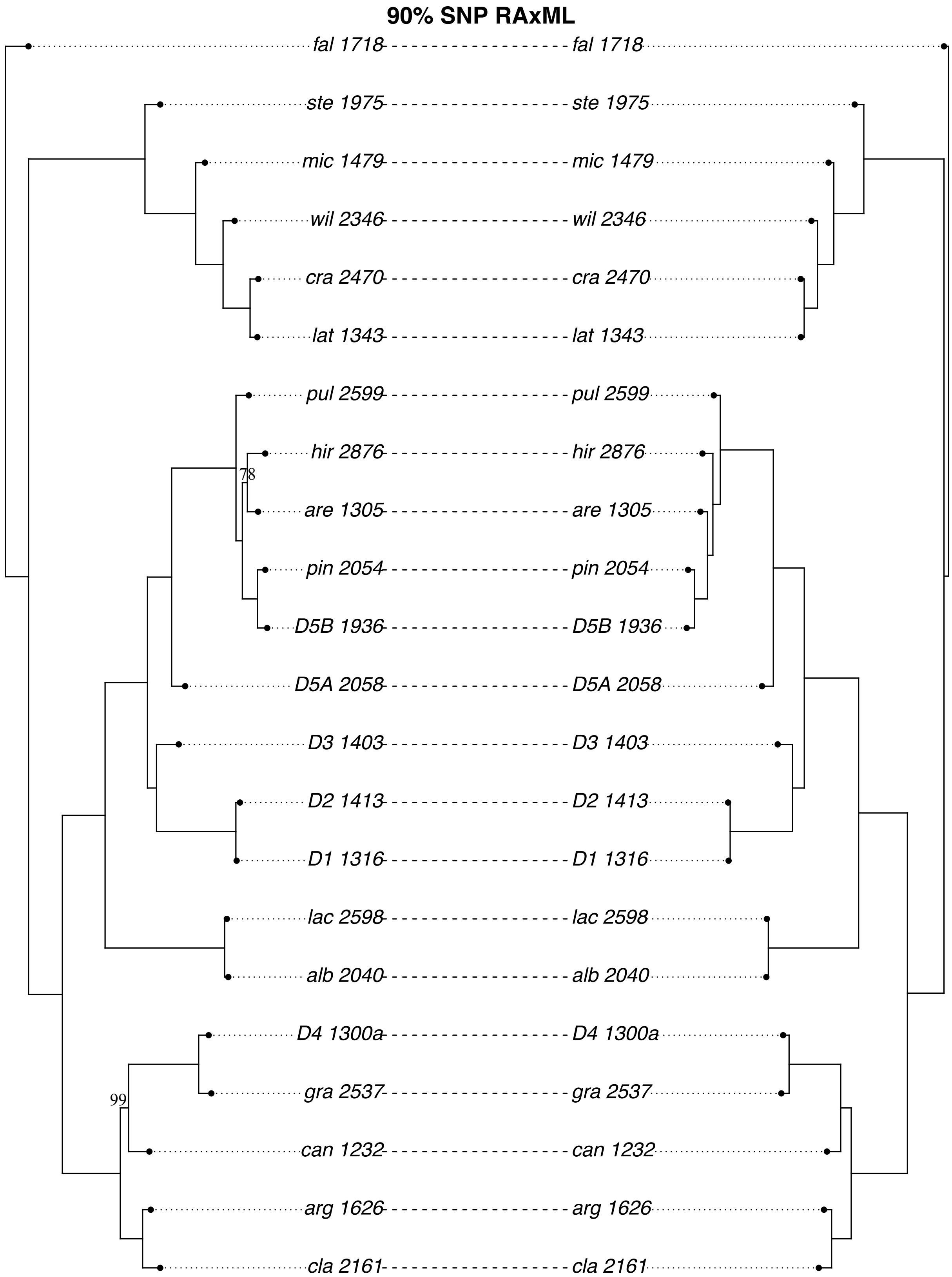

DOI 10.1002/aps3.11611
Appendix S3. Inferred topologies from all empirical datasets

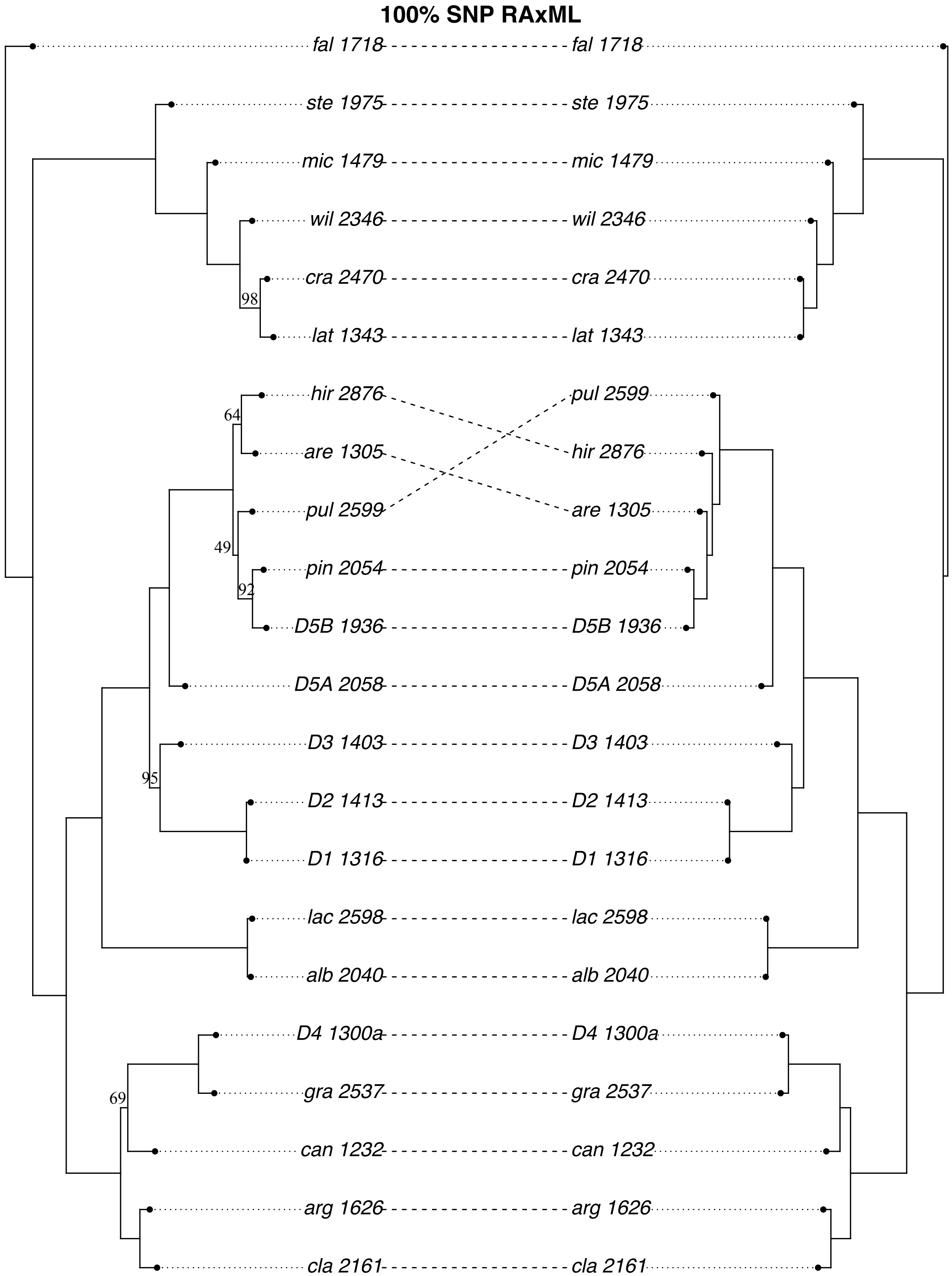

DOI 10.1002/aps3.11611

0% Locus RAxML

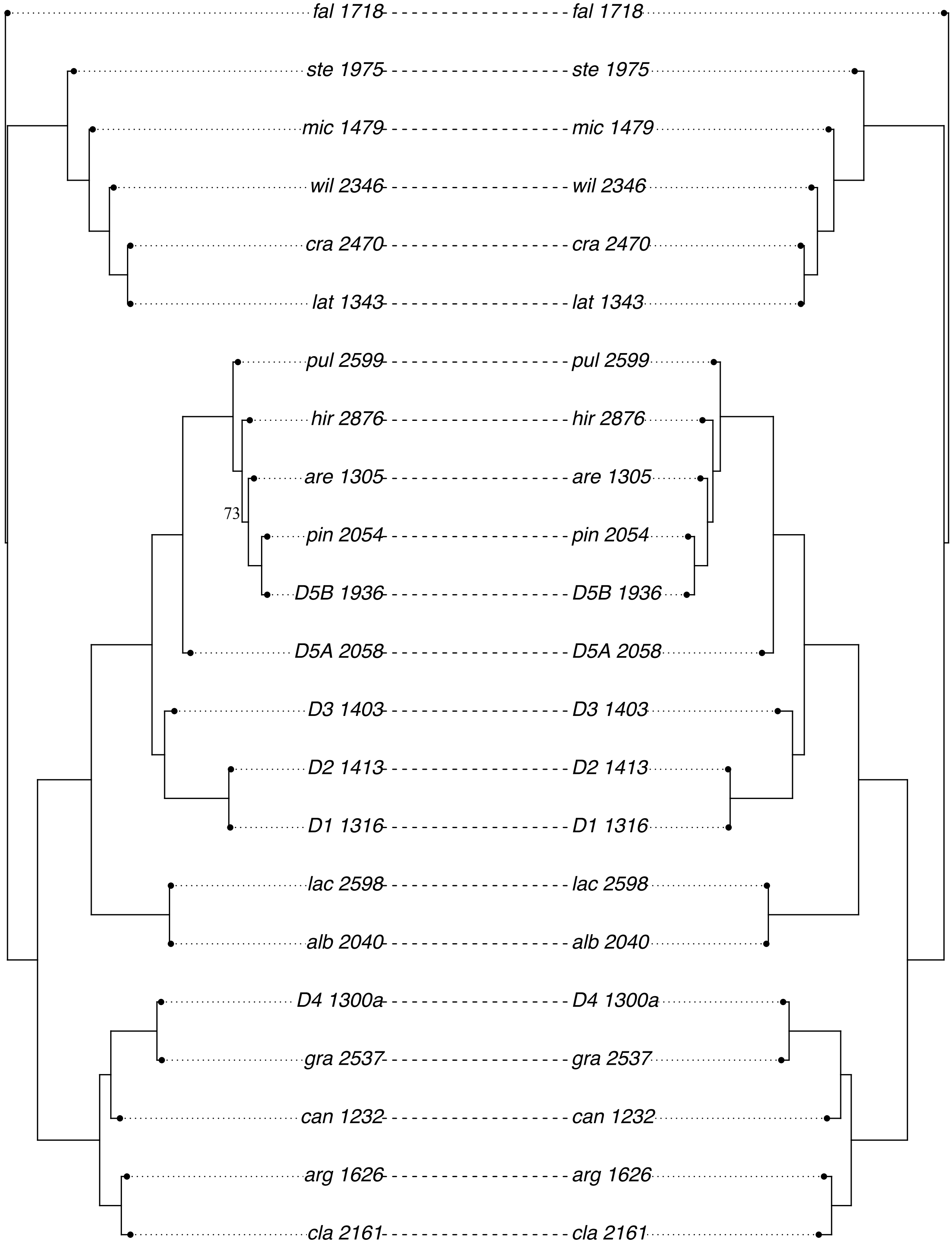

DOI 10.1002/aps3.11611
Appendix S3. Inferred topologies from all empirical datasets

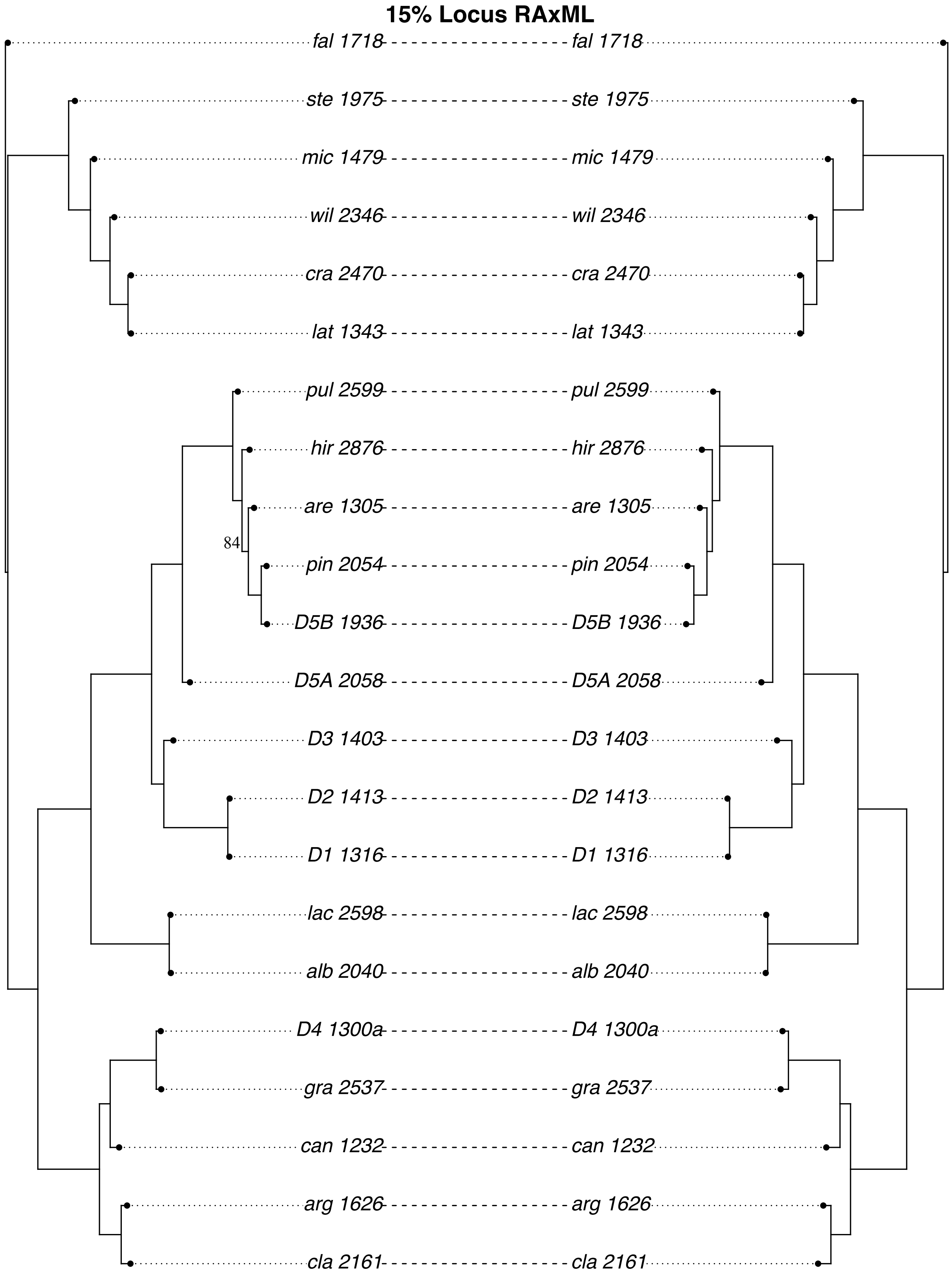

DOI 10.1002/aps3.11611
Appendix S3. Inferred topologies from all empirical datasets

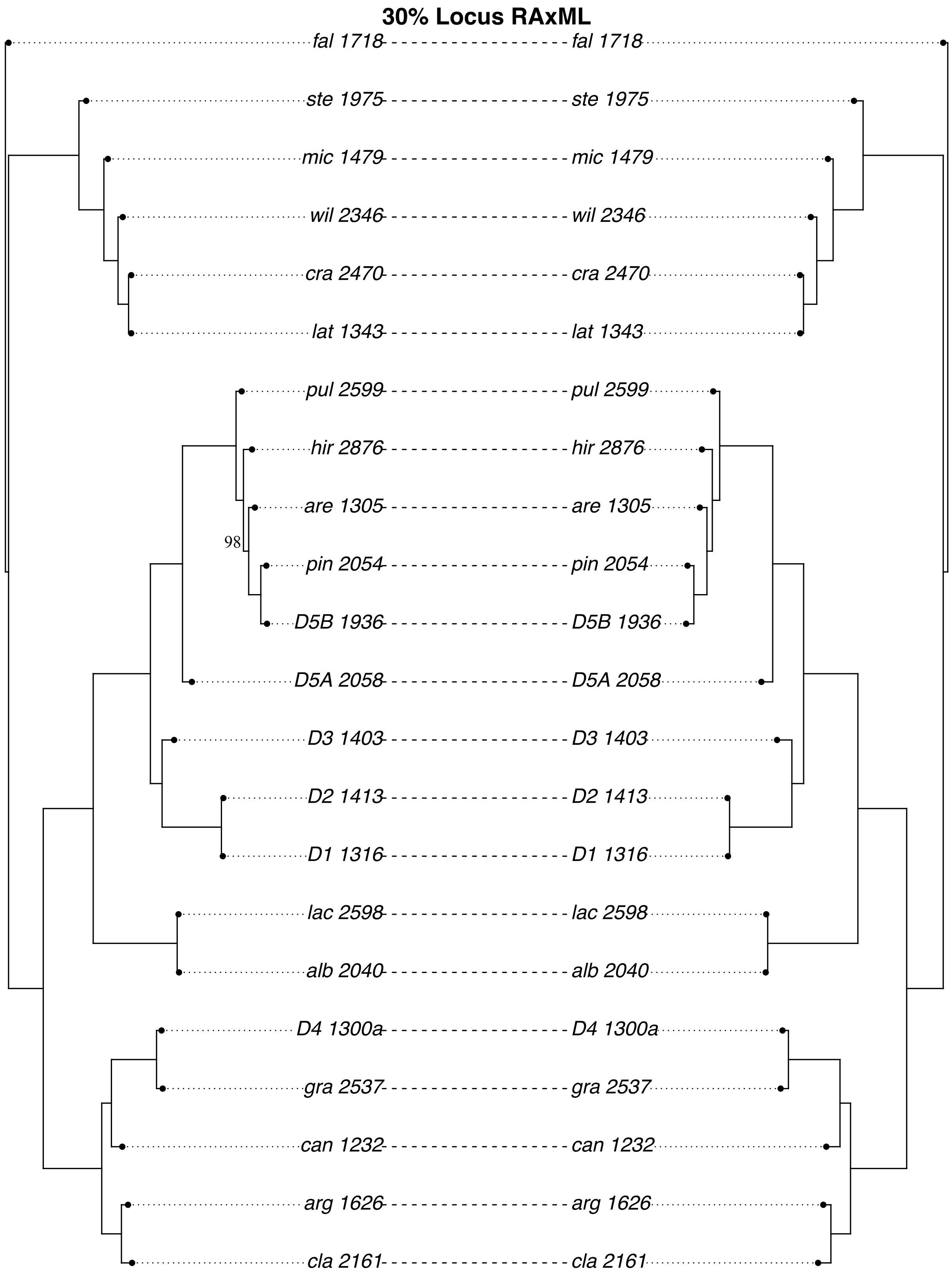

DOI 10.1002/aps3.11611
Appendix S3. Inferred topologies from all empirical datasets

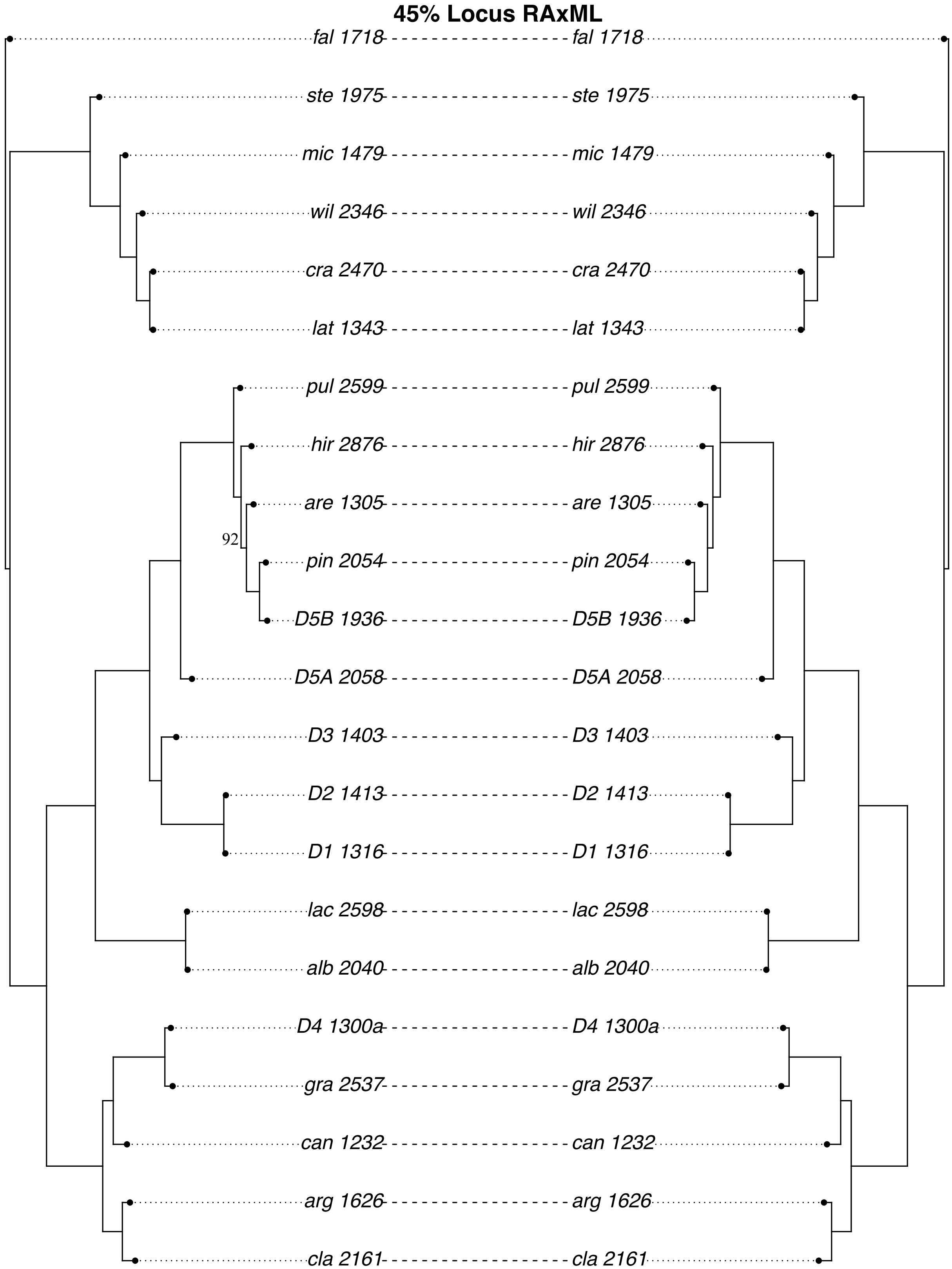

DOI 10.1002/aps3.11611
Appendix S3. Inferred topologies from all empirical datasets

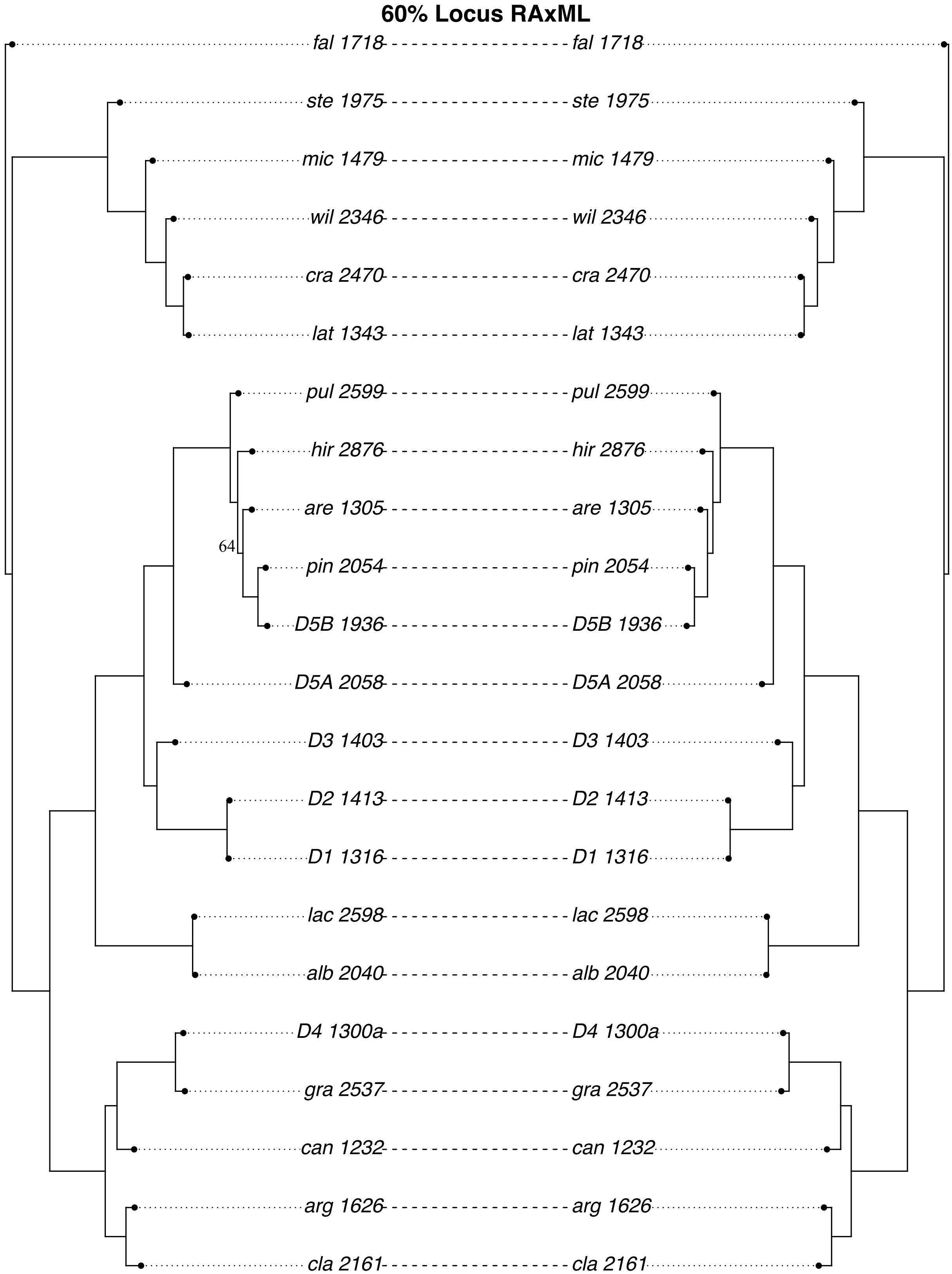

DOI 10.1002/aps3.11611
Appendix S3. Inferred topologies from all empirical datasets

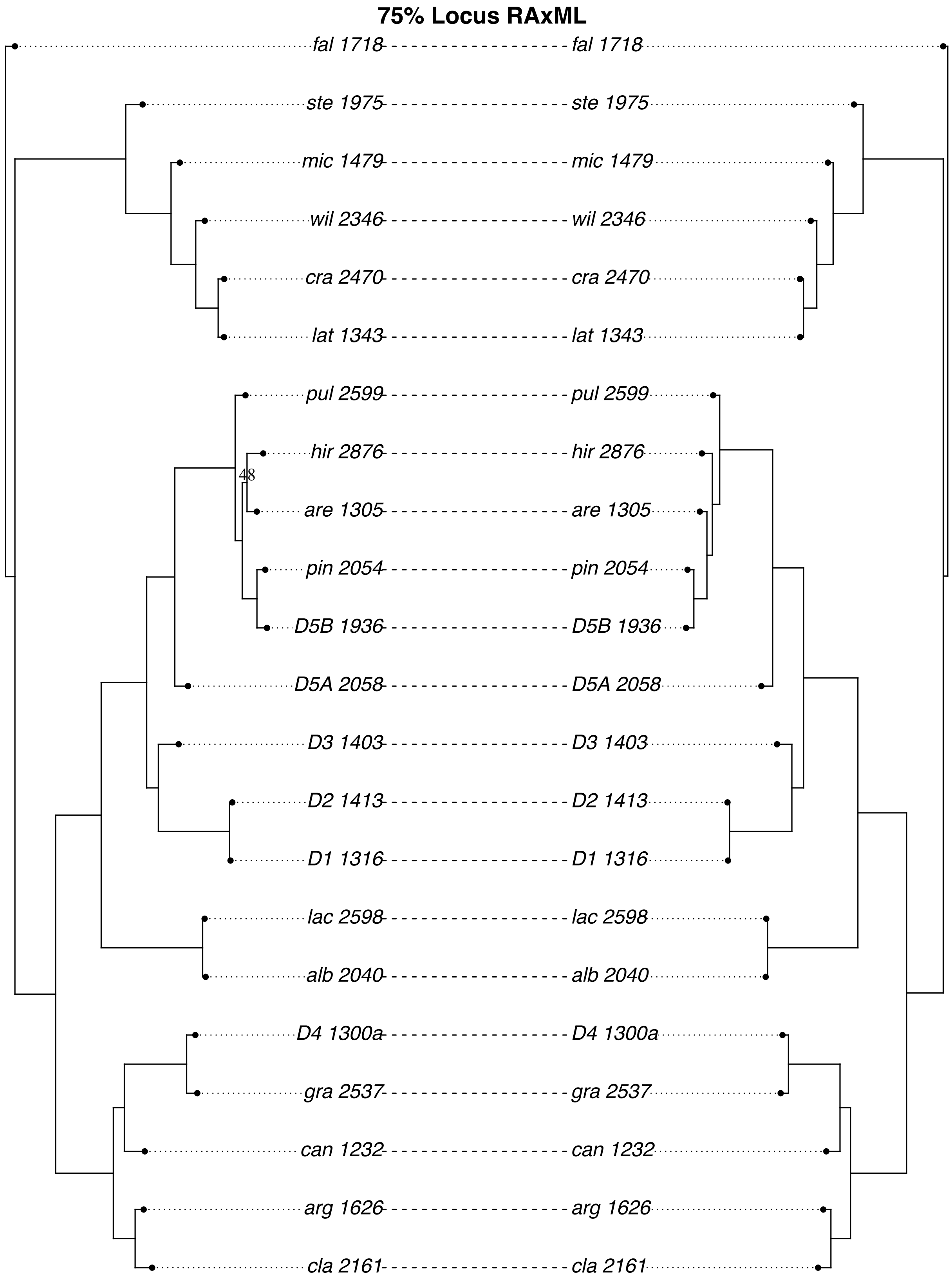

DOI 10.1002/aps3.11611
Appendix S3. Inferred topologies from all empirical datasets

90% Locus RAxML

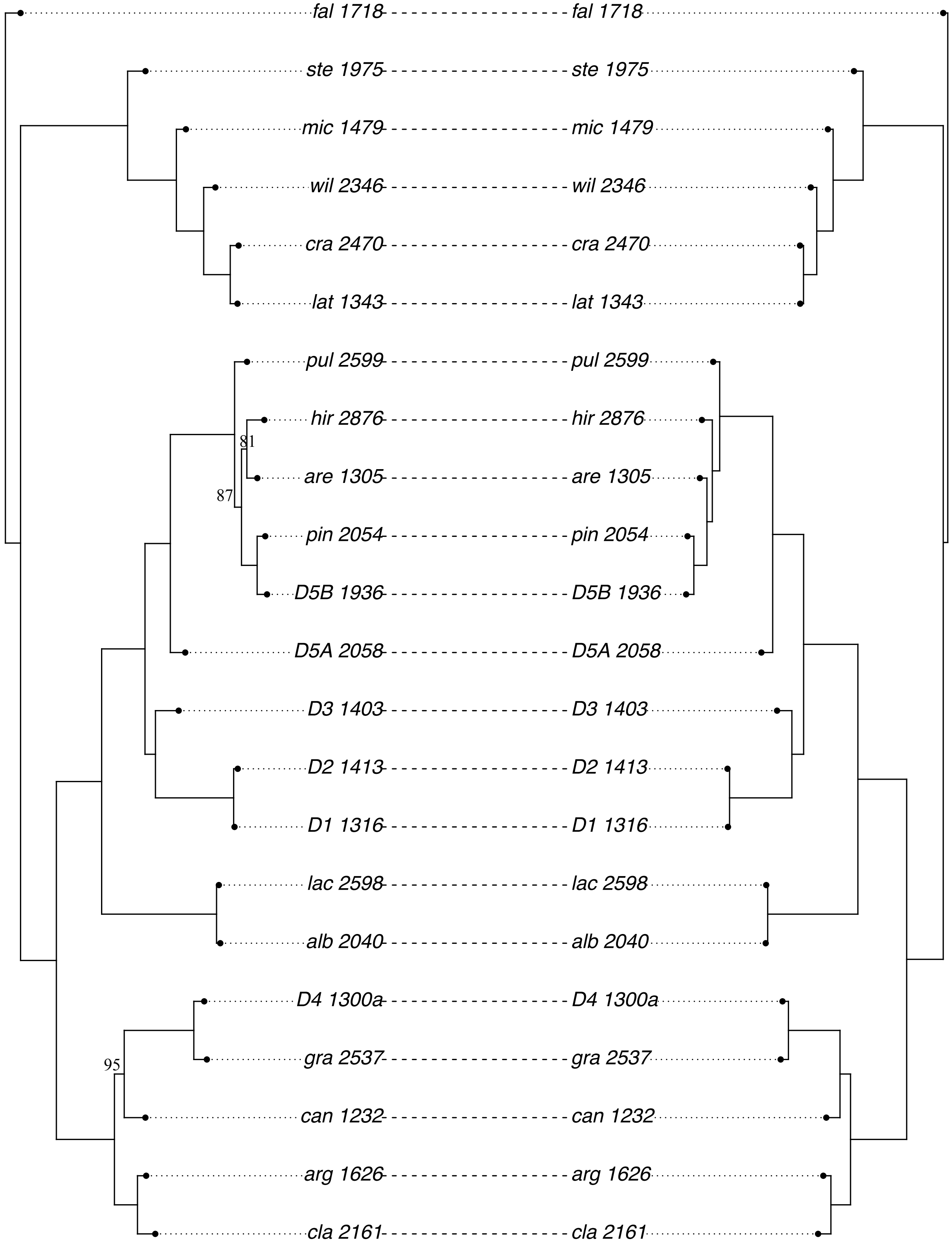

DOI 10.1002/aps3.11611
Appendix S3. Inferred topologies from all empirical datasets

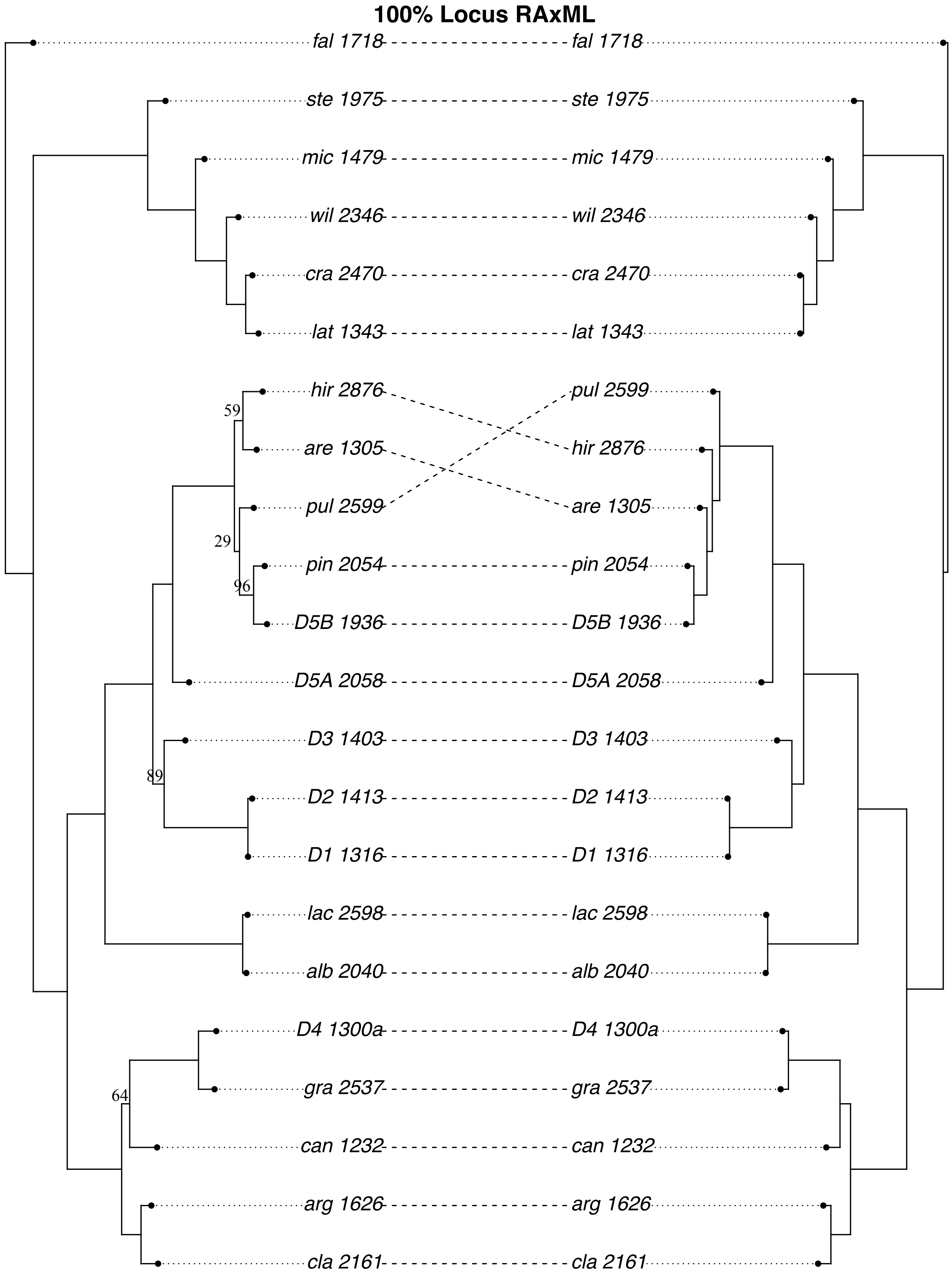

Supplement: Supplementary file 3 — Appendix S3. Inferred topologies from all empirical datasets including different filtering thresholds and analysis methods. The right side of the cophylo plot is the 45% RAxML tree as a reference. Bootstrap support or posterior probability at nodes are 100%/1.0 unless otherwise specified. [file APS3-12-e11611-s007.pdf]
