# Supplementary material for: Data‐driven guidelines for phylogenomic analyses using SNP data
Source: Appl Plant Sci. 2024 Aug 9;12(6):e11611. doi: 10.1002/aps3.11611 (PMC11610416; doi:10.1002/aps3.11611)

DOI 10.1002/aps3.11611

Appendix S6. Inferred topologies and divergence time estimation with the 95% confidence interval of node ages from all empirical datasets including different filtering thresholds and analysis methods.

0% variant SNP filtering threshold BEAST MCC

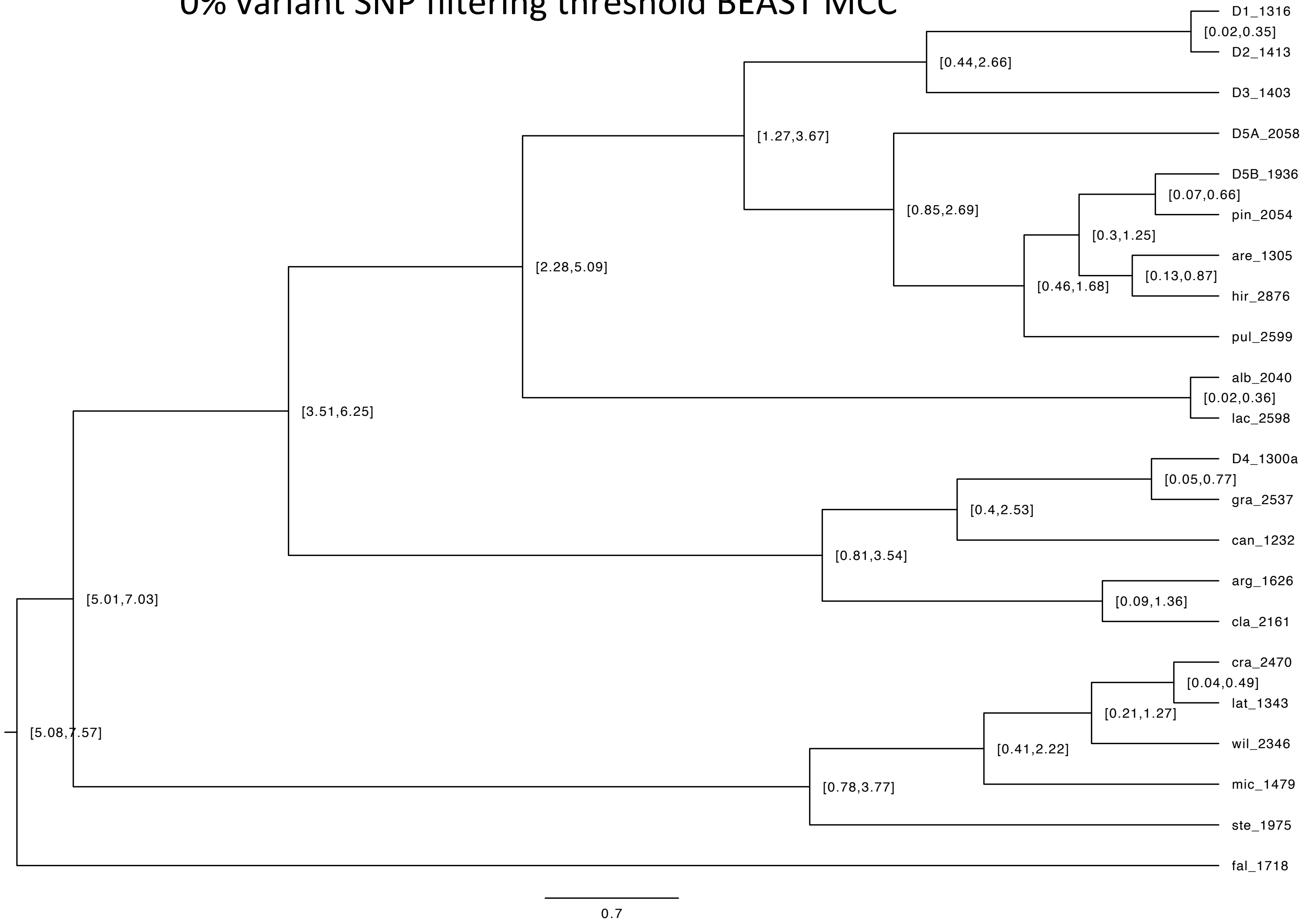

DOI 10.1002/aps3.11611

15% all SNP filtering threshold BEAST MCC

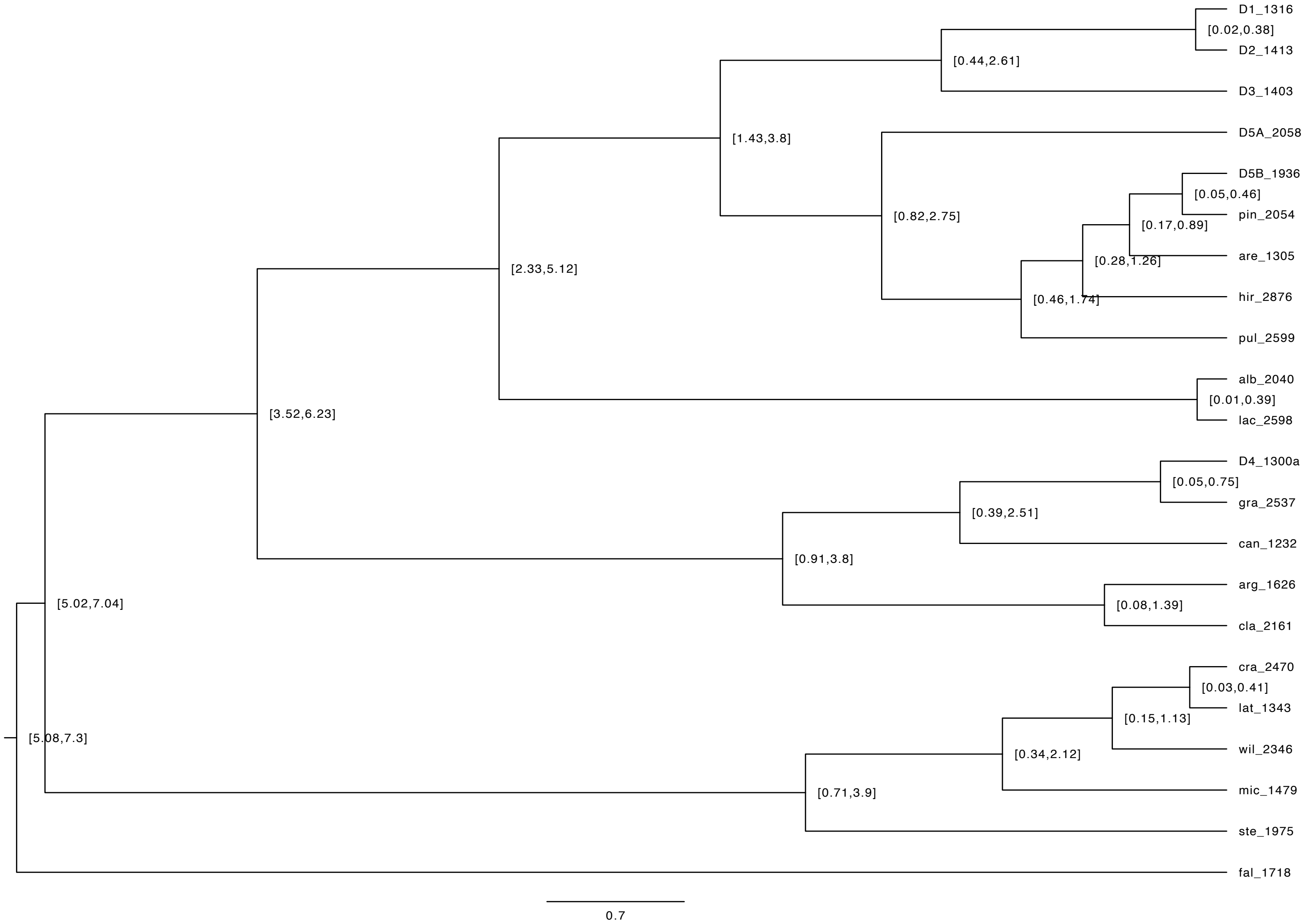

DOI 10.1002/aps3.11611

15% variant SNP filtering threshold BEAST MCC

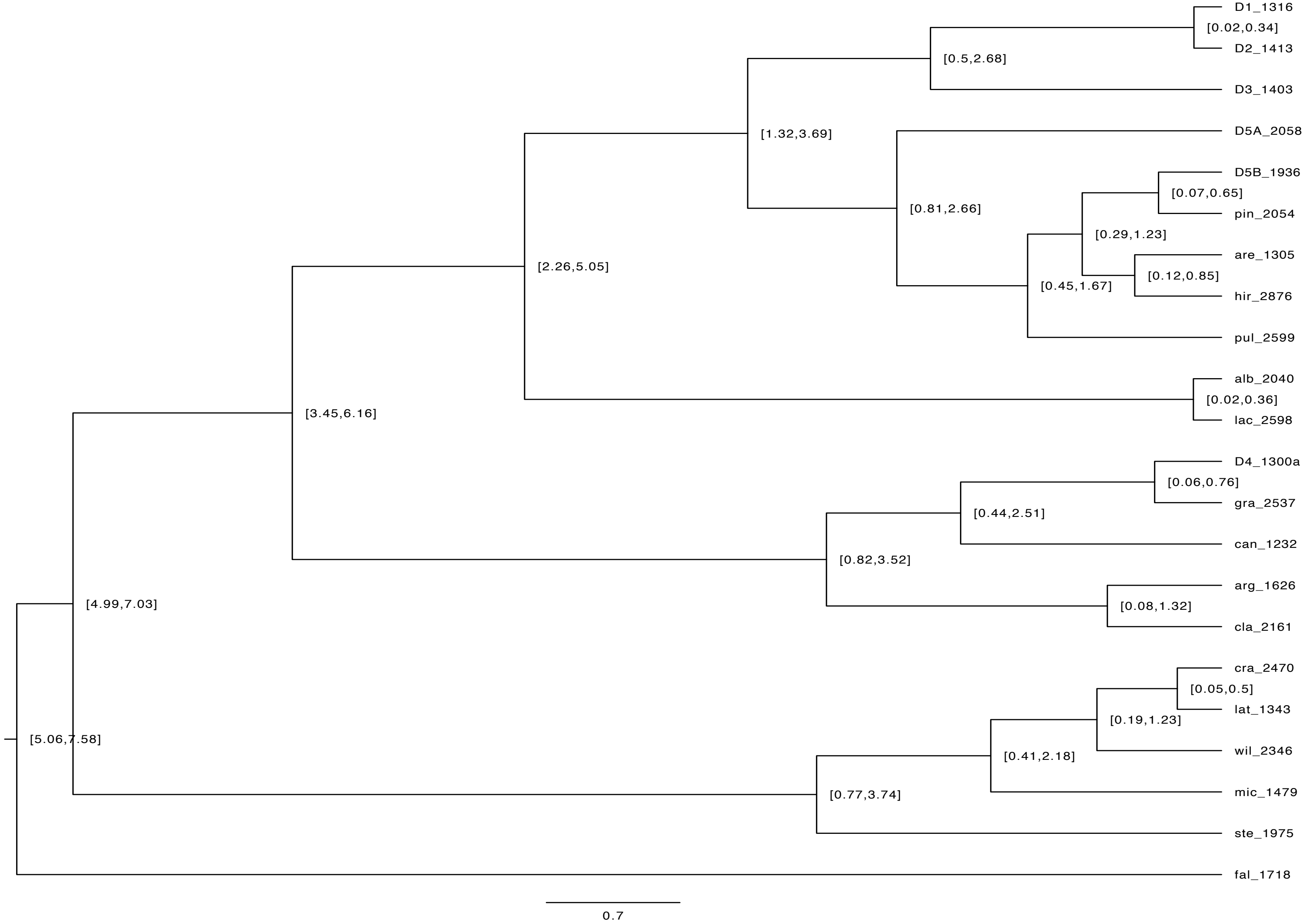

DOI 10.1002/aps3.11611

30% all SNP filtering threshold BEAST MCC

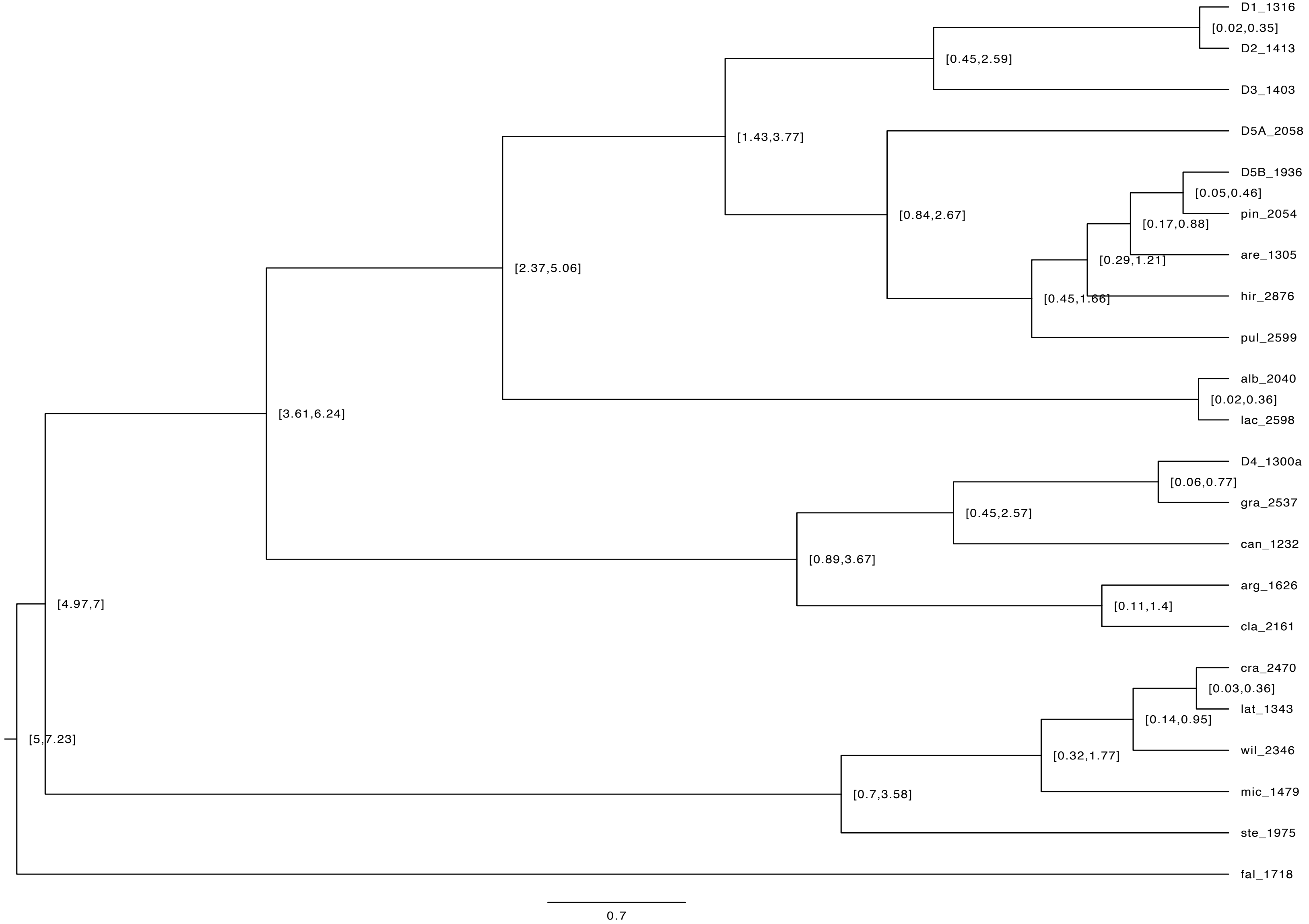

DOI 10.1002/aps3.11611

30% variant SNP filtering threshold BEAST MCC

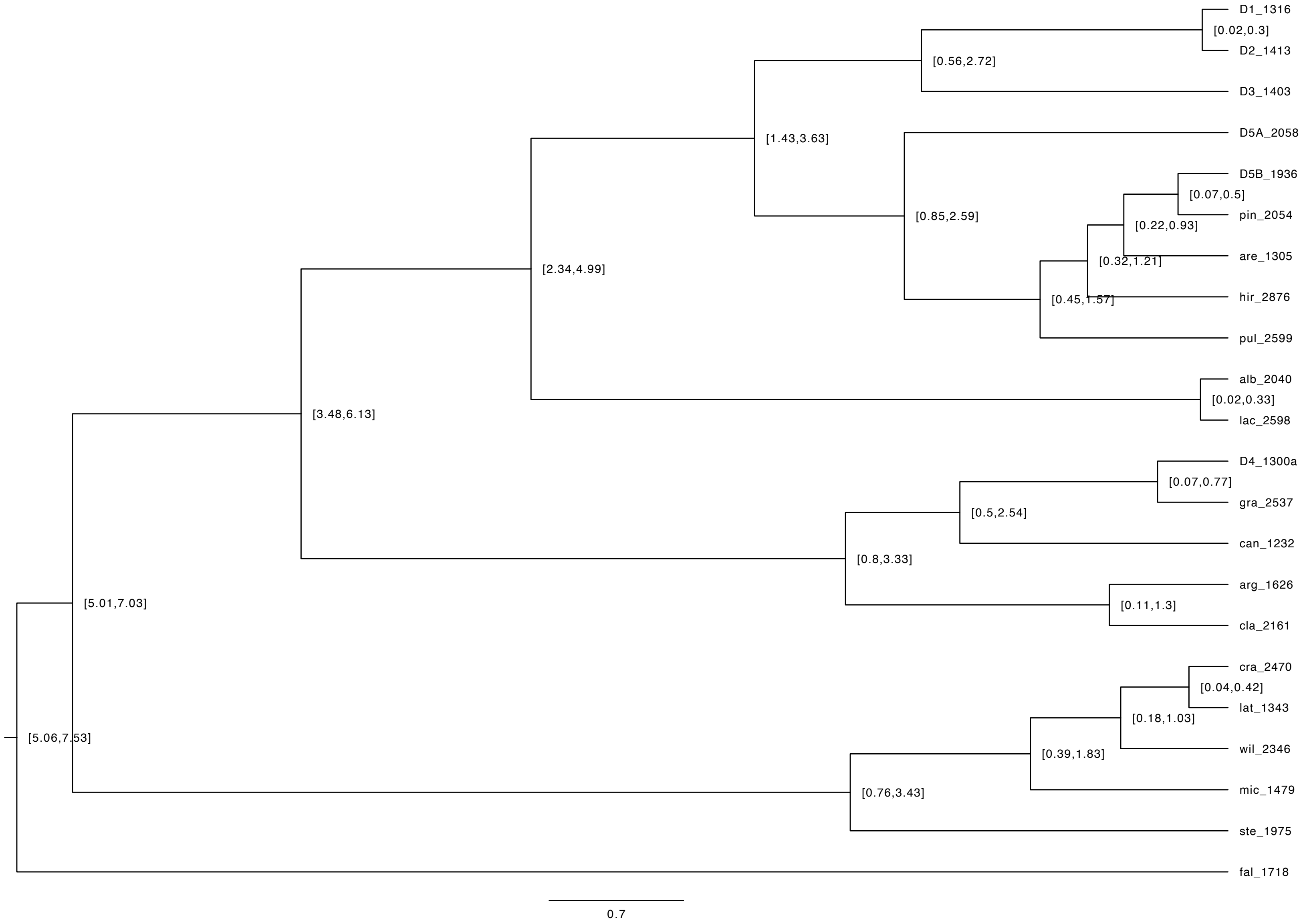

DOI 10.1002/aps3.11611

45% all SNP filtering threshold BEAST MCC

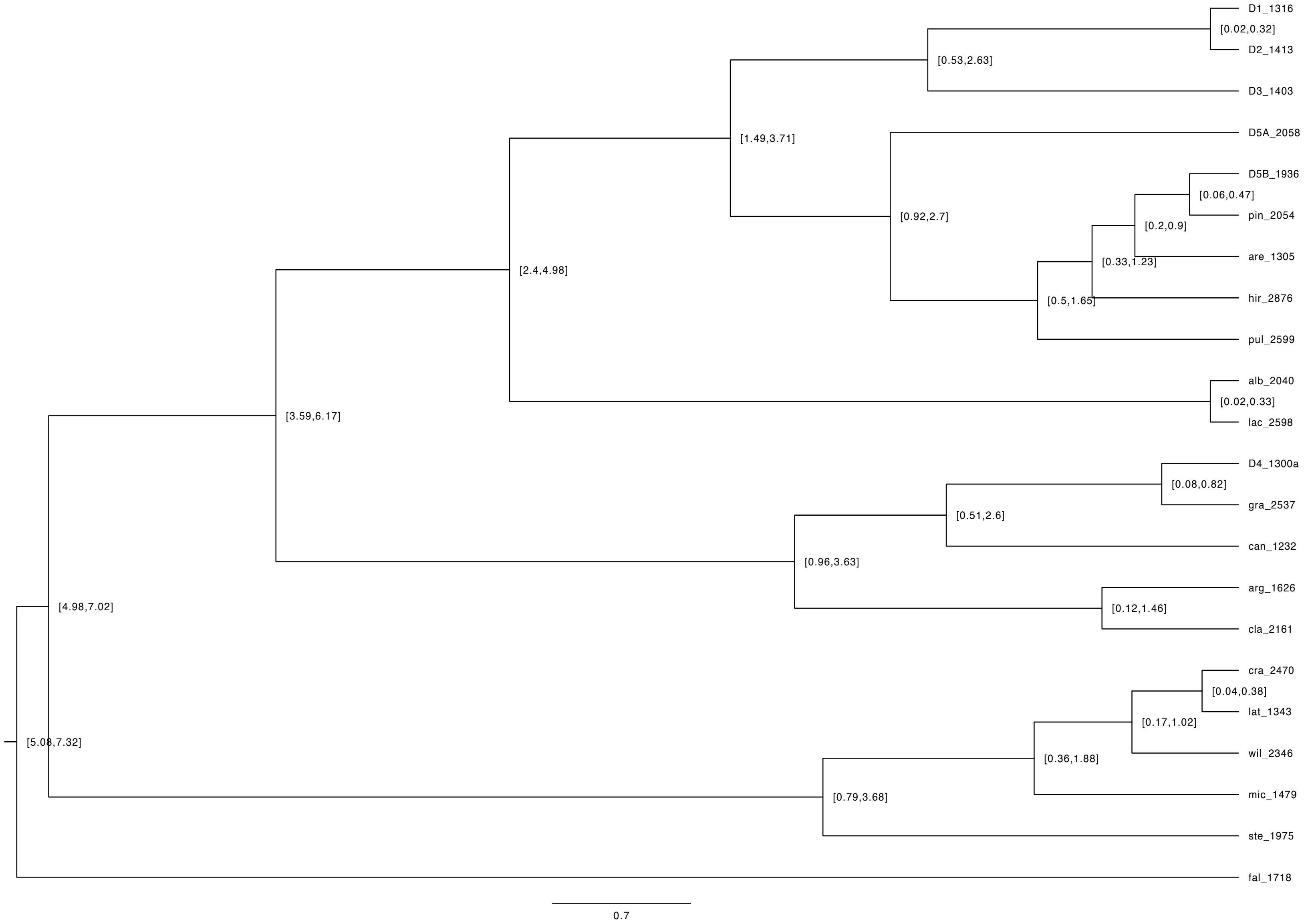

DOI 10.1002/aps3.11611

45% variant SNP filtering threshold BEAST MCC

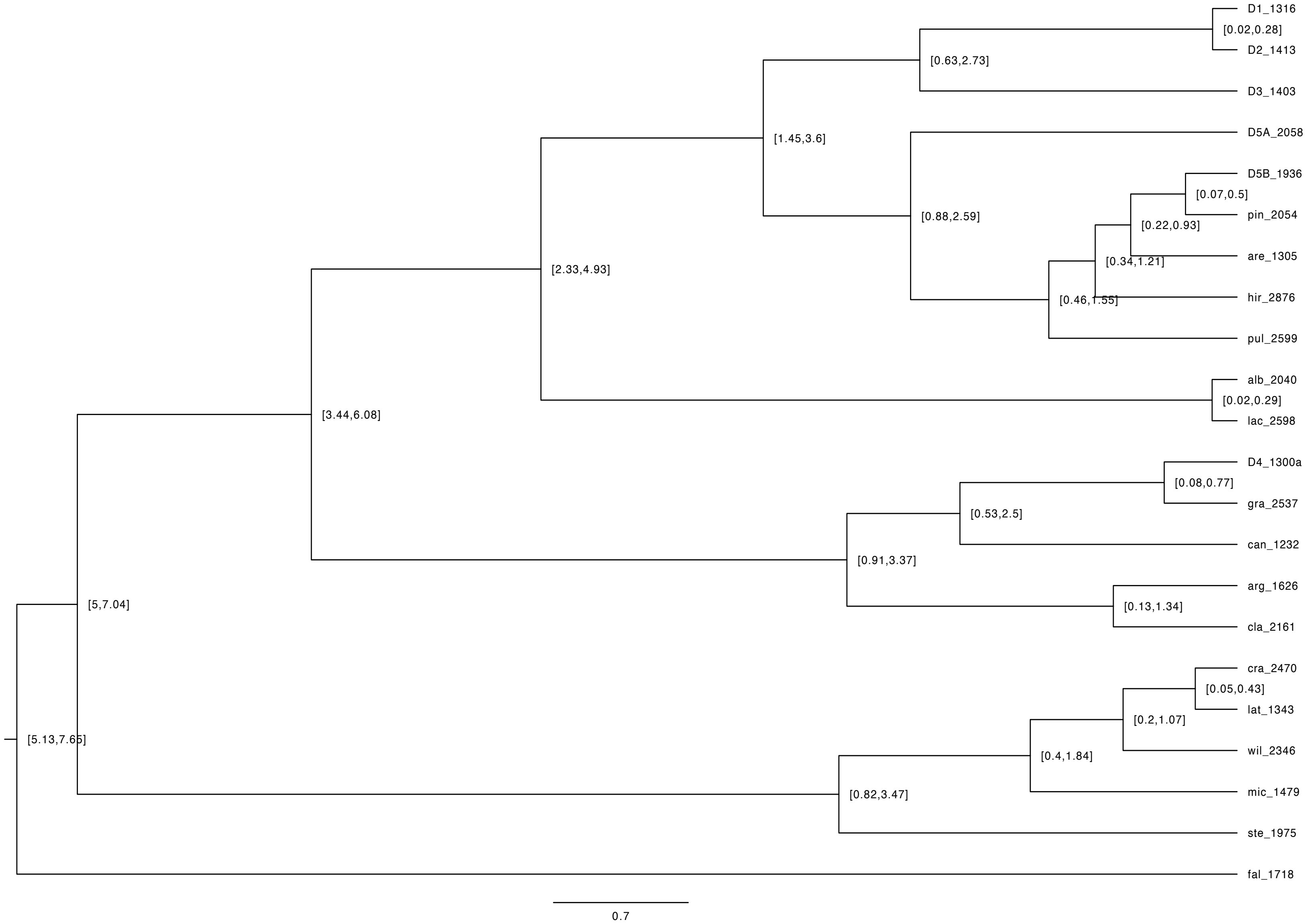

DOI 10.1002/aps3.11611

60% all SNP filtering threshold BEAST MCC

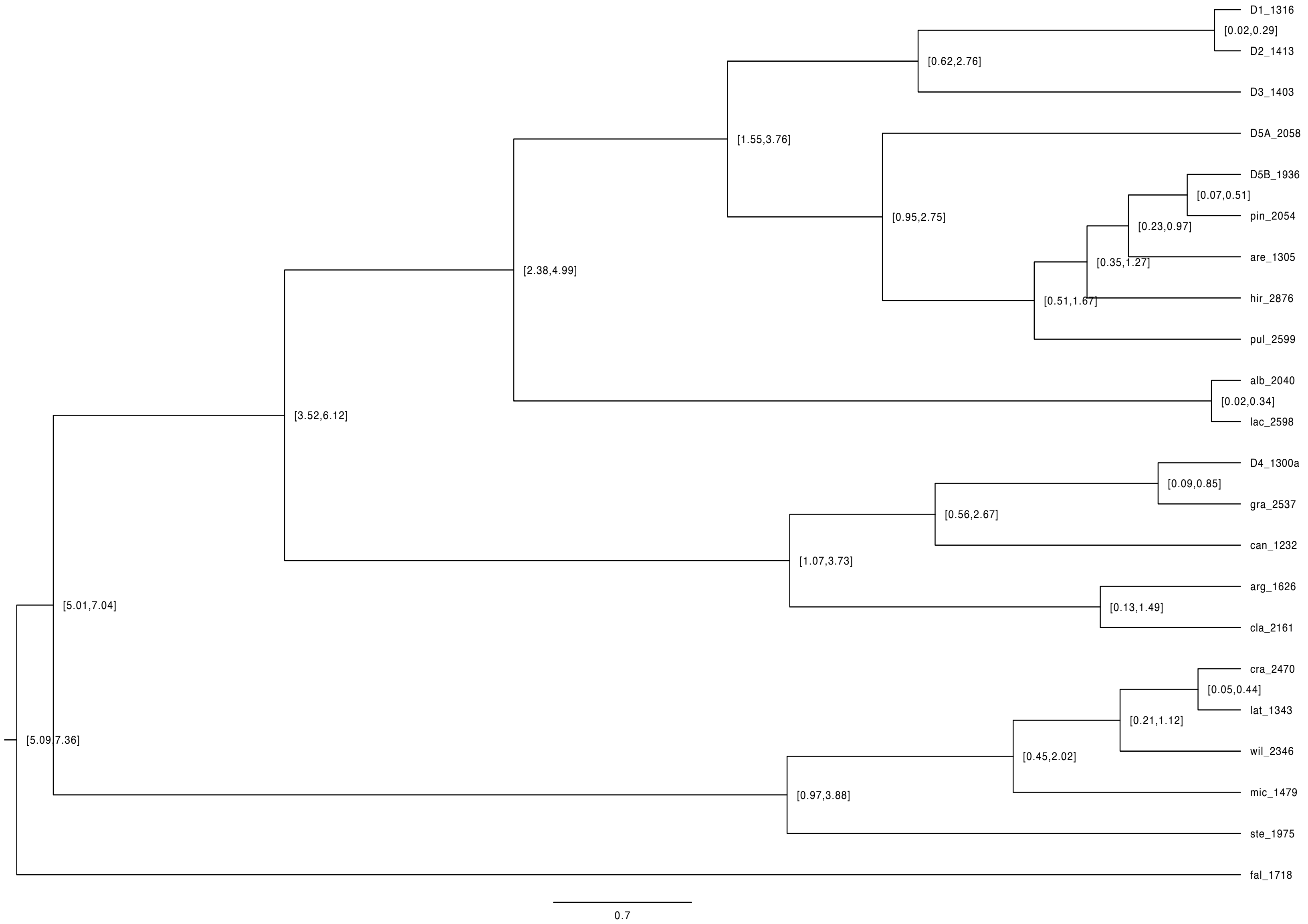

DOI 10.1002/aps3.11611

60% variant SNP filtering threshold BEAST MCC

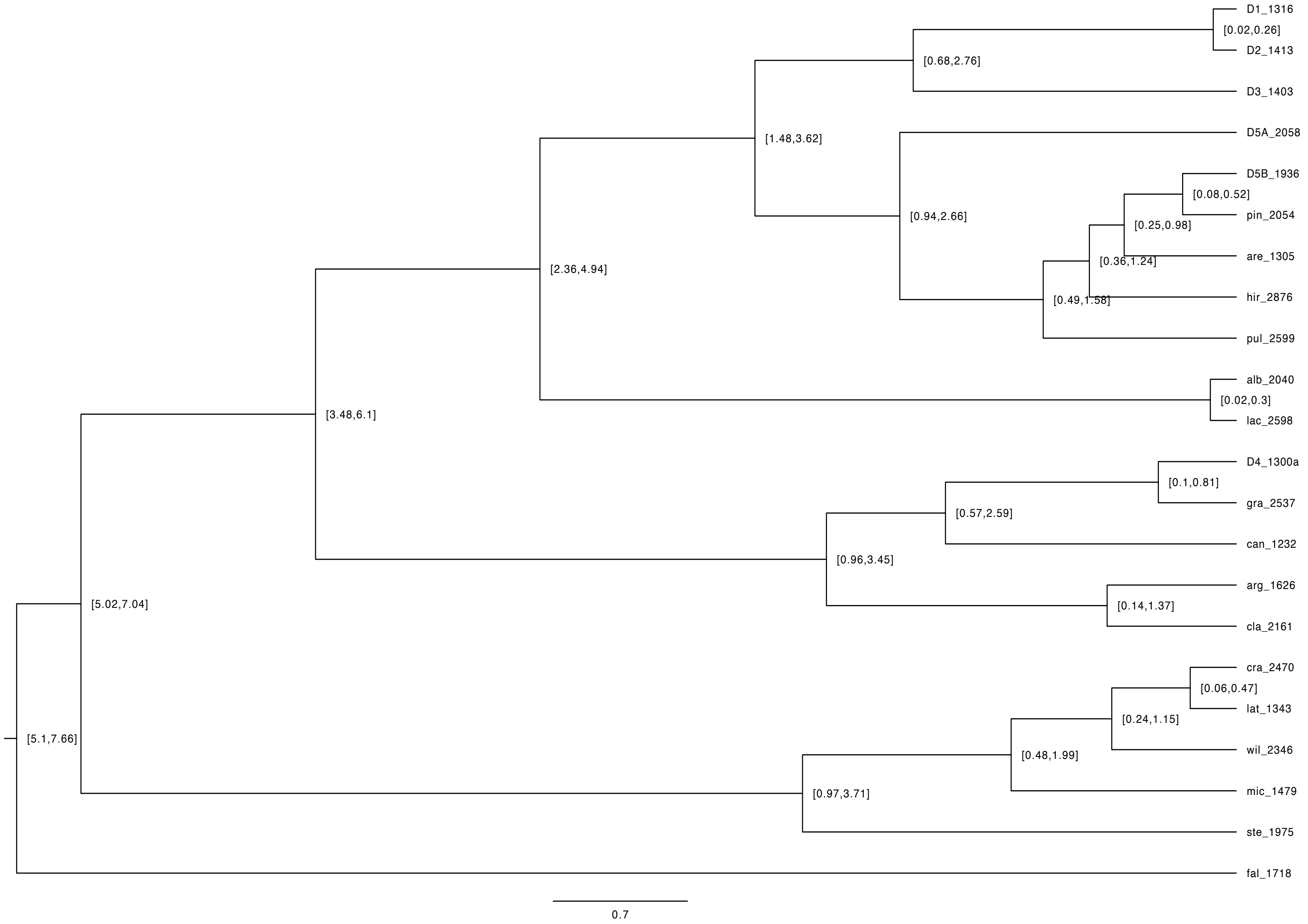

DOI 10.1002/aps3.11611

75% all SNP filtering threshold BEAST MCC

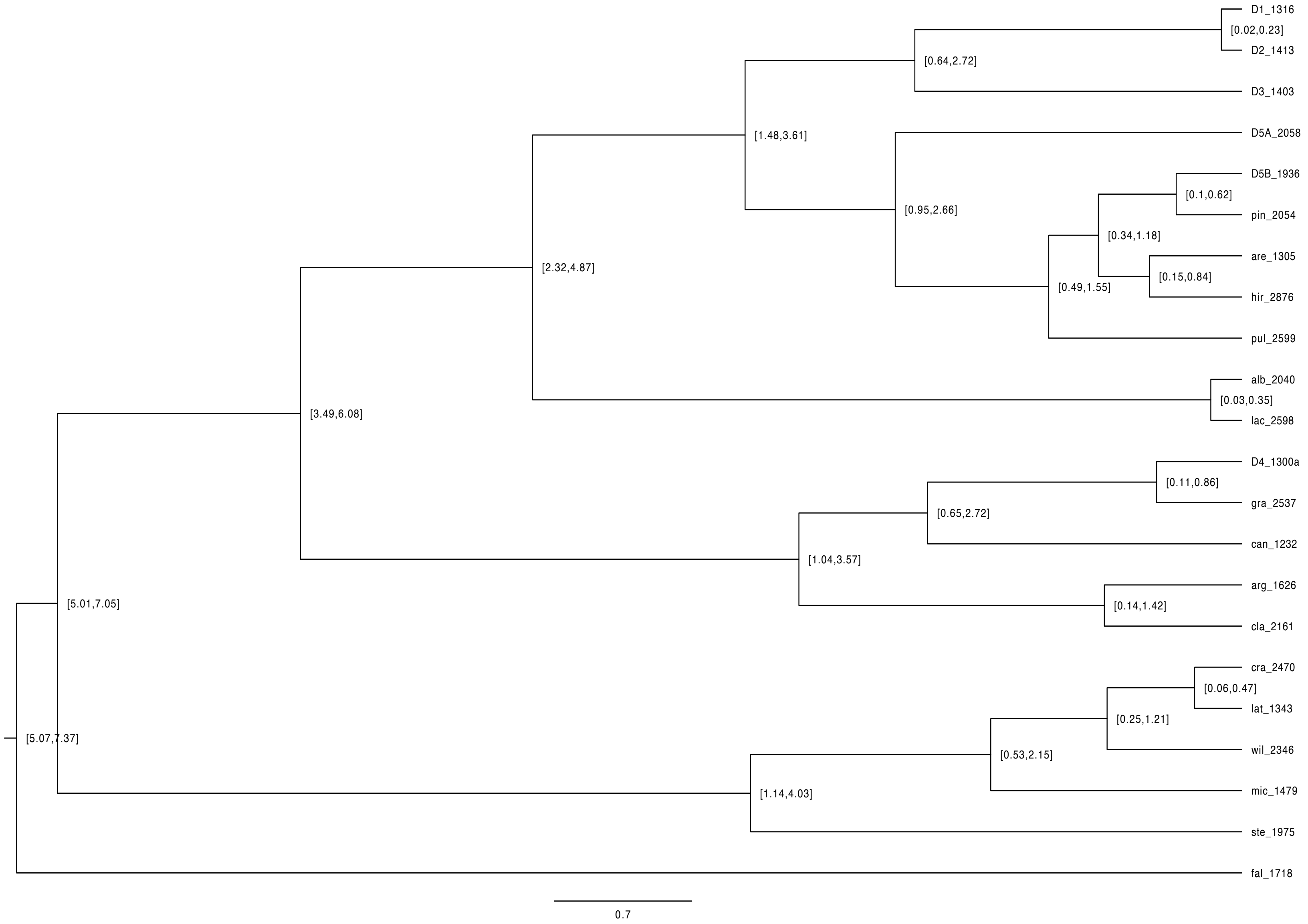

DOI 10.1002/aps3.11611

75% variant SNP filtering threshold BEAST MCC

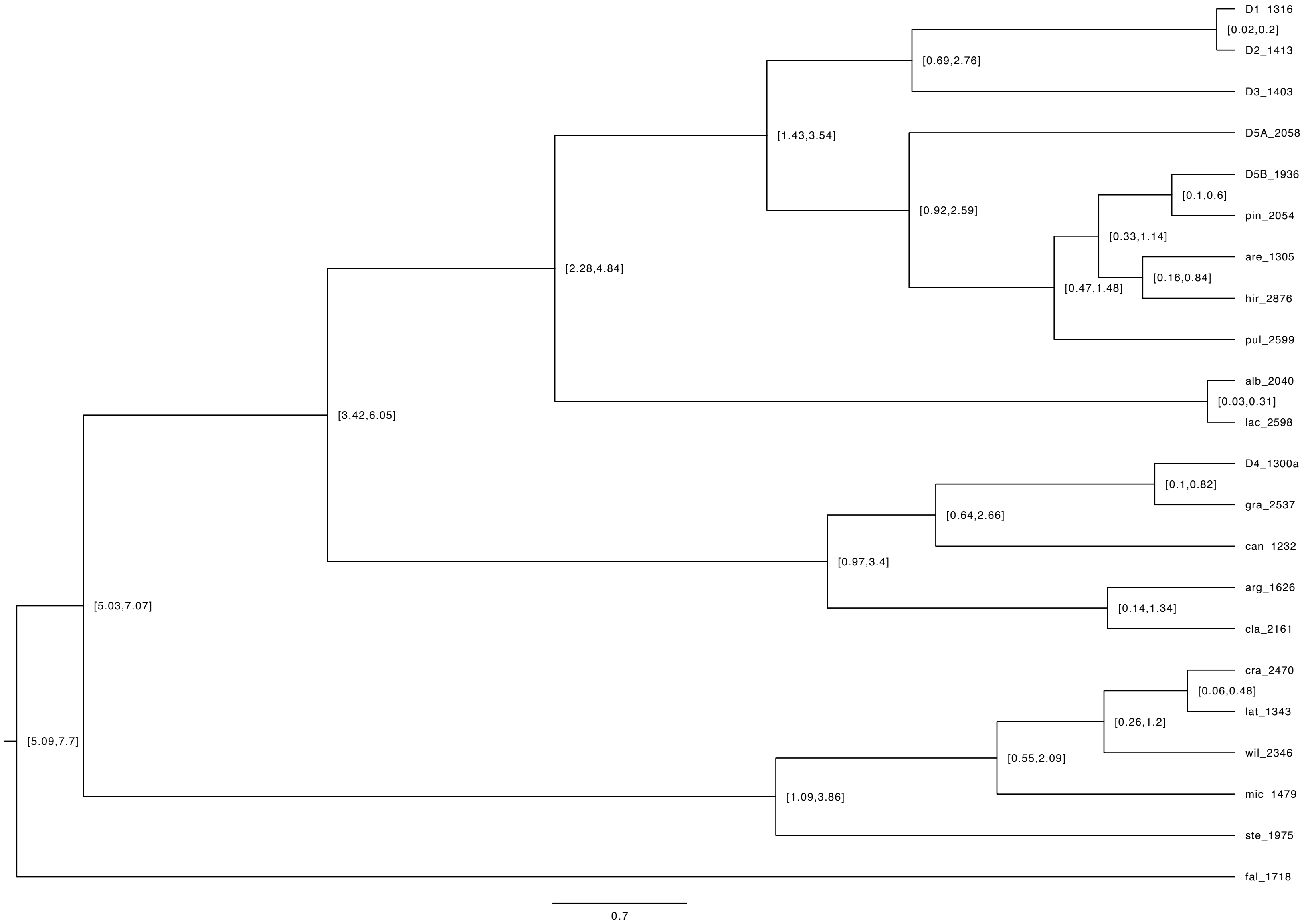

DOI 10.1002/aps3.11611

90% all SNP filtering threshold BEAST MCC

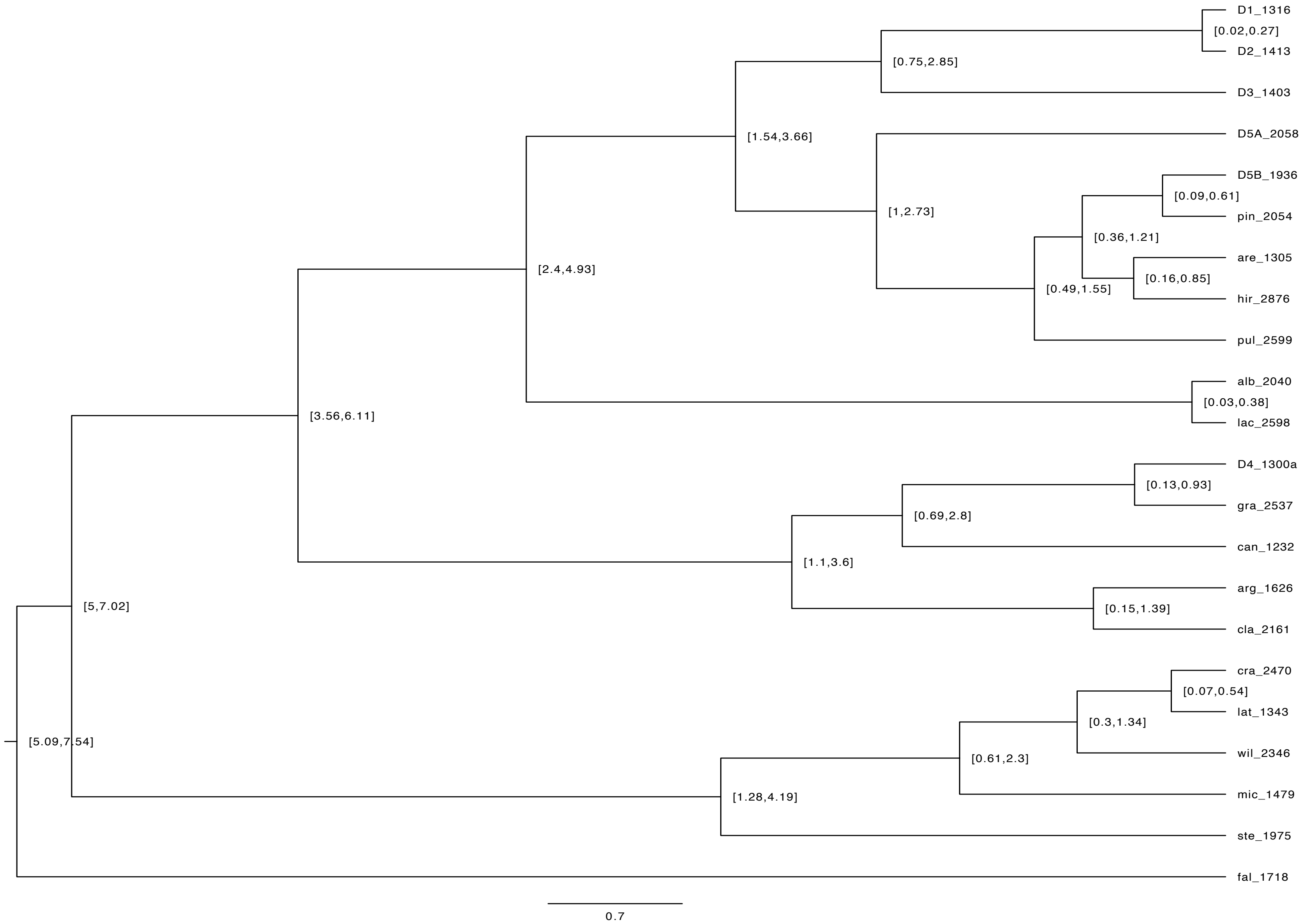

DOI 10.1002/aps3.11611

90% variant SNP filtering threshold BEAST MCC

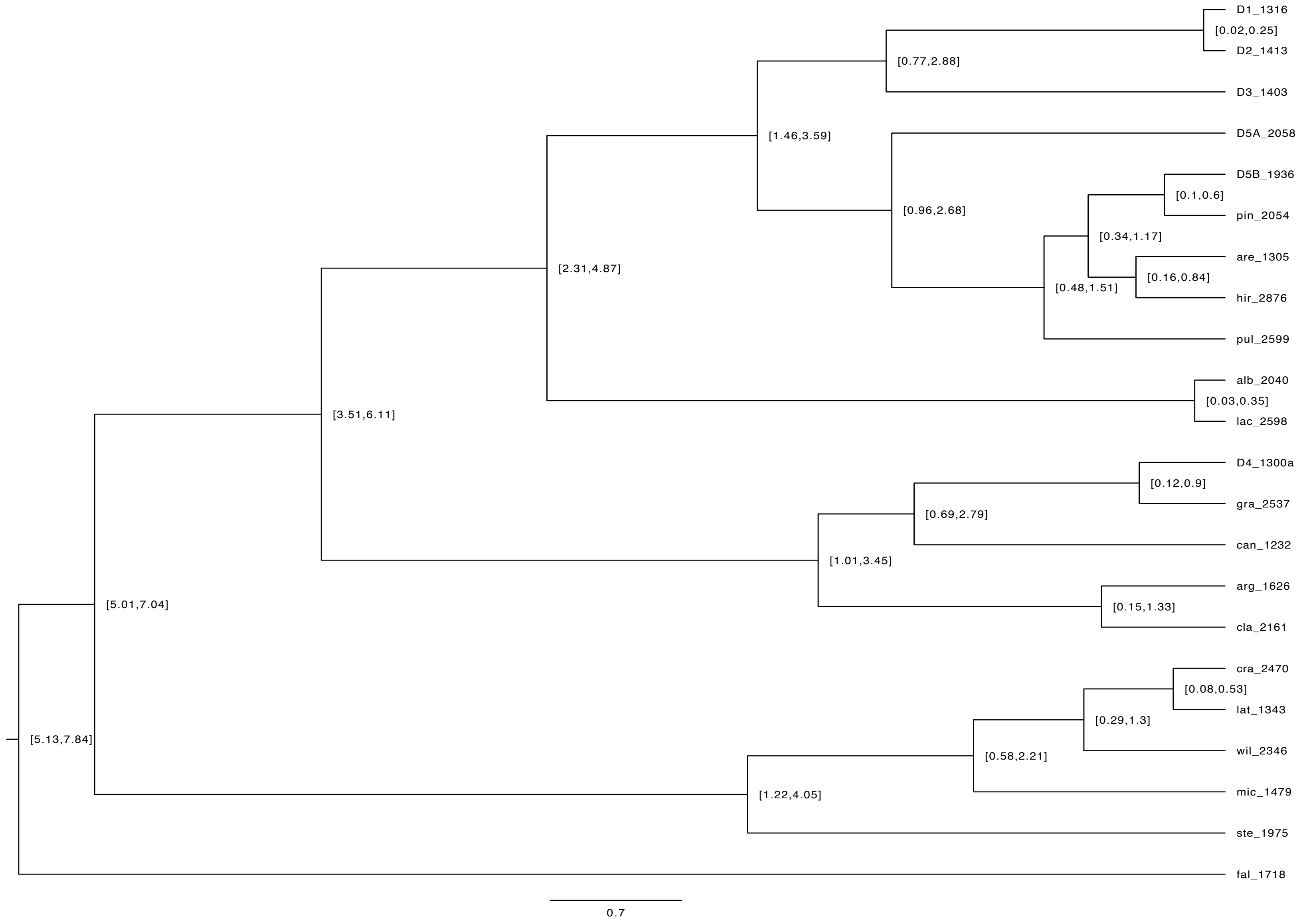

DOI 10.1002/aps3.11611

100% all SNP filtering threshold BEAST MCC

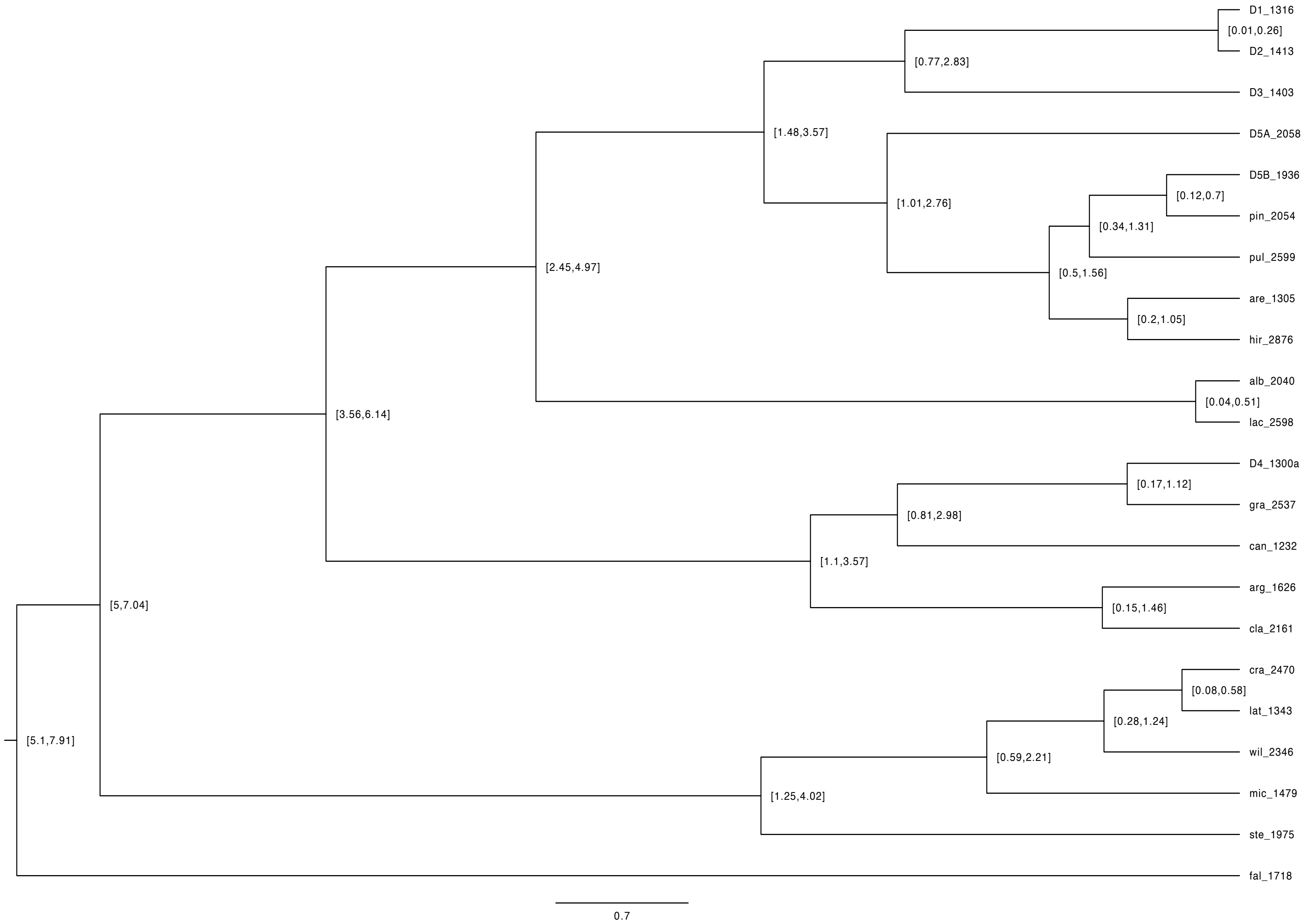

DOI 10.1002/aps3.11611

100% variant SNP filtering threshold BEAST MCC

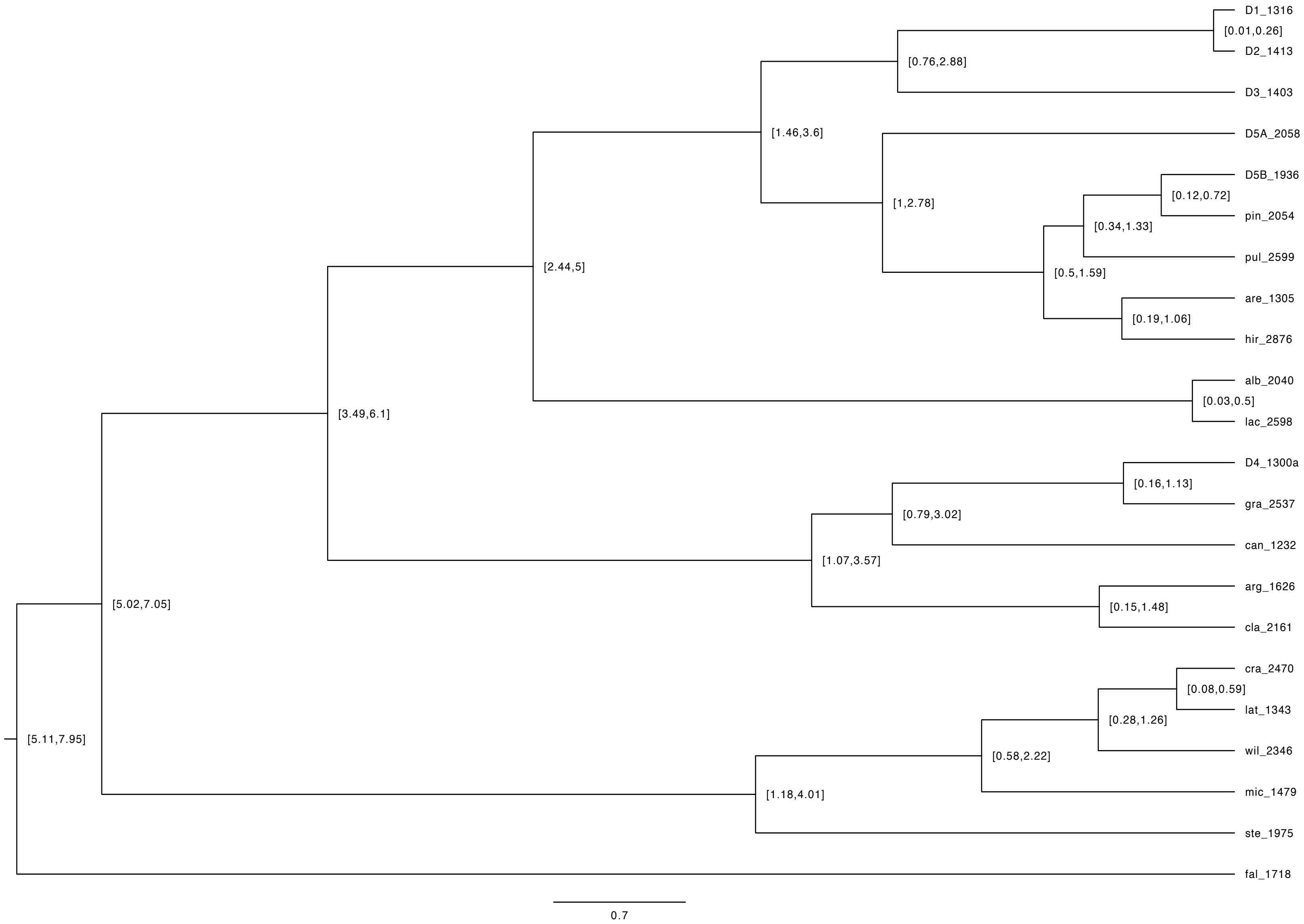

DOI 10.1002/aps3.11611

0% variant SNP filtering threshold RelTime

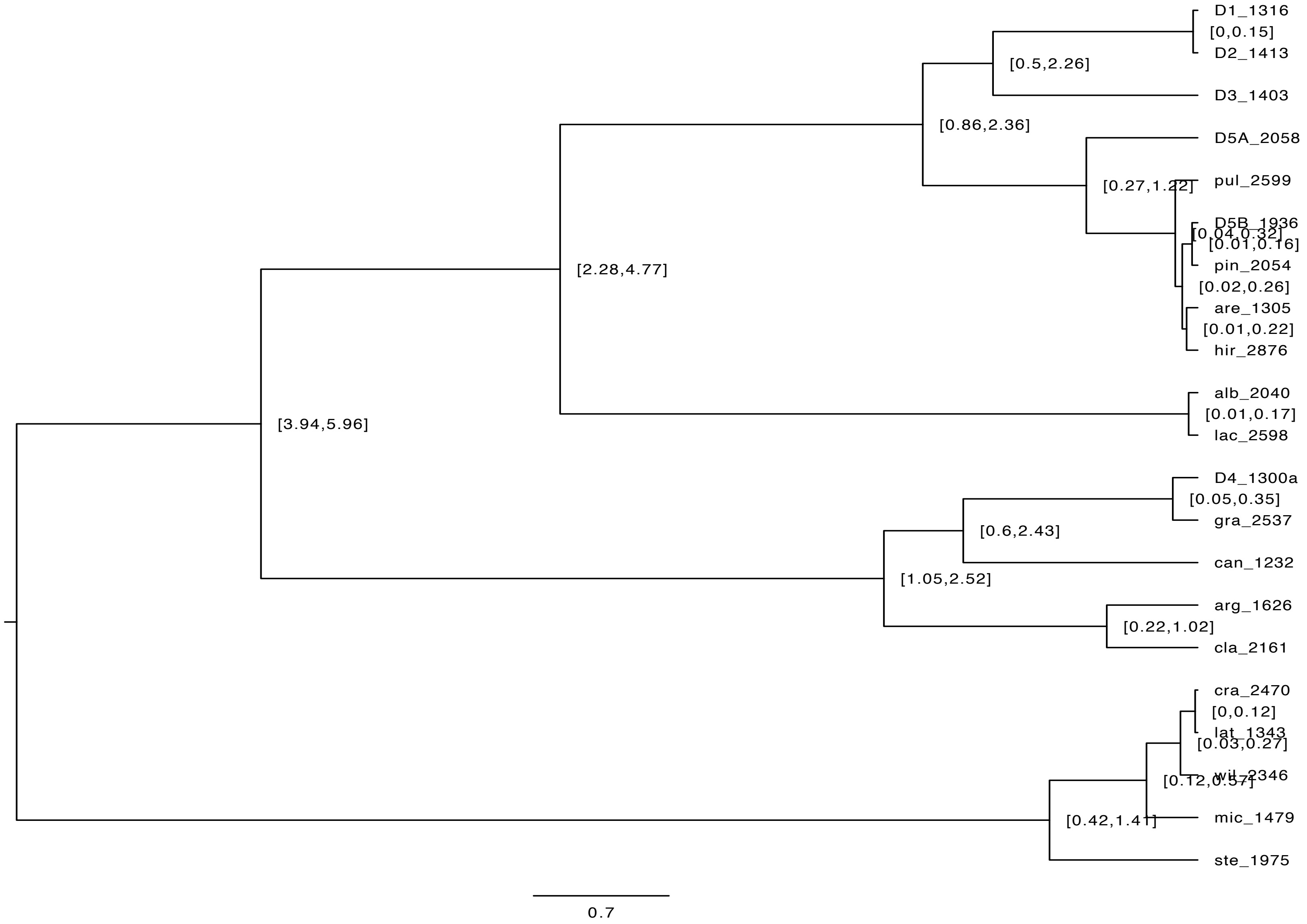

DOI 10.1002/aps3.11611

15% all SNP filtering threshold RelTime

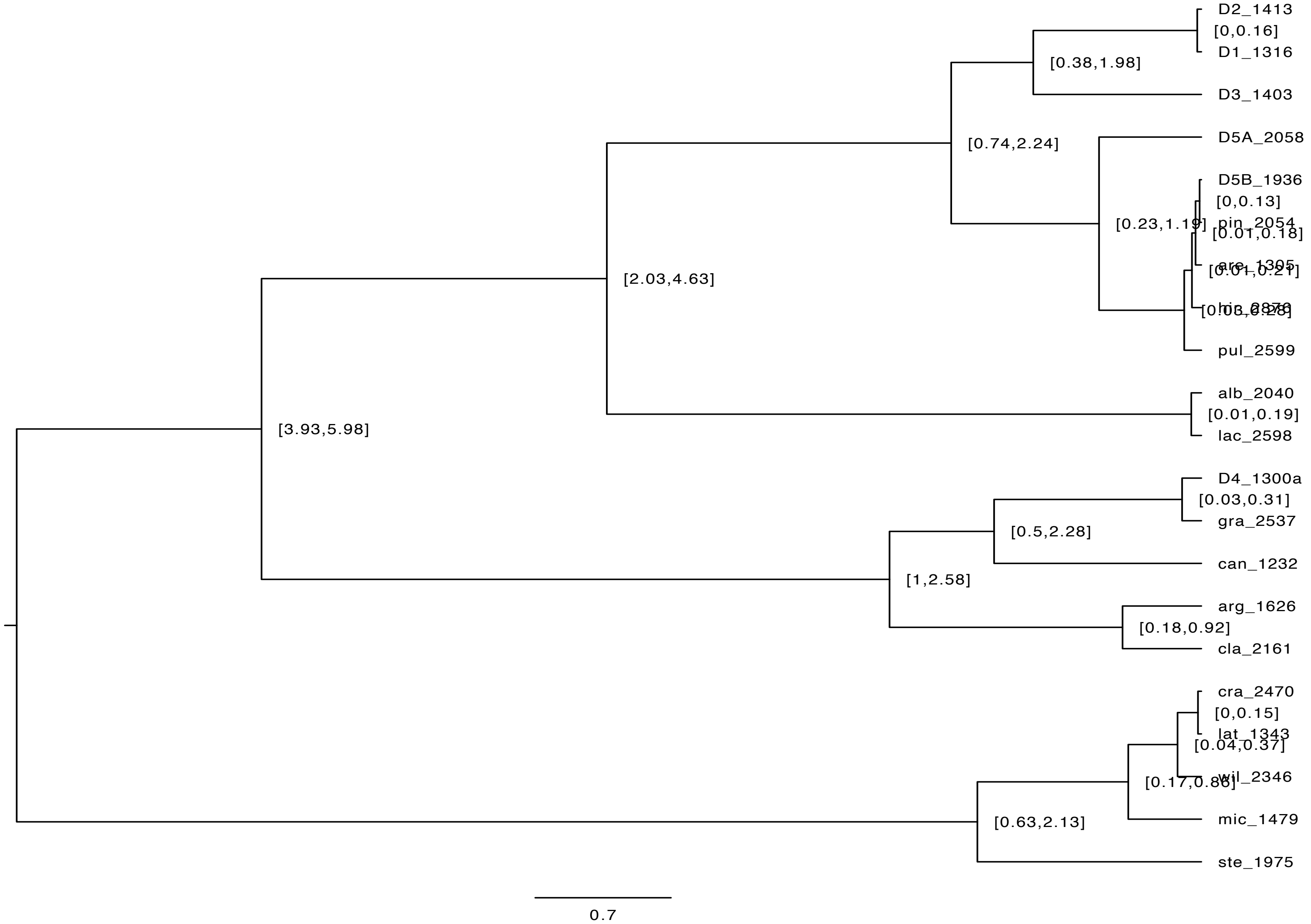

DOI 10.1002/aps3.11611

15% variant SNP filtering threshold RelTime

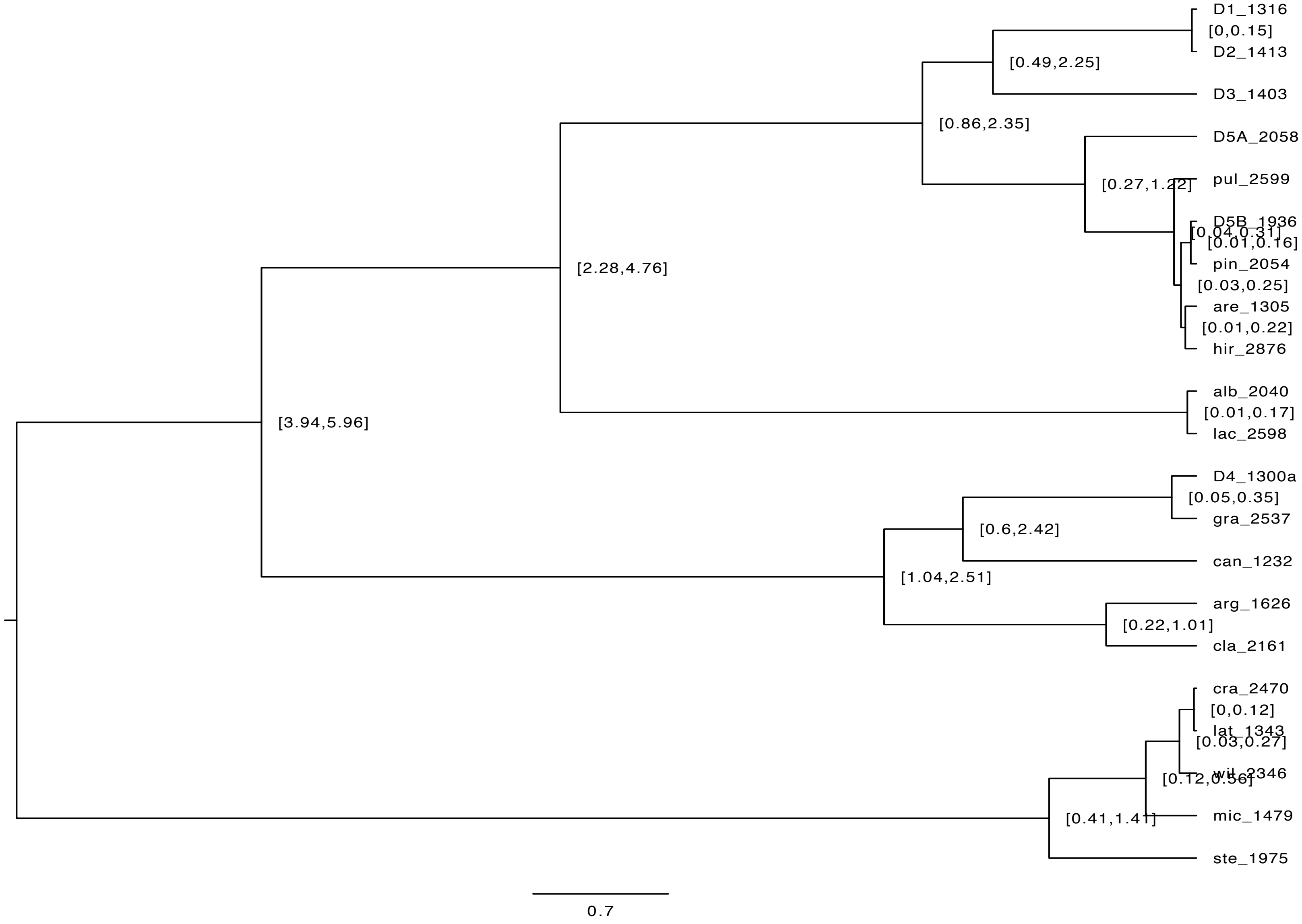

DOI 10.1002/aps3.11611

30% all SNP filtering threshold RelTime

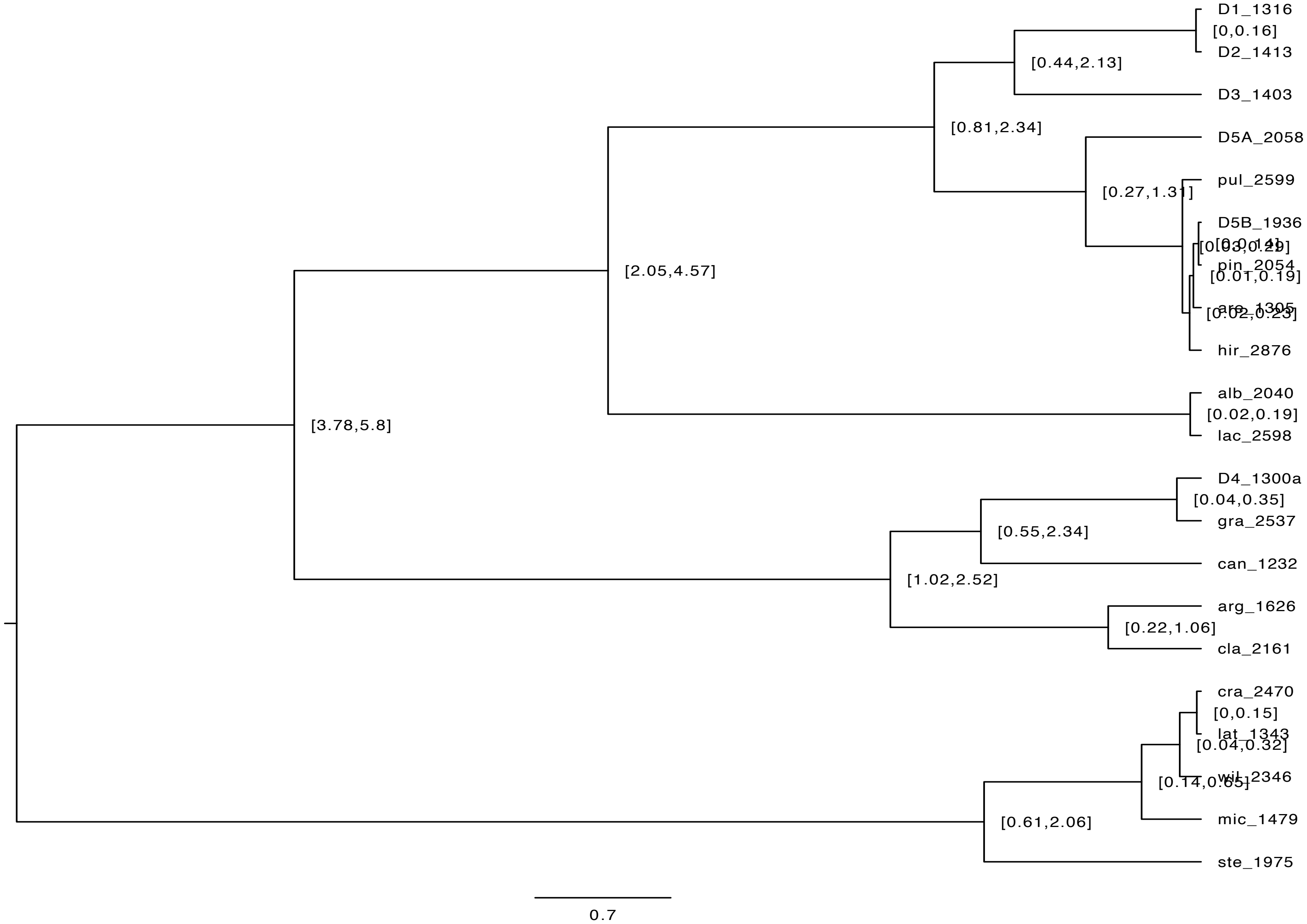

DOI 10.1002/aps3.11611

30% variant SNP filtering threshold RelTime

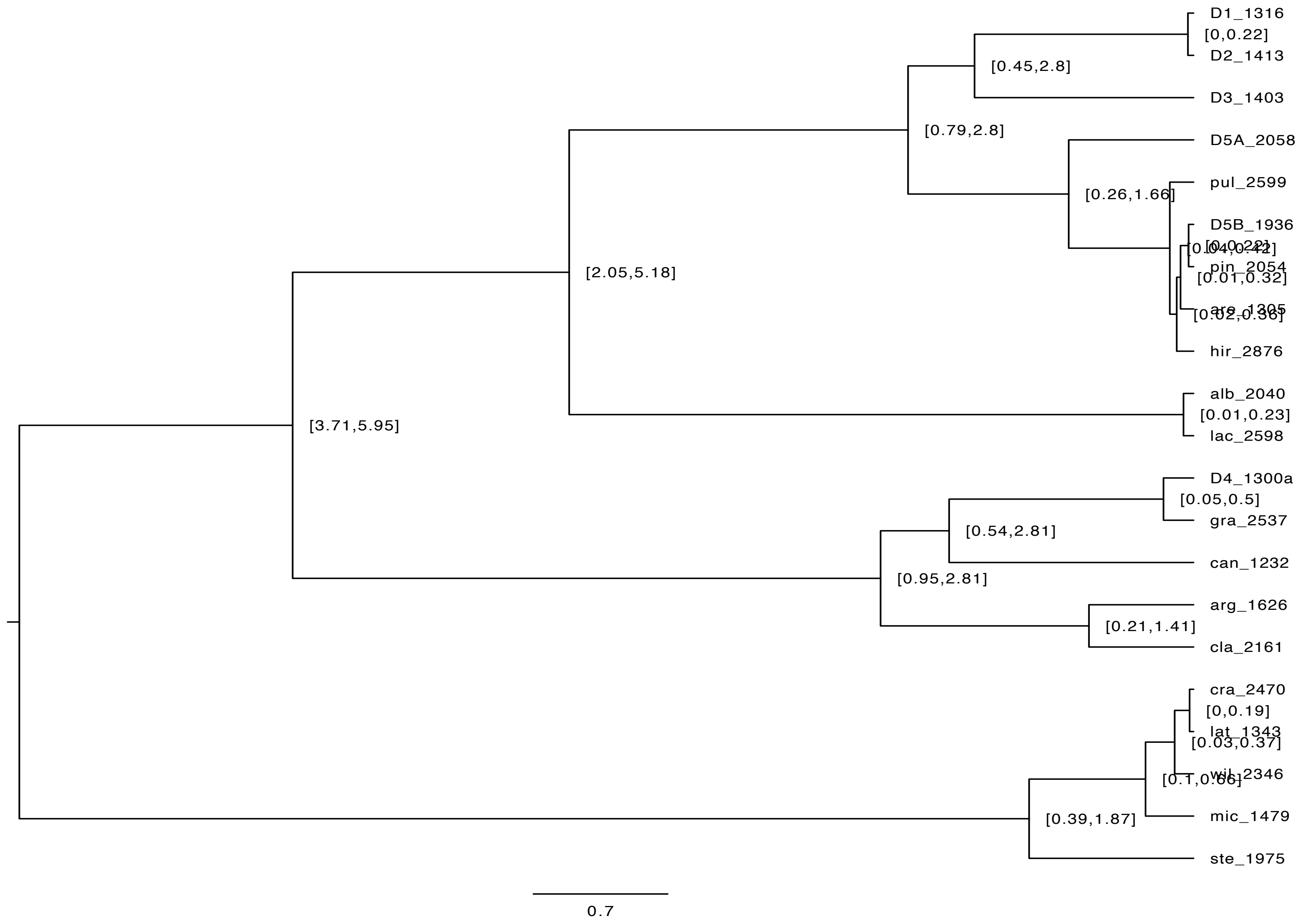

DOI 10.1002/aps3.11611

45% all SNP filtering threshold RelTime

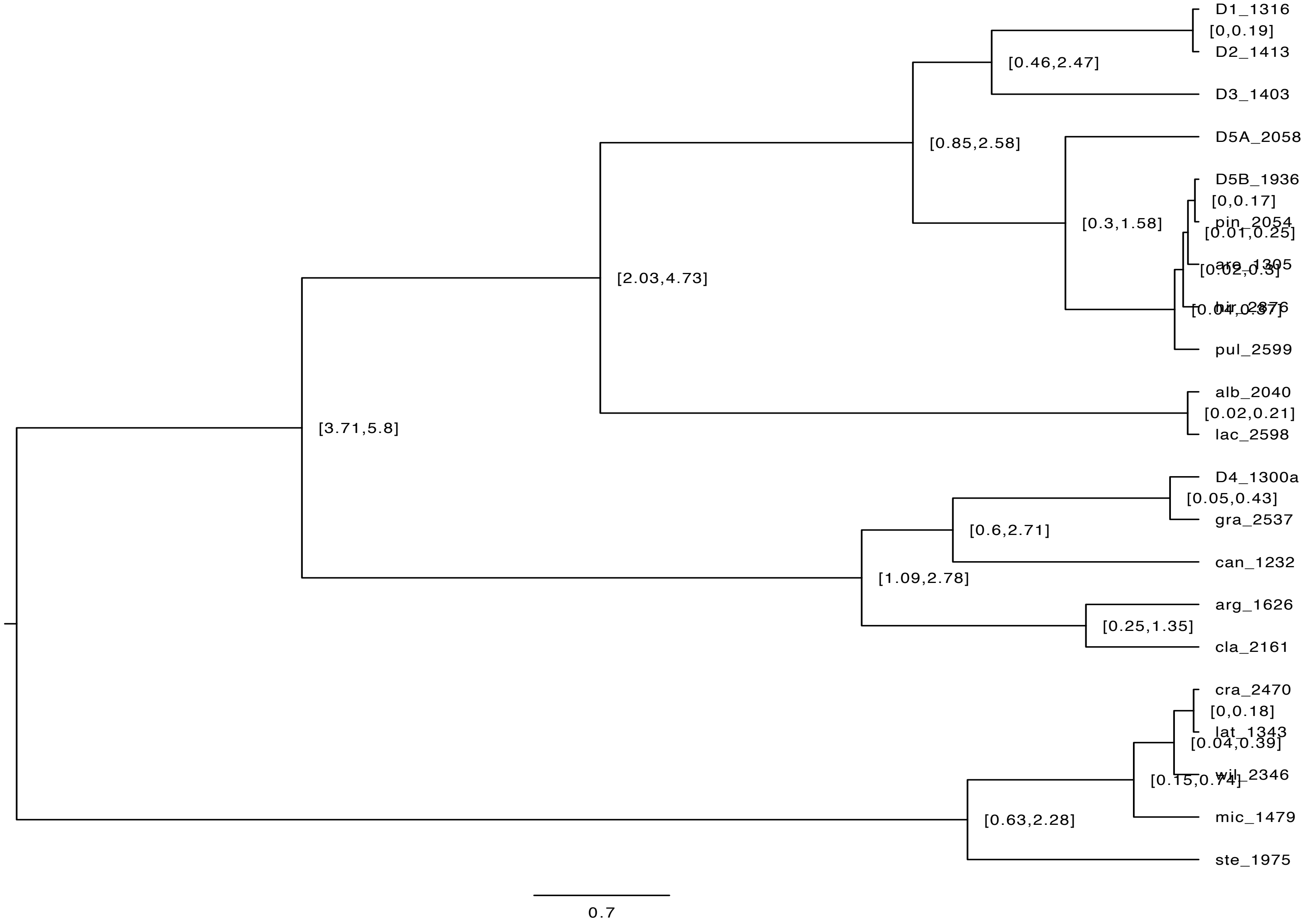

DOI 10.1002/aps3.11611

45% variant SNP filtering threshold RelTime

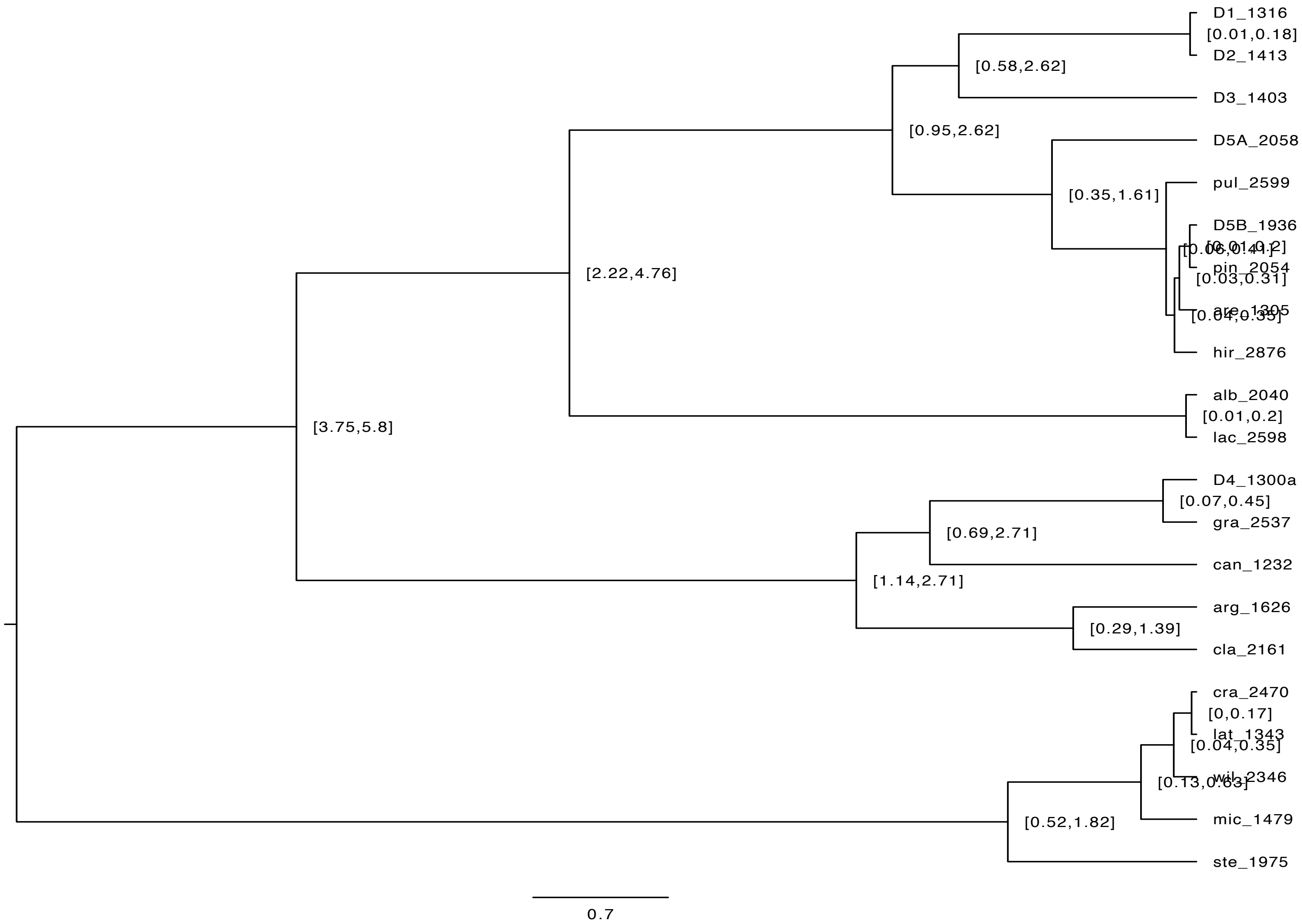

DOI 10.1002/aps3.11611

60% all SNP filtering threshold RelTime

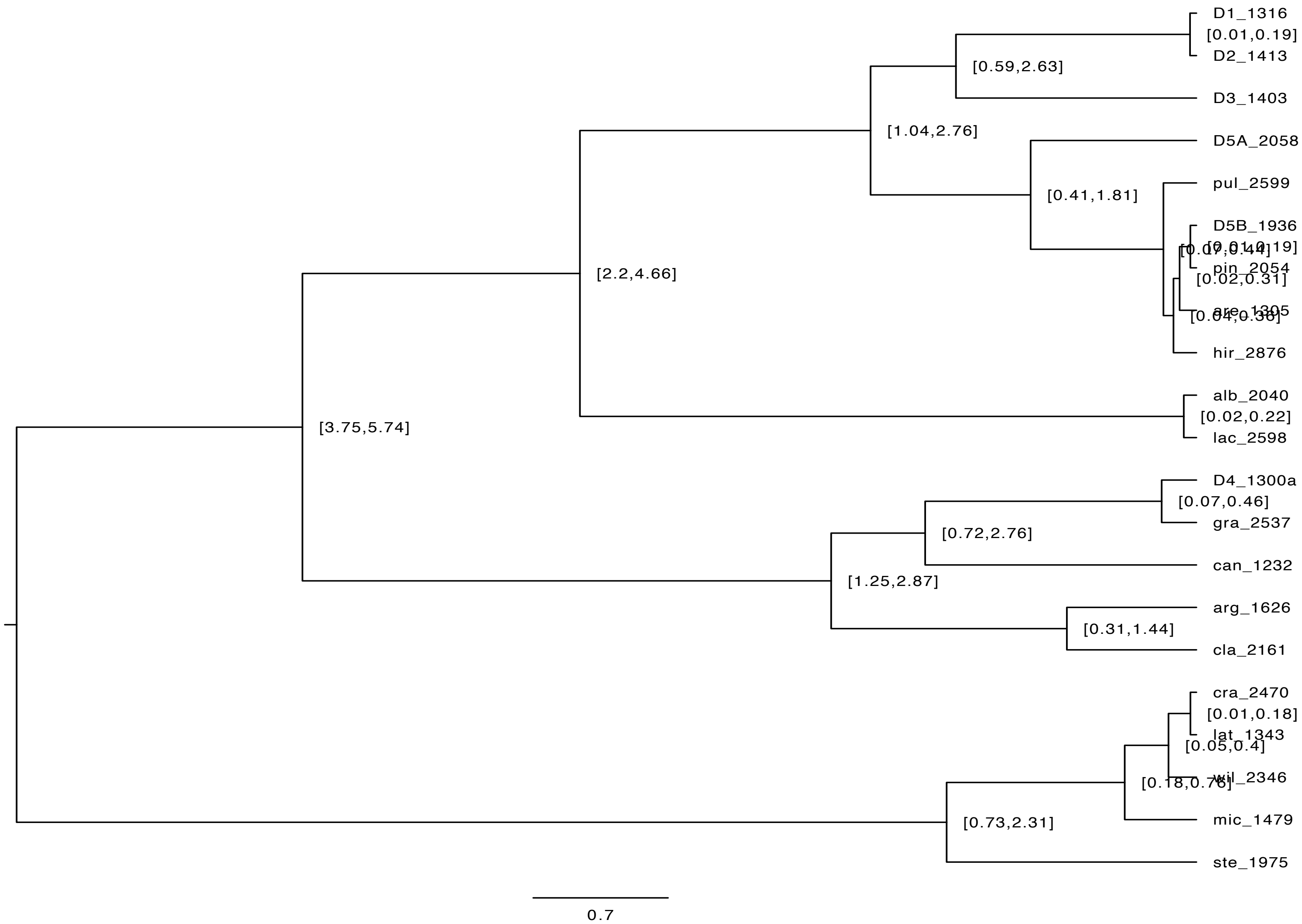

DOI 10.1002/aps3.11611

60% variant SNP filtering threshold RelTime

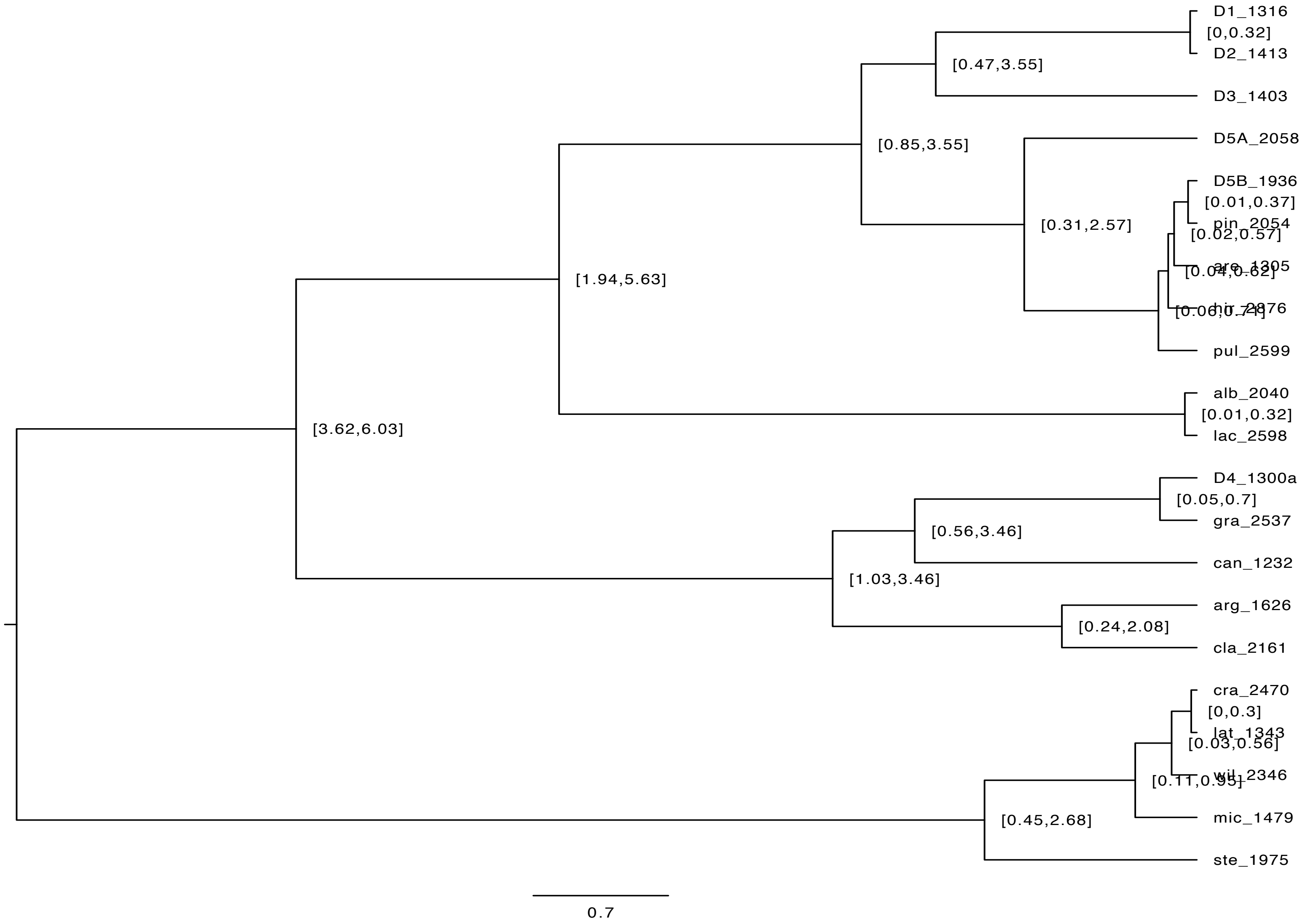

DOI 10.1002/aps3.11611

75% all SNP filtering threshold RelTime

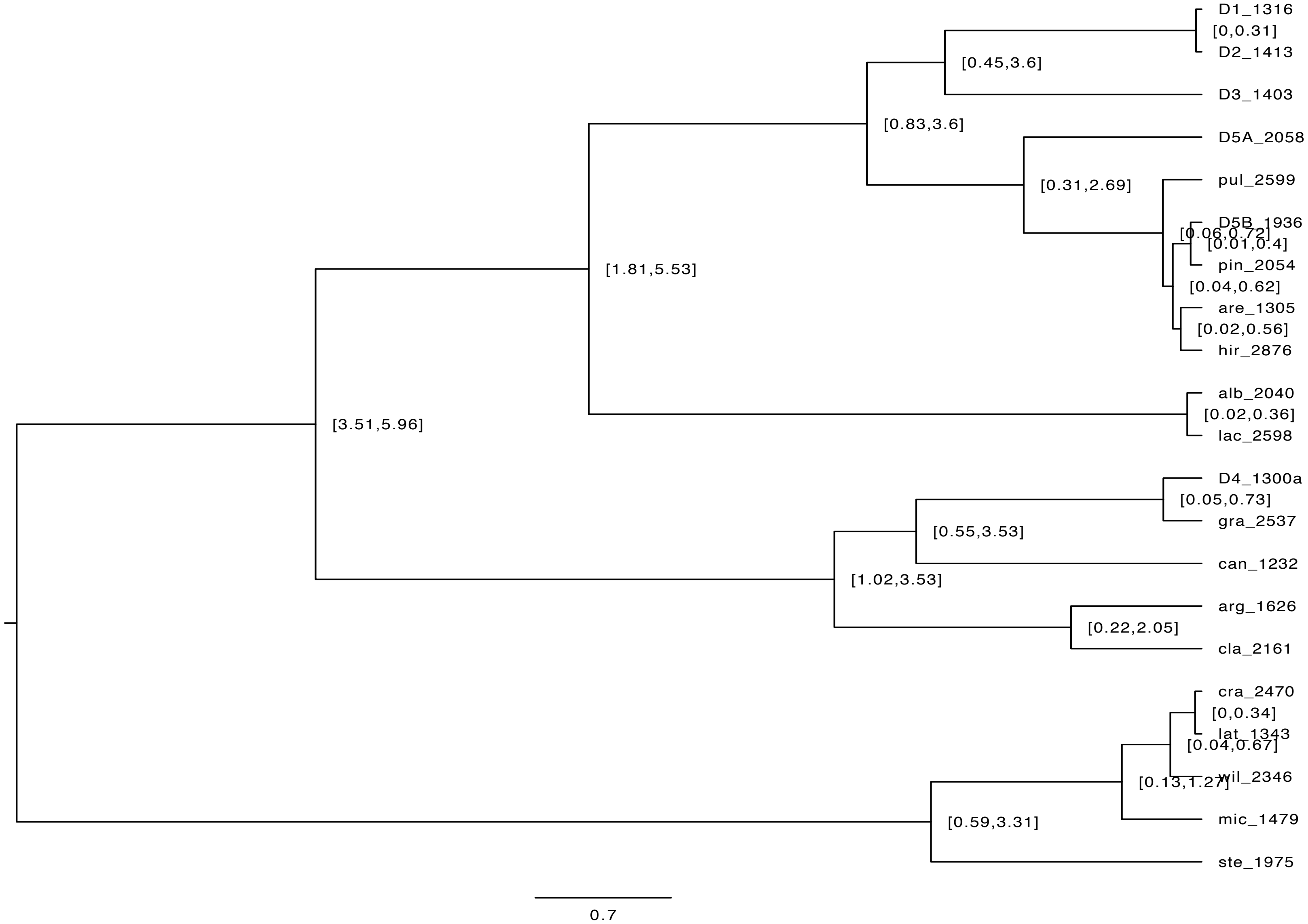

DOI 10.1002/aps3.11611

75% variant SNP filtering threshold RelTime

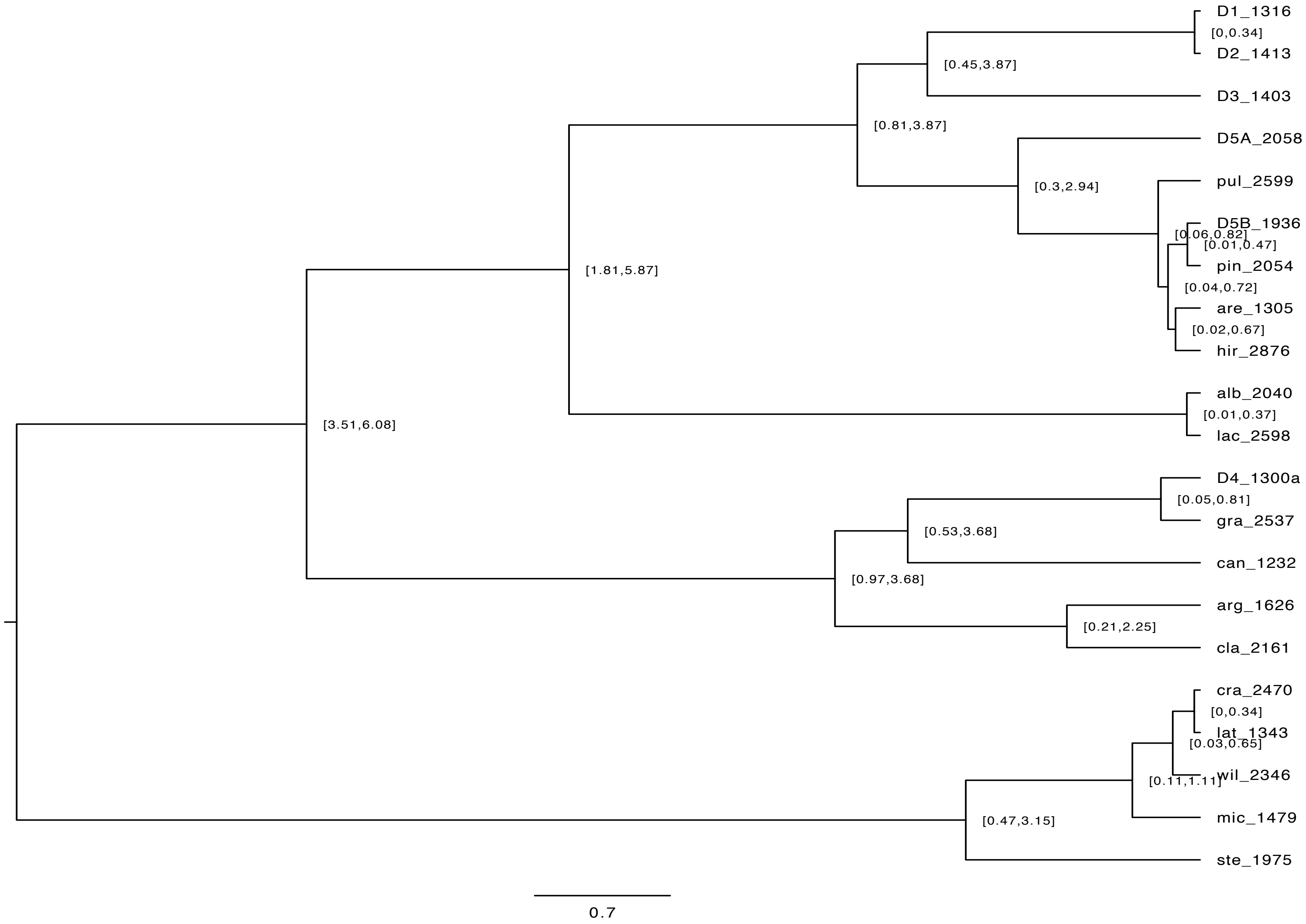

DOI 10.1002/aps3.11611

90% all SNP filtering threshold RelTime

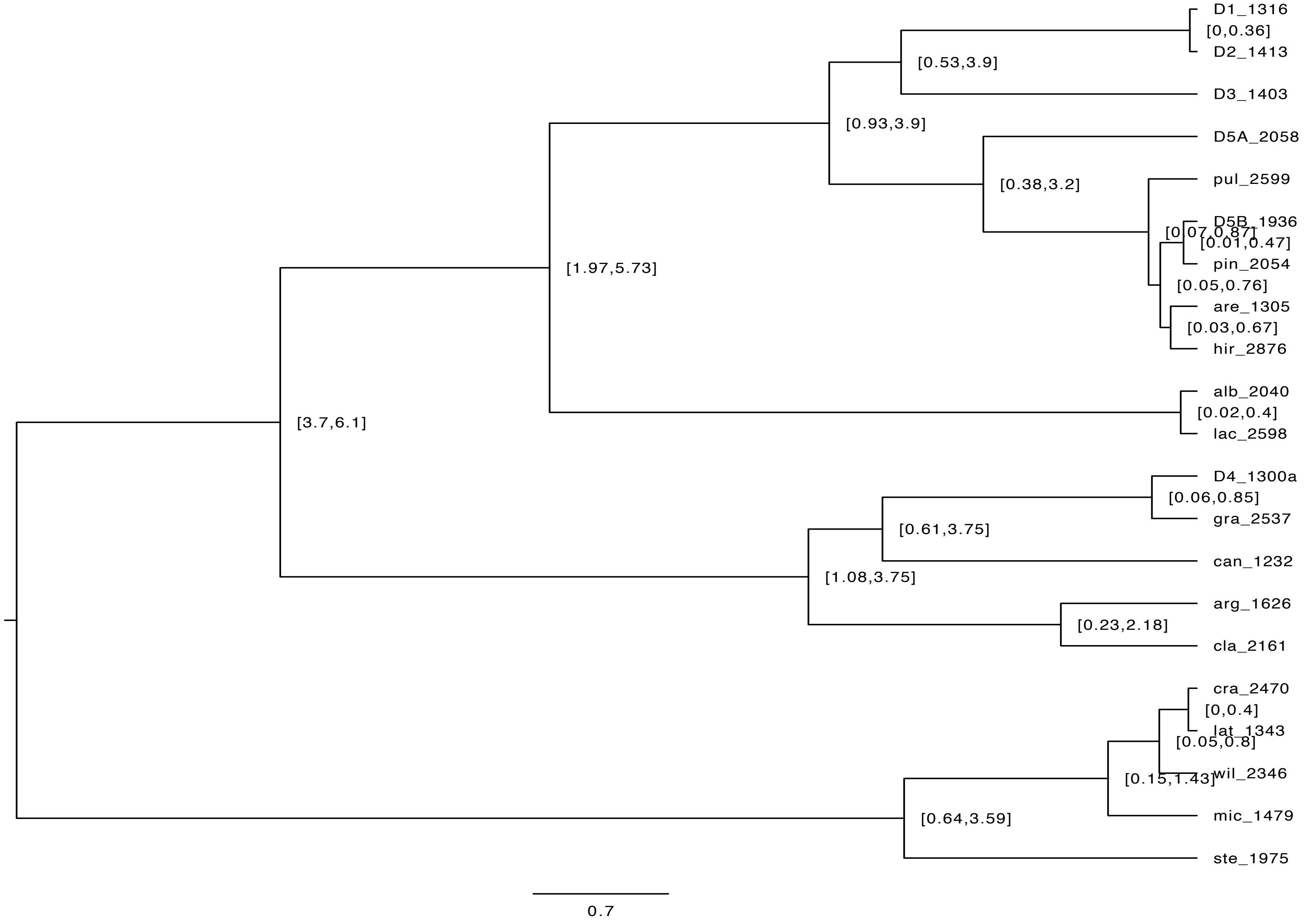

DOI 10.1002/aps3.11611

90% variant SNP filtering threshold RelTime

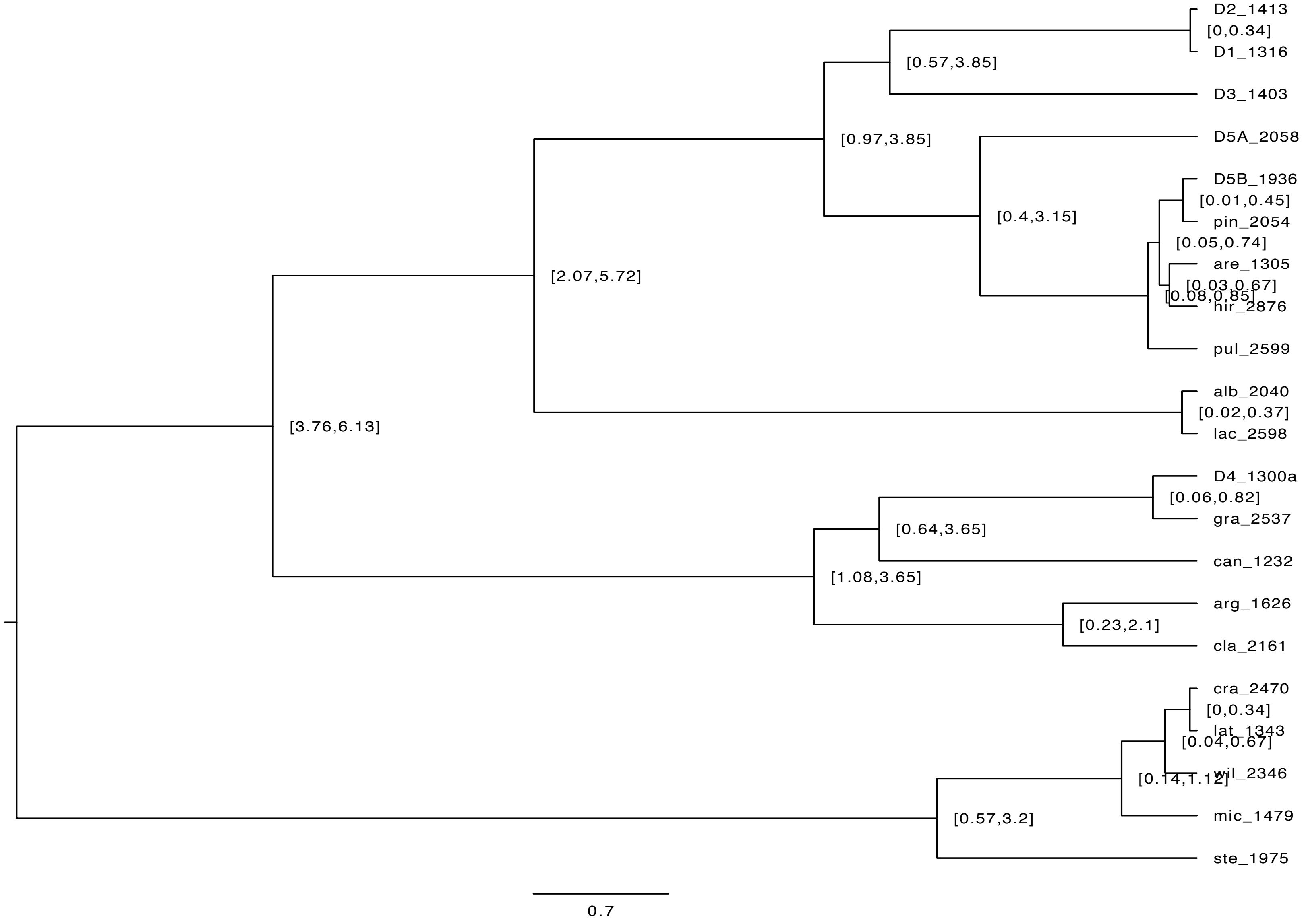

DOI 10.1002/aps3.11611

100% all SNP filtering threshold RelTime

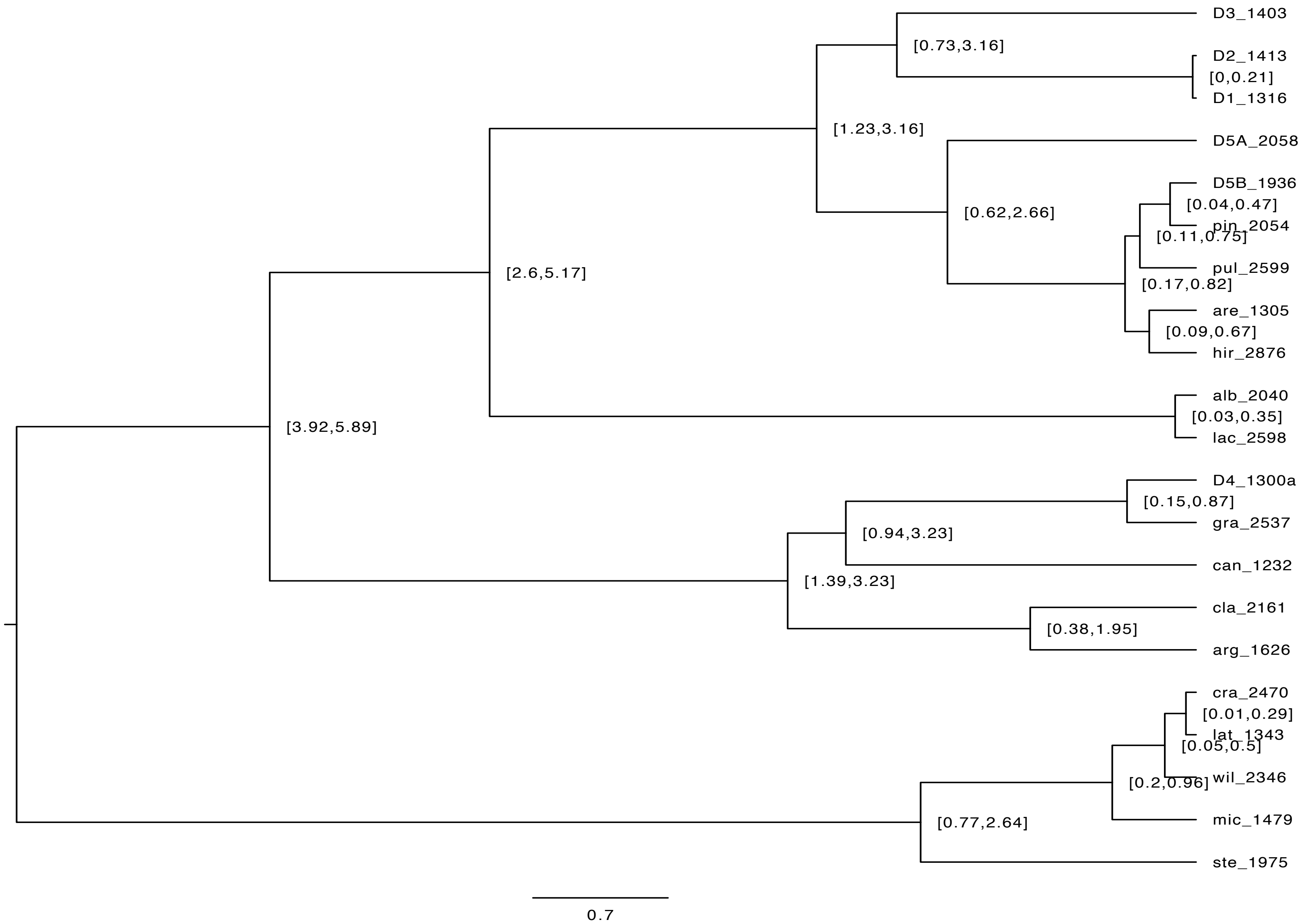

DOI 10.1002/aps3.11611

100% variant SNP filtering threshold RelTime

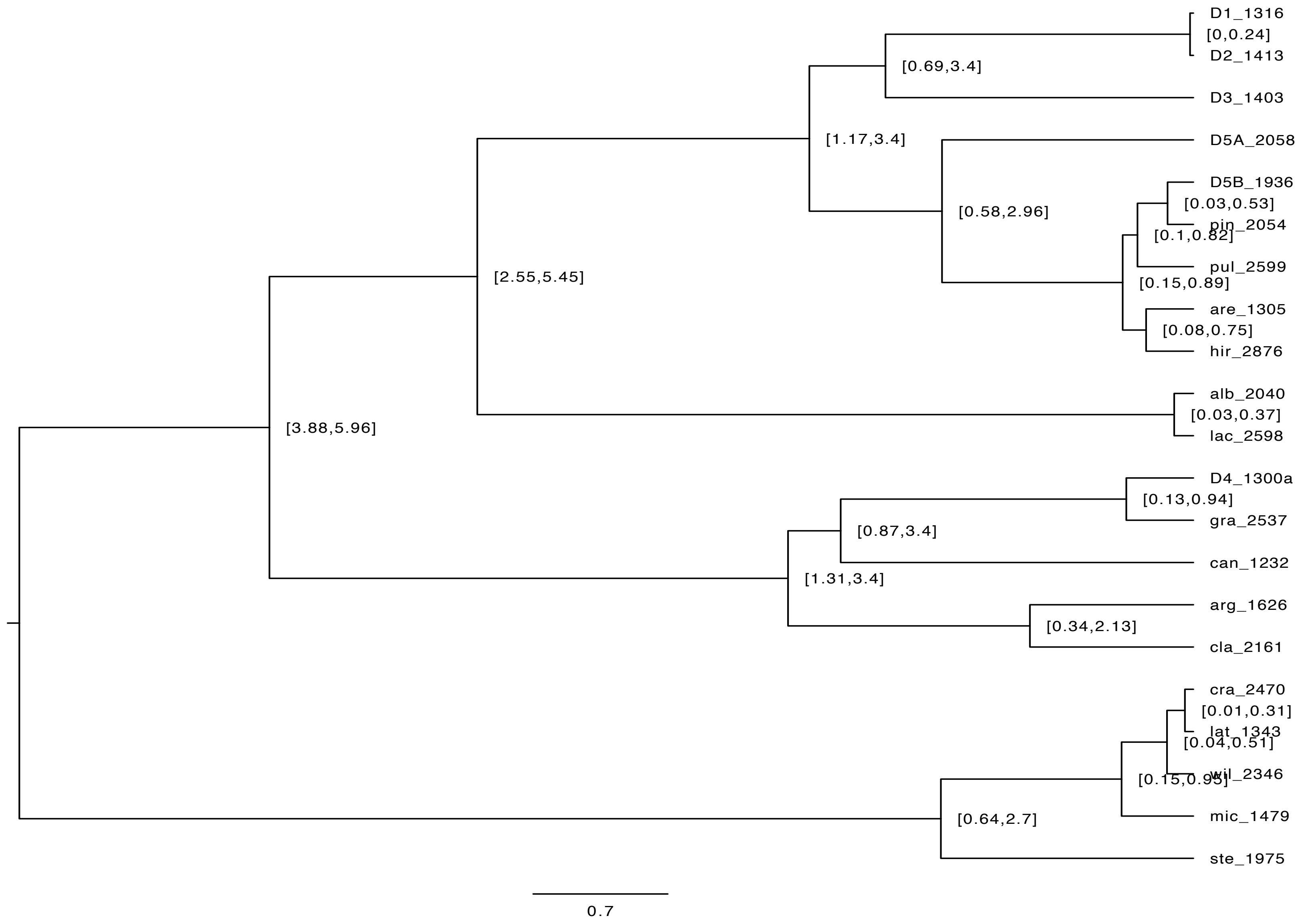

Supplement: Supplementary file 6 — Appendix S6. Inferred topologies and divergence time estimation with the 95% confidence interval of node ages from all empirical datasets including different filtering thresholds and analysis methods. Divergence time estimation in RelTree was rooted with Glycine falcata as in the BEAST analyses, but due to plotting limitations of Mega, the outgroup was not included in the plot. [file APS3-12-e11611-s004.pdf]
